# Deconvolution of transcriptomes and miRNomes by independent component analysis provides insights into biological processes and clinical outcomes of melanoma patients

**DOI:** 10.1186/s12920-019-0578-4

**Published:** 2019-09-18

**Authors:** Petr V. Nazarov, Anke K. Wienecke-Baldacchino, Andrei Zinovyev, Urszula Czerwińska, Arnaud Muller, Dorothée Nashan, Gunnar Dittmar, Francisco Azuaje, Stephanie Kreis

**Affiliations:** 1 Quantitative Biology Unit, Luxembourg Institute of Health (LIH), L-1445 Strassen, Luxembourg; 2 Life Sciences Research Unit (LSRU), University of Luxembourg, L-4367 Belvaux, Luxembourg; 3 Epidemiology and Microbial Genomics Unit, Department of Microbiology, Laboratoire National de Santé, Dudelange, Luxembourg; 4 INSERM, U900, F-75005 Paris, France; 5 MINES ParisTech, PSL Research University, F-75006 Paris, France; 6 Centre de Recherches Interdisciplinaires, Université Paris Descartes, Paris, France; 7 Klinikum Dortmund GmbH, 44137 Dortmund, Germany

**Keywords:** Independent component analysis, Deconvolution, Transcriptomics, Cancer, Survival analysis

## Abstract

**Background:**

The amount of publicly available cancer-related “omics” data is constantly growing and can potentially be used to gain insights into the tumour biology of new cancer patients, their diagnosis and suitable treatment options. However, the integration of different datasets is not straightforward and requires specialized approaches to deal with heterogeneity at technical and biological levels.

**Methods:**

Here we present a method that can overcome technical biases, predict clinically relevant outcomes and identify tumour-related biological processes in patients using previously collected large discovery datasets. The approach is based on independent component analysis (ICA) – an unsupervised method of signal deconvolution. We developed parallel consensus ICA that robustly decomposes transcriptomics datasets into expression profiles with minimal mutual dependency.

**Results:**

By applying the method to a small cohort of primary melanoma and control samples combined with a large discovery melanoma dataset, we demonstrate that our method distinguishes cell-type specific signals from technical biases and allows to predict clinically relevant patient characteristics. We showed the potential of the method to predict cancer subtypes and estimate the activity of key tumour-related processes such as immune response, angiogenesis and cell proliferation. ICA-based risk score was proposed and its connection to patient survival was validated with an independent cohort of patients. Additionally, through integration of components identified for mRNA and miRNA data, the proposed method helped deducing biological functions of miRNAs, which would otherwise not be possible.

**Conclusions:**

We present a method that can be used to map new transcriptomic data from cancer patient samples onto large discovery datasets. The method corrects technical biases, helps characterizing activity of biological processes or cell types in the new samples and provides the prognosis of patient survival.

## Background

Genomic and transcriptomic research has accumulated a vast collection of publicly available cancer related data. Data have been continuously collected using massive financial and scientific efforts. For example, The Cancer Genome Atlas (TCGA, https://www.cancer.gov/tcga) holds over 10,000 patient-derived samples including various levels of omics data: DNA, RNA, and proteins. Now, the question arises if these resources can also be used to support clinicians in making rapid and accurate assessments leading to tailored treatments for individual cancer patients. Integrating this information still poses a considerable obstacle as genomic and transcriptomic data from cancer patients are characterised by significant heterogeneity at three levels. First, results are generally collected using different sample preparation protocols and transcriptome analysis platforms and are then interrogated by constantly changing techniques. Although these techniques have improved on accuracy, sensitivity or genome coverage, they restrain backward compatibility, e.g., expression level analysis has evolved from qPCR through microarrays toward NGS sequencing in the last 15 years. Second, the data are collected for various layers of “omics”: genome, transcriptome, miRNome, proteome etc. Integration of data from these layers is not trivial especially when genomically unconnected entities should be integrated, like microRNA and their target mRNAs. Third, collected patient samples are intrinsically heterogeneous at tissue and cellular levels. Bulk analysis of transcriptomes can mask different types of heterogeneity in the sample as tumour biopsies contain many cell types that are mixed in different proportions [1]. Furthermore, there are well-documented variations of tumour cells within the same neoplasia, which can conceal low abundant, but critical cell subtypes such as drug-resistant tumour cells [2]. These facts limit discoveries and can lead to erroneous clinical conclusions [3, 4]. The experimental approach to resolve the complex issue of working with heterogeneous cancer samples involves physical separation of tissue into homogeneous cell populations or even single cells (by cell sorting, single cell technologies or microdissection) before the actual measurement. Technologically, this is an expensive and laborious task, which is not yet accessible routinely and which can introduce experimental errors [5, 6].

Alternatively, computational approaches can be applied to separate or deconvolute multivariate signals from different cell types, accounting for variable biopsy sample composition and intra-tumour heterogeneity [7–10]. One of the most promising methods of assumption-free transcriptome deconvolution is independent component analysis (ICA) [11]. This method originated from the domain of signal processing aiming at detecting individual components from a complex mix of mutually independent non-Gaussian signals. It allows to identify sources of transcriptional signals, cluster genes into functional groups and cell type-related signatures [10, 12, 13] and deduce interactions between biological processes [14]. The method can also recognise and remove biologically irrelevant biases introduced by different measurement platforms [15]. Therefore, this approach can use pre-existing data that were collected through different stages of technological progress.

Here we present an ICA-based method combining newly measured data with pre-existing large discovery data. We show its prognostic power and the ability to characterize biological processes on the example of cutaneous melanoma patients.

Melanoma arises through the malignant transformation of melanocytes and presents a very aggressive form of skin cancer with increasing global case numbers. Melanoma’s extremely high mutation rate (> 10 somatic mutations/Mb) and the concomitant genetic heterogeneity make it difficult to distinguish true cancer driver genes from noise in bulk samples using current technologies [16, 17]. Nevertheless, the analysis of gene expression data resulted in three patient subtypes or clusters: “immune”, “keratin” and “MITF-low”, which have implications for patient survival [18]. Interestingly, the majority of primary melanomas belonged to the “keratin” cluster having a worse prognosis than the other two subtypes.

In this study, we used the skin cutaneous melanoma (SKCM) TCGA cohort with over 470 patients diagnosed with cutaneous melanoma as the discovery dataset. Two layers of “omics” data were considered and integrated: mRNA and microRNA (miRNA). The investigation dataset included a small cohort of three primary melanoma tumours and two controls: matched cancer patient-derived normal skin and normal melanocytes. First, for the discovery cohort, we demonstrated that ICA deconvolution can be successfully applied to classify patients based on their tumour subtypes and to build the risk score that predicts patient survival. The risk score was then tested using an independent validation cohort of 44 patients, obtained by microarray gene expression technology. The strong technical differences between discovery RNA-seq data and microarray-derived validation datasets were resolved by our method. Next, the investigation dataset was studied in depth and key processes involved in cancer aetiology were detected and quantified: immune response and inflammation, angiogenesis, self-sufficient cell proliferation among others.

We show here that consensus ICA can integrate data from different sources and platforms and predict clinically important characteristics of cancer in a bias-free, unsupervised and potentially automatable fashion, suggesting consensus ICA as a useful module of future clinical support systems.

## Methods

### Discovery, validation and investigation datasets

#### Discovery and validation datasets

As a discovery dataset, we used two SKCM TCGA datasets: RNA-seq (472 samples) and miRNA-seq (452 samples) data from the Genomic Data Commons (GDC) data portal of the National Cancer Institute of the National Institutes of Health (NIH, https://portal.gdc.cancer.gov/). Soft filtering as in [19] was used to reduce number of RNA features from 60,446 to 16,579 (see Additional file 3: Fig. S1): only genes with more than 1000 counts in at least one sample among 472 were considered. For miRNA we used less strict filtering and required at least one read to be presented. Four metrics of gene expression were considered for mRNA: raw counts, DESeq2-normalized counts [20], FPKM and TPM. All expression values were log2 transformed.

From TCGA clinical data we extracted survival time, gender and sample type (primary tumour or metastatic). We also added data of tumour subtype based on RNA-cluster (immune, keratin, MITF-low) as it is relevant for prognosis [18]. The extracted survival and clinical datasets are provided in Additional file 4: Tables S1 and S2, respectively.

A validation gene expression dataset was taken from [21], available from ArrayExpress under E-GEOD-19234. This microarray dataset consists of 44 metastatic samples from melanoma patients accompanied by survival information. The samples were collected from different metastatic sites, mainly from lymph nodes, from patients with grade III (39 samples) and IV (5 samples). As microarray expression data have very different dynamic range compared to RNA-seq [22], array expression were linearly transformed to fit RNA-seq distribution as described in Additional file 1: Supplementary Methods.

#### Investigation dataset

The investigation dataset, represented by RNA-seq and miRNA qPCR array data, originated from three primary tumour samples of melanoma patients (entitled P2PM, P4PM, P6PM) and two control samples: one matched normal skin P4NS and a healthy melanocyte cell line NHEM (see Additional file 4: Table S3).

Melanoma biopsies of three Caucasian patients were collected after surgical resection at the Dermatology Department of the University Clinic Freiburg, Germany. All patients signed an informed written consent. Ethical approval of this study was obtained from the Comité National d’Ethique de Recherche Luxembourg (CNER-No. 201201/05) and from the German Ethik-Kommission der Albert-Ludwigs-Universität Freiburg (EK-Freiburg 196/09). Histological examination and estimation of the percentage of tumour cells within the lesion was performed by two independent pathologists (normal skin and NHEM cell line were free of tumour cells). Tissues from snap frozen biopsies were lysed in RLT buffer with a Qiagen TissueLyser (50 Hz, 5 min). DNA and total RNA were extracted using the Qiagen’s AllPrep Mini Kit according to supplied protocols. Quality and quantity of samples were measured with Nanodrop, gel electrophoresis and Qubit High Sensitivity Kit. RNA integrity was determined using the Agilent Bioanalyzer Nano chip.

RNA-seq data for these samples are available by GEO accession number GSE116111 and Ct-values for all quantified miRNAs are available in Additional file 4: Table S4. MiRNA names were harmonised using miRBase v.21 and Ct-values were inverted and expression was calculated as 36-Ct.

### Data analysis

#### Consensus ICA

ICA was applied to the combined discovery and investigation datasets for unsupervised separation of signals and feature extraction (Additional file 3: Fig. S2 and S3). By combining the datasets, we expect that technical biases between the discovery and investigation data are estimated by the method and isolated within some of the components. Each layer of omics data: mRNA and miRNA was analysed separately at this stage. ICA implementation from the `fastICA` package of R was used [23]. Let us denote ***E***_***nm***_ the expression matrix of n genes or miRNAs measured in m bulk samples. ICA decomposed such a matrix into a product of k statistically independent transcriptional signals ***S***_***nk***_ (addressed as matrix of metagenes) and a weight or mixing matrix ***M***_***km***_ (matrix of metasamples) [11].

1 $$ {\boldsymbol{E}}_{\boldsymbol{nm}}={\boldsymbol{S}}_{\boldsymbol{nk}}\times {\boldsymbol{M}}_{\boldsymbol{km}} $$

The values represented in the columns of ***S*** (metagenes) can be interpreted as the level of influence of the corresponding genes/miRNAs on the components and considered as “markers” of the component. Weights in rows of ***M*** show how the metagenes are mixed in the samples. In order to distinguish independent components obtained after ICA of mRNA and miRNA data, we introduce the terms RICs (mRNA) and MICs (miRNAs). Thus, each RIC and MIC is associated with two vectors: one showing the contribution of the genes into this component (a column of ***S***); the second representing the weights of the component in the samples (a row of ***M***). Unlike non-negative matrix factorization, both metagenes and weights can be positive or negative and ab initio the selection of the direction is random, depending on the initial estimation. ICA may also suffer from reduced reproducibility for at least some components. To mitigate these drawbacks, we ran the analysis multiple times (100 runs during the exploratory steps and 1000 for the final analysis) following [13]. In brief, the algorithm used for consensus ICA is described below.

- For the defined number of tries (*nt*), a random sample was excluded from the expression matrix and ICA was performed on this reduced dataset. As a result, we obtained *nt* matrices ***M***^***(1)***^ and ***S***^***(1)***^.
- Next, one of the decompositions was selected as “standard” and all the others were compared to it by correlation of metagenes. The sign and order of components was adjusted to fit the “standard” decomposition.
- Consensus ***S*** and ***M*** matrices were calculated by averaging all reordered ***S***^***(1)***^ and ***M***^***(1)***^. The squared correlation between corresponding metagenes was used as a measure of stability (*R*^2^).

Multithreading was implemented in R code to speed-up calculations using the `foreach` package and either `doMC` (Linux) or `doSNOW` (MS Windows) packages available in R/Bioconductor. The script of the implemented consensus ICA and following analysis (Additional file 2: Supplementary Results) is available online: https://gitlab.com/biomodlih/consica.

#### Gene signatures and functional annotation

The top-contributing genes and miRNAs per component were detected using the following significance analysis approach. A *p*-value was individually assigned to each gene/miRNA within each component, based on the probability that it came from a normal distribution with estimated parameters. As the ICA algorithm extracted non-Gaussian signals from the mixed data, the contributing genes that did not deviate from the normal distribution were considered as non-important. In most components, there was a small subset of genes that had extremely high absolute values in ***S***, while the majority was normally distributed. To avoid overestimation of the variances, we used non-parametric measures of the centre and scale: median and median absolute deviation. Then these *p*-values were adjusted for multiple testing (Benjamini & Hochberg), and genes with an adjusted *p*-value (adj.*p*-value) < 0.01 were reported as top-contributing (see Additional file 2: Supplementary Results). Two lists of top-contributing genes resulted from the analysis – positively and negatively involved. The lists of top-contributing genes of each RIC were afterwards used for over-representation (enrichment) analysis. The 16,579 genes, with expression above the selected threshold in at least one sample, were used as a background gene list and significantly enriched (adj.*p*-value< 0.01) GO terms were investigated. In order to simplify the interpretation and to increase the robustness for runs on different datasets, we reoriented the components in order to have the most significantly enriched categories associated with positive top-contributing genes (see Additional file 1: Supplementary Methods). For MICs, the direction could not be identified by enrichment analysis, therefore we reoriented only those MICs that showed strong negative correlation with RICs.

#### Prediction of sample classes

Random forest classifier, implemented in the `randomForest` R-package [24], was used with the default settings to predict classes of patients. Columns of the weight matrix ***M*** were used as inputs and clinical variables (e.g. gender, sample type) as outputs. Each variable was analysed independently. First, leave-one-out cross-validation (LOOCV) was performed on the discovery set in order to address the ability of predicting sample classes and estimate the accuracy of prediction. Then the random forest, trained on all discovery data, was used to predict classes for the new clinical samples of the investigation dataset. To ensure accuracy and robustness of our approach to select the number of components, we performed a nested cross-validation, excluding 20% of the data and using the remaining 80% to estimate the optimal number of components and then train the classifier (Additional file 1: Supplementary Methods).

#### Other dimensionality reduction methods

In order to compare the performance of consensus ICA to other available tools, we run benchmarking of 7 approaches, applying them to mRNA expression data. First, we considered standard PCA of the joint dataset and PCA after correction for batch effects between discovery and investigation data sets using ComBat (package `sva`) [25] and XPN [26]. Next, we applied the non-negative matrix factorization (NMF) implemented in the `NMF` package [7] and low-rank approximation based multi-omics data clustering (LRAcluster) [27]. Finally, we investigated several non-linear dimensionality reduction methods, such as locally-linear embedding (LLE) implemented in the `lle` package [28], Isomap (package `RDRToolbox`) [29], as well as t-SNE (package `tsne`) [30]. To compare these methods to ICA, we performed 20 runs of 5-fold cross validation and estimated the accuracy of patient classification by random forest. For fair comparison, we used 80 features (dimensions), the same as number of components for ICA. For PCA, we pre-selected 80 principal components with the highest predictive power.

#### Integration of components for survival prediction

Weights of the components (rows of matrix ***M***) were statistically linked to patient survival using Cox partial hazard regression implemented in the `survival` package of R [31]. Adjusted *p*-values of the log rank test were used to select significant components. However, the prognostic power of each individual component might not have been high enough to be applied to the patients from the new cohort. Therefore, we integrated weights of several components, calculating the risk score (RS) with an improved prognostic power. For each patient, its RS is the sum of the products of significant log-hazard ratios (LHR) of the univariable Cox regression, the component stability *R*^2^ and the standardised row of weight matrix ***M***:

2 $$ {RS}_i=\sum \limits_{i=1}^k{H}_i{R}_i^2{M}_{i,j}^{\ast } $$

where *H*_*i*_ is LHR for the components significantly (adj.*p*-value< 0.05) linked to survival and 0 for other. The applicability of the proposed score was checked using the independent validation dataset. This dataset was extracted from another study and was based on an independent cohort of the patients [21]. In addition, gene expression for the validation cohort was measured using Affymetrix U133 Plus 2.0 microarrays, while the discovery dataset was based on RNA-seq.

#### Biological relevance of the components

Our strategy to investigate the biological relevance of the components is presented in Additional file 3: Fig. S1 (green box). First, we attempted to connect the metagenes of all the components from the mRNA data to biological functions and cell types. We analysed separately the positively and negatively contributing genes using several tools. Automatic analysis was done by `topGO` R-package [32] followed by a manual analysis with Enrichr [33] that checked for enrichment in multiple categories originated from various databases (we used Reactome 2016, GO Biological Processes 2017, Human Gene Atlas, ARCHS4 Tissues and Chromosome Location). In addition, we compared the metagenes to the ones previously published by Biton et al. [10] and assigned the component number to the reciprocally corresponding metagene as explained in [34] using the `DeconICA` R-package (https://zenodo.org/record/1250070). As enrichment of immune-related processes and functions was observed, we also correlated our metagenes to the immune cell type signature matrix named LM22 [35] in order to identify components originated from different types of leukocytes; cell-types were associated with components through highest absolute Pearson correlation. Finally, for some components we confirmed their biological origin by correlating the metagenes with averaged gene expression profiles of cell types measured at a single-cell level and reported by Tirosh et al. [36]. For miRNA data we considered enrichment (hypergeometric test) of genomic locations of contributing miRNAs annotated by the cyto_convert tool of NCBI.

#### Integration of components for data at miRNA and mRNA levels

Pearson correlation between weights of the components was used to link the components found within mRNA and miRNA data. Here we hypothesized that if two components show significant correlation of the weights in all the samples, they should be functionally linked. Of note, these MICs have been linked to their respective RIC, purely based on the high absolute correlation of component weights, without considering any biological knowledge. Due to the lack of tools providing data with regard to biological functions or cell types for miRNAs, we performed literature mining, searching for all publications related to miRNAs-clusters and additional biologically relevant keywords. More detailed description of literature mining is given in Additional file 1: Supplementary Methods.

#### Involvement of components in the new samples

The involvement or the weight of each component in the samples is not centred and scaled due to the nature of ICA. Therefore, to visualize the involvement of the components in the new samples, we replaced the weights of the components by a ranking score that changed from 0 to 1 (only discovery data were considered to define the ranking). If the weight of the considered component in a new sample was below (or above) the weights in the discovery set, such component automatically was assigned to a limiting value of 0 (or 1). Values of ranking score around 0.5 in the new sample suggest that the weight of the considered component was close to the median in the discovery set.

## Results

### ICA of combined data sets can remedy technical biases

**Fig. 1:**
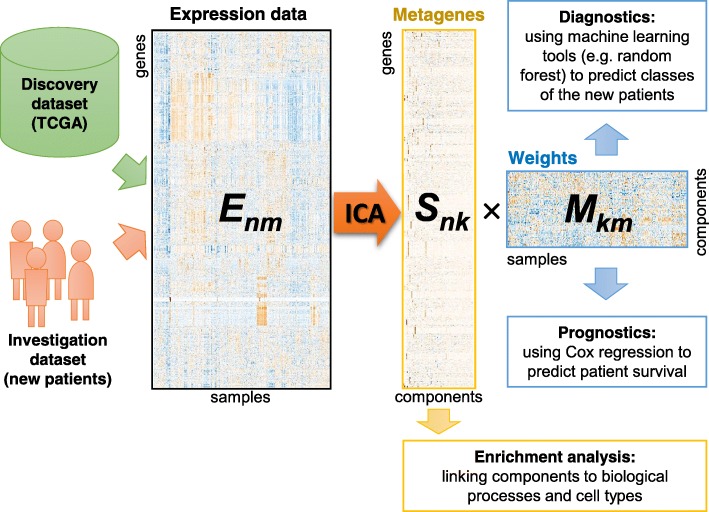
Visualization of the approach taken to data analysis. A large discovery dataset and a small investigation dataset from patients (both mRNA) were concatenated and analysed together by ICA. As a result, two matrices were obtained: ***S*** (metagenes), containing contribution of the genes to each component, and ***M*** (metasamples), presenting the weights of the components in the samples. ***S*** provides gene signatures for each of the components, which could be linked to cellular processes by standard functional annotation or enrichment analysis. ***M*** can be linked to clinical data and used to predict classes of new patients and their survival

In this study, graphically outlined in Fig. 1 (see detailed schemes in Additional file 3: Figs. S1 and S2), we used public TCGA data as the discovery dataset, published microarray data [21] as a validation set. An investigation data set was based on newly obtained clinical samples described in Methods and Additional file 4: Table S3. ICA was applied to two types of transcriptomic data: mRNA and miRNA expression. The number of components was chosen based on ability of ICA features to classify patients in the discovery set (see Additional file 1): 80 independent components were used for the deconvolution of mRNA data (named RIC1–80) and 40 for miRNA data (denoted as MIC1–40). ICA was run 1000 times in order to achieve robust results. 49 of RICs and 36 MICS showed high reproducibility (with stability of metagenes or mean *R*^2^ > 0.5). The values of *R*^2^ are provided in the Additional file 4: Tables S5 and S6. The improvements linked to the use of consensus ICA over single-run ICA were recently discussed in [34]. Here we independently investigated the effect of consensus on classification and reproducibility of the results. First, we compared accuracies obtained with several single ICA runs and the accuracy obtained using a consensus approach. We saw a slight, but statistically significant improvement for sample type (from 0.868 to 0.871, *p*-value = 6e-3) but not for tumour subtype (from 0.9 to 0.902, *p*-value = 0.39). At the same time, a much stronger effect was observed on the reproducibility of metagenes and gene signatures, associated with the components (see Additional file 3: Fig. S4 A,B). Therefore, the use of consensus ICA may be considered as optional for patient classification, but it is necessary for obtaining reproducible and biologically interpretable components and gene signatures.

**Fig. 2:**
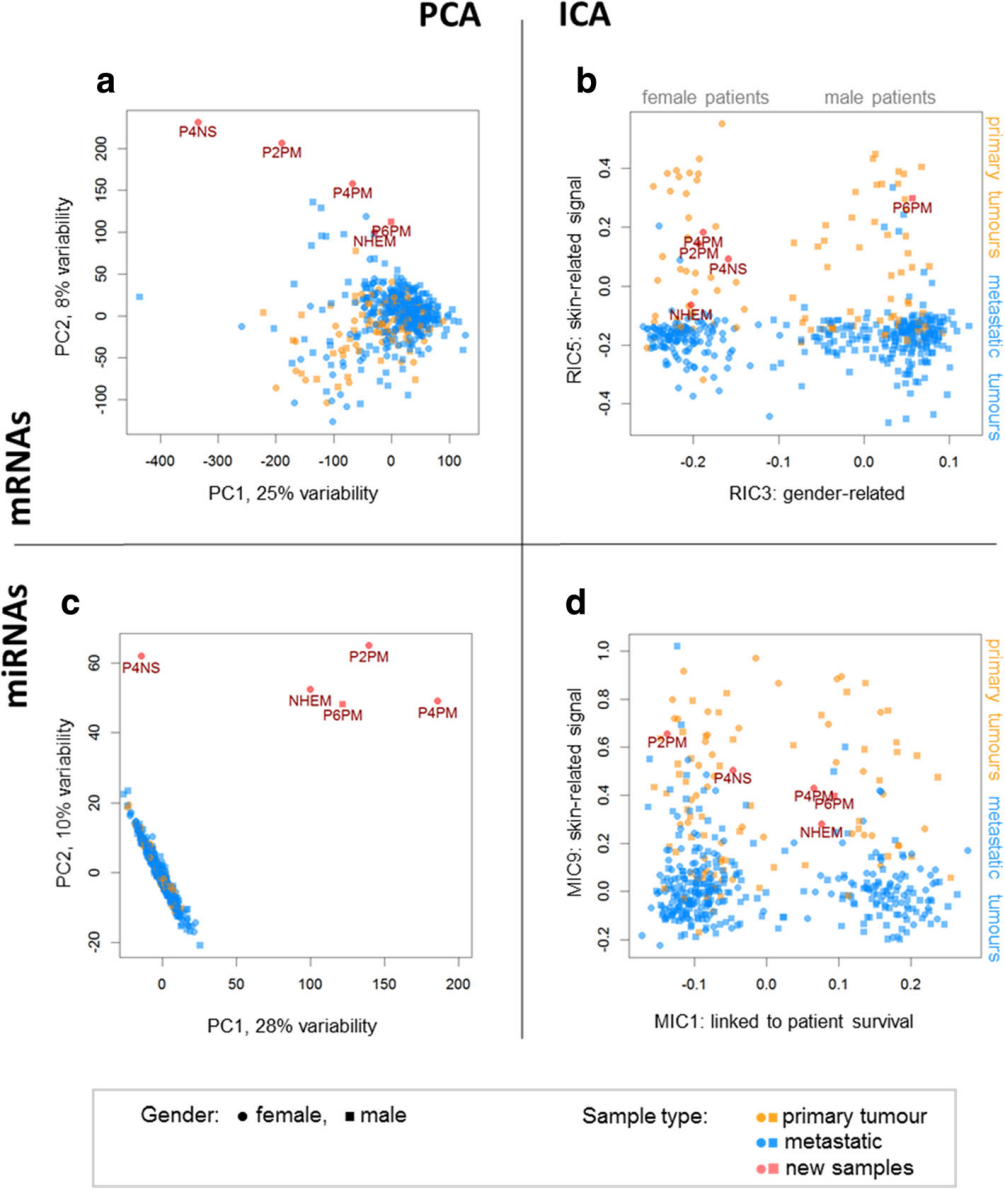
Data overview in the space defined by principal and independent components. Data variability captured by the first components of PCA (**a**) and two selected components of ICA (**b**) in gene expression data. Independent components were selected based on the predictive power of their weights for patient gender (RIC3) and sample type (RIC5). MiRNA data showed even higher discrepancy comparing miRNA-seq and qPCR results by PCA (**c**). However, in the space of independent components (MIC1 and MIC9), the samples studied by miRNA-seq and qPCR overlap (**d**)

The combined discovery/investigation dataset profiled by RNA-seq is presented in the space of two first principal components (Fig. 2a) and weights of two selected independent components (Fig. 2b). The two principal components included 33% of total variability and mainly reflected technical effects: PC1 was linked to the RNA-seq library size (data not shown) and PC2 segregates discovery and investigation data. Among all RICs, the components that reflected data clustering according to gender (RIC3) and sample type (primary or metastatic, RIC5) were chosen as an example. The investigation data were clearly integrated within the discovery data and showed reasonable clustering in Fig. 2b while preserving important clinical information (P6PM was the only male patient). Functional analysis showed that genes contributing to RIC5 participate in keratinocyte-specific functions and thus weights of RIC5 could be used as a marker of keratinocyte presence. Indeed, the vast majority of metastatic samples had low values of RIC5 weights, while primary tumours showed high values. NHEM (pure melanocytes) are devoid of keratinocytes and therefore clustered with metastatic tissues. We investigated whether other principal components can compete with independent components discriminating patient gender, sample type and tumour subtype. Results of ICA showed higher statistical significance than PCA in all comparisons (Additional file 3: Fig. S3A, C, E). In addition, AUC of ICA was higher for gender and sample type (Additional file 3: Fig. S3B, D) and only slightly lower for tumour subtypes (Additional file 3: Fig. S3F), where averaged AUC was reported. The observations were confirmed by Wilcoxon test (*p*-values are reported in Additional file 3: Fig. S3) and by 2-factor ANOVA on log-transformed *p*-values. Post-hoc analysis confirmed that ICA, on average, gives features that are linked stronger to clinical groups, than PCA (Tukey’s HSD *p*-value = 0.0175).

An even stronger correction effect of ICA was observed for miRNA data, for which discovery data were obtained by miRNA-seq and investigation – by whole miRNome qPCR arrays. PCA showed strong differences between log2 transformed counts and inverted Ct values (Fig. 2c). However, in the space of independent components, the investigation samples were properly located again (Fig. 2d). Here, two miRNA components MIC1 and MIC9 were depicted. MIC1 showed a strong relation to survival (Cox-based log rank *p*-value = 9.4e-4) while MIC9 was correlated with the skin-related signal of RIC5.

### ICA yields clinically relevant information

#### ICA as a feature-selection method for sample classification

As observed for patient gender and sample type in Fig. 2b, the weights of the components can be used as features with predictive potential. We investigated whether clinical factors could be predicted by weights originated from ICA deconvolution (only RICs considered). Three factors were selected: gender, sample type and RNA cluster, that could be considered as cancer subtype and was previously introduced in [18]. We validated the random forest classification directly on the discovery set using LOOCV, as described in the Methods section. In addition, nested cross-validation was performed excluding 20% of the data and estimating the optimal number of components. We obtained very similar accuracies (see Additional file 1) and estimated optimal numbers of components between 37 (lowest limit for tumour subtype) to 76 (upper limit for gender and sample type).

Next to cross-validation tests that were run directly on the discovery data, we applied ICA and random forest classification on two independent datasets joint with the discovery data: public validation (E-GEOD-19234, 44 samples) and in-house clinical investigation data (5 samples). Analysis was run independently for both cases, and the identified components were re-ordered and renamed to obtain comparable results. Notably, the metagenes of the new decompositions were reproducible and strongly correlated with the metagenes from ICA of single discovery set.

Predicting patient gender showed a high accuracy of 0.977 in the validation data, with only one samples misclassified. Testing sample type (primary/metastatic) for this validation cohort resulted in 34 samples classified as metastatic and 10 – as primary (accuracy of 0.773, as all validation samples were coming from metastatic tissue). However, as the precise excision location of the tumours is unknown, we cannot exclude that some metastatic tissues were collected from skin. Indeed, 7 of 10 misclassified samples showed high expression of keratinocyte marker genes *KRT5* and *KRT14*.

The investigation samples were classified as well and the results are presented in Table 1. Gender and sample types were accurately predicted for all the investigation samples but NHEM cells were considered “metastatic”, although with a border probability of 0.51: the best location predictors were weights linked to the transcriptional signal of keratinocytes, which was low in metastatic tumours and also in this primary cell line. Similarly, normal skin P4NS was classified as “primary” because classifier was not trained to distinguish melanoma from normal skin (absent in the training set).

**Table 1:** Performances of ICA-based feature extraction. Mean values of sensitivity and specificity are reported as well as class probability originated from random forest voting

Predicted variables	Groups	Accuracy (st.dev.)	Sensitivity specificity	P2PM (prob.)	P4PM (prob.)	P6PM (prob.)	P4NS (prob.)	NHEM (prob.)
Gender	female: 179	0.996 (< 0.001)	0.994 0.994	female (0.73)	female (0.66)	male (0.79)	female (0.68)	female (0.67)
	male: 293							
Sample type	primary: 105	0.871 (0.003)	0.733 0.733	primary (0.68)	primary (0.55)	primary (0.65)	primary (0.59)	meta-static (0.51)
	metastatic: 367							
Subtype (RNA cluster)	immune: 170	0.902 (0.006)	0.877 0.945	keratin (0.64)	keratin (0.48)	keratin (0.61)	keratin (0.64)	keratin (0.55)
	keratin: 102							
	MITF-low: 59							

**Fig. 3:**
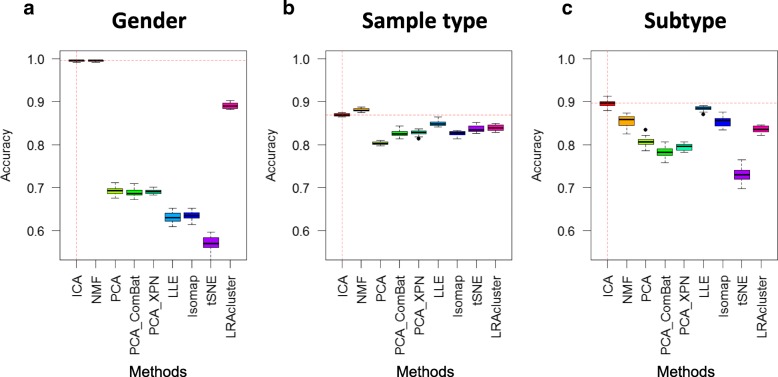
Benchmarking of ICA and other dimensionality reduction methods. Accuracies for classifying patients by gender (**a**), sample type (**b**) and tumour subtypes (**c**) were compared using 8 distinct methods. PCA was applied on the original data (PCA), as well as on the data corrected data using ComBat (PCA_ComBat) and XPN (PCA_XPN). The presented tools are described in the Methods section

We also compared the ability of ICA-based features to predict patient classes in comparison with other dimensionality reduction method (Fig. 3). The results indicate that ICA and NMF performed similarly well on classifying gender and sample type but ICA out-performed all other tools in terms of tumour subtype classification. Noteworthy, the reproducibility of NMF is very limited [34]. Overall, t-SNE showed the lowest accuracy of the 8 tested methods.

#### ICA provides prognostic features linked to patient survival

Next, prognostic abilities of the ICA weights were examined by a Cox regression model. All components, their significance and log-hazard ratios (LHRs) are summarised in Additional file 4: Tables S5 and S6. Eleven RICs and 3 MICs were found significantly linked to patient survival after multiple testing adjustment (adj.*p*-value by log rank test for Cox regression < 0.05). Among them, 6 RICs and 2 MICs showed very high stability of *R*^2^ > 0.8 and 8 out or 11 RICs were linked to biological processes. The remaining 3 RICs did not have enough contributing genes to run successful enrichment analyses. However, their behaviour over the samples allowed us to link two of them (RIC74 and RIC79) to the immune cluster, as is described in the next section. Although these 11 RICs and 3 MICs were statistically linked to survival in our discovery set, the predictive power of any of them was not sufficient to predict survival of new patients. Therefore, we combined the weights of these components into a risk score (RS) as described in Methods. Combined RS showed high significance (*p*-value = 2.2e-13) for the TCGA dataset.

**Figure 4:**
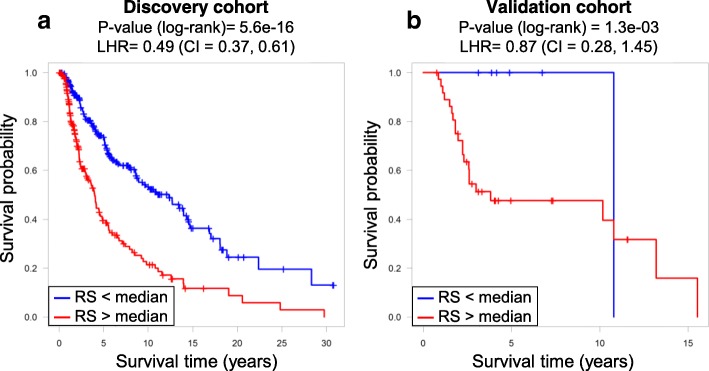
ICA-based risk score (RS) can predict patient survival. Performance of the risk score on the TCGA discovery patient cohort (**a**). Validation of the risk score on the independent cohort composed of 44 metastatic melanoma patients (**b**). Cox regression log hazard ratio (LHR) together with its 95% C.I. and log rank *p*-value are reported. In order to visualize the results as Kaplan-Meier curves, patients were divided into two groups by their RS (low risk – blue and high risk – red)

In order to validate the proposed risk scoring approach on an independent cohort of patients, we applied it on the validation dataset. The components that showed a significant link to survival (adj.*p*-value< 0.05) on the discovery set were then used to compose RS for the validation data and also showed significant prognostic properties (LHR = 0.87, *p*-value = 0.0013); Kaplan-Meier plots are shown in Fig. 4. The developed RS separated patients with low hazard (only one death among 7 patients, blue line in the validation cohort, Fig. 4b) from the group of patients with a high risk score.

For the three primary melanoma samples from the investigation set, the calculated RS was the highest for P6PM (RS = 1.92). This was in agreement with clinical observations, as patient P6 suffered from a very aggressive form of melanoma and deceased shortly after sample collection. From the quantitative results obtained from the validation dataset and qualitative differences observed for the investigation dataset, we concluded that weights of independent components can be combined into a risk score, suitable to predict patient survival.

### Independent components provide information about biological processes in tumours

#### General strategy

**Fig. 5:**
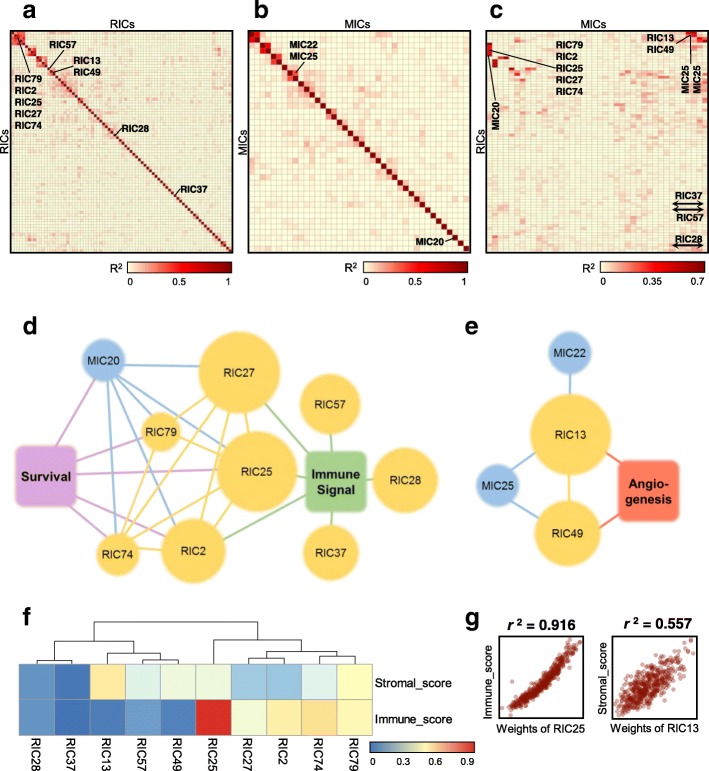
Correlated component clusters. Heatmaps showing coefficient of determination (*r*^2^) between weights of RIC-RIC (**a**), MIC-MIC (**b**) and RIC-MIC(**c**). The cluster of components (**d**) is based on gene components (RICs) linked to immune response via enrichment analysis of top-contributing genes; cluster (**e**) is based on RICs linked to angiogenesis and stroma transcriptional signal. The size of the circles illustrates the number of top-contributing genes and miRNAs in the components. RIC and MIC components have been linked to each other on basis of correlation (edges between components show *r*^2^ > 0.25). As an additional validation, the weights of the described components were compared with ESTIMATE [9] scores and corresponding *r* ^2^ are shown in (**f**). The weights of the RIC25 and RIC13 components correlated best to immune and stromal scores, shown in (**g**)

The most challenging part of ICA is assigning components to specific biological processes, cell types and technical factors. The approach we have taken is outlined in Additional file 3: Fig. S1 (green panel) and the Methods section. The automatically generated reports describing the components can be found in the Additional file 2: Supplementary Results. We also linked RICs and MICs based on squared Pearson correlation (or coefficient of determination, *r*^2^) between weights of corresponding components. Correlation maps are presented in Fig. 5a-c and two clusters of the components in Fig. 5d-e. Finally, we compared our findings to previously published immune and stromal scores calculated by the well-accepted ESTIMATE algorithm [9] (Fig. 5f-g).

#### Immune components

The biggest cluster of RICs was linked to immune cells and immune response. Based on functional annotation it included seven components: RIC2, RIC25, RIC27, RIC28, RIC37, RIC57 and MIC20. RIC2, RIC25 and RIC27 showed correlated weight profiles between themselves and with RIC74, RIC79 and MIC20 (Fig. 5d and Additional file 2: Supplementary Results). Immune component RIC2 was strongly linked to survival (LHR = -0.89, *p*-value = 1.8e-4) and most probably originated from B cells (Enrichr “B cells” category enriched, adj.*p*-value = 3.9e-6). The metagenes of RIC2 were also correlated with the LM22 signatures for B cells (Additional file 3: Fig. S5B, and showed the highest correlation with B cell profiles measured in single cells, Additional file 3: Fig. S6). Interestingly, RIC25 almost perfectly reconstructed the ESTIMATE immune score (Fig. 5 f-g). RIC27 showed a very similar collection of enriched gene sets, but was much less correlated to this particular score, suggesting that ICA shows better sensitivity and captures more cell subtypes than ESTIMATE.

Functionally, RIC28 was linked to inflammatory responses to wounding (adj.*p*-value = 6.3e-22), neutrophil degranulation (adj.*p*-value = 1.3e-7), *TNF*- (adj.*p*-value = 4.7e-8) and *IL1*-mediated signalling pathways (adj.*p*-value = 2.2e-9); RIC37 was connected to interferon signalling (adj.*p*-value = 5.1e-22) whose metagenes were also reciprocally correlated with M5_INTERFERON of the Biton dataset [10] (Additional file 3: Fig. S5A). Neither RIC28 nor RIC37 were detected by ESTIMATE scoring.

Components RIC74 and RIC79 contained a very limited number of top-contributing genes, but both were significantly linked to survival (*p*-values of 1.3e-3 and 3.2e-3). No specific cell type was associated with these components. RIC74 was, however, associated with positive and negative regulation of immune response and receptor-mediated endocytosis (all adj.*p*-values = 2.6e-4).

The weights of miRNA component MIC20 were positively correlated with the weights of RIC2, RIC25 and RIC27 (correlation of 0.69, 0.86 and 0.64 accordingly) and were positively linked with survival (LHR = − 1.32, *p*-value = 1.2e-4). Among the top miRNAs in MIC20 were *miR-155*, *miR-150*, *miR-342*, *miR-146b*, and *miR-142*. MiR-155 is known to be a regulator of immune response in cancer cells [37, 38] while *miR-150*, *miR-155* and *miR-342* have been proposed as markers for melanoma patient survival [39]. Interestingly, four of those positively contributing miRNAs formed a cluster on chr1q32.2 (adj.*p*-value = 7.3e-3).

**Fig. 6:**
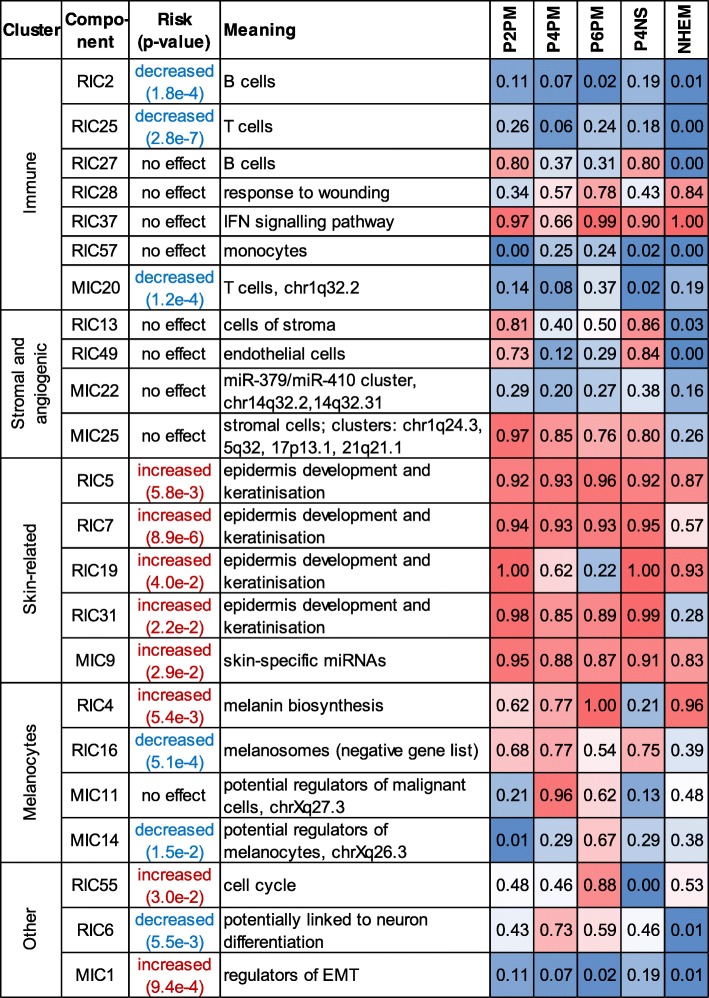
Biologically relevant components and their ranked weights in the investigation dataset. Rank for samples is calculated in comparison to the TCGA discovery set (red – weight above median in TCGA samples, blue – below)

The samples from the investigation cohort were characterised by the involvement of the above immune response-related components (Fig. 5d). The results are presented in Fig. 6. All components linked to subpopulations of immune cells (RIC2, RIC25, RIC57, MIC20) showed little involvement in the patients of investigation cohort suggesting low overall immune reactions to the tumour except specific interferon responses, which had high weights in the investigation samples (RIC28, RIC37). Similarly, we checked behaviour of these components for the validation dataset (Additional file 3: Fig. S7). RIC2 and RIC25 showed tendency to predict better survival (LHR < 0) and their weights are higher for censored patients than for dead. However the *p*-values from Cox regression on 44 validation samples were not conclusive (RIC2: 0.154, RIC25: 0.06).

#### Stromal and angiogenic components

The second cluster of RICs was linked to the signals of stromal cells and showed enrichment in genes related to angiogenesis. It included four correlated components: RIC13, RIC49, MIC22 and MIC25 (Fig. 5e, Additional file 2: Supplementary Results). Genes of component RIC13 were enriched in extracellular matrix organisation (adj.*p*-value 2e-26) and vasculature development (adj.*p*-value = 5e-23). The component’s metagenes were strongly correlated with metagene M3_SMOOTH_MUSCLE of Biton et al. [10]. In the single cell study, the highest correlation of RIC13 metagenes was observed with cancer-associated fibroblasts. Most probably, this component is linked to cells of tumour stroma, which again is supported by high correlation with the ESTIMATE stromal score (Fig. 5f-g). Another component from this cluster, RIC49, showed enrichment in GO-terms linked to blood-vessel development and angiogenesis (both with adj.*p*-value = 6e-24). Its most correlated single cell type was endothelial cells, which also form part of the tumour microenvironment. Thirteen of the positively contributing miRNAs from MIC22 were strongly concentrated in a narrow genomic region in chr14q32.2 (adj.*p*-value 5.8e-11). MiRNAs of MIC25 were significantly enriched in four cytogenetic locations: chr1q24.3, chr5q32, chr17p13.1 and chr21q21.1 (adj.*p*-values of 5.0e-6, 2.6e-3, 4.1e-02 and 9.7e-5, respectively).

In the clinical samples of investigation cohort, the highest amount of stromal and endothelial cells was observed in P2PM and P4NS samples (Fig. 6). The primary cell line NHEM showed almost no signal of stromal cells. Interestingly, MIC25 was heavily weighted in all new patient samples, excluding this cell line.

#### Skin-related components

RIC5, RIC7, RIC19, RIC31 all showed an enrichment in GO terms related to processes of the skin including epidermis development (adj.*p*-value<2e-15 for all mentioned components) and keratinisation (adj.*p*-value< 1.4e-10). Enrichr suggested that the signals of these components are specific to skin (adj.*p*-value<1e-50). The dataset contained 48 keratins and many of them were observed among the top-contributing genes: 20 for RIC5, 28 (RIC7), 30 (RIC19) and 13 (RIC31). RIC5 and RIC7 were negatively correlated with survival, which is in concordance with previous observations [18]. MIC9 with the skin-specific *miR-203* [40], was linked to RIC5, RIC7 and RIC31. Furthermore, several components (RIC4, RIC16, MIC11 and MIC14) were connected to the activity of melanocytes. Top-contributing genes of RIC4 were enriched in the melanin biosynthesis process (adj.*p*-value = 1.2e-5) and Enrichr linked these genes to melanocytes (adj.*p*-value = 2.8e-25). RIC16 showed an inverse correlation of the weights with RIC4. Both components were linked to survival, but with an opposite effect: while RIC4 increased the risk (LHR = 0.18, *p*-value = 5.4e-3), RIC16 increased the survival (LHR = -0.23, *p*-value = 5.1e-4) (Additional file 2: Supplementary Results). Many positively contributing miRNAs of the MIC11 component (16 of 33) – a miRNA cluster associated with early relapse in ovarian cancer patients [41] – were located on chrXq27.3 (adj.*p*-value<1e-7).

In the validation cohort we identified several patients with a strong skin signature (Additional file 3: Fig. S7, skin-related cluster). These samples also showed a high expression of keratinocyte markers, such as *KRT5* and *KRT14* and most probably originated from skin metastasis. Interestingly, component RIC16 was not strongly presented in the validation dataset, which could suggest absence of healthy melanocytes in the metastatic samples. Contrary, RIC4 was strongly presented and linked to increased risk in the validation cohort (*p*-value = 5.3e-3).

#### Other tumour-related components

Some components could be linked to transcriptional signals and regulation of cancer cells. For example, RIC55 captured the cell cycle process (adj.*p*-value = 6.6e-29) and the majority of the 383 genes positively associated to this component are known to be involved in cell cycle control with tumour cells contributing the most to cell division activities. Increased cell proliferation was linked to survival (*p*-value = 3.0e-2). In the investigated samples, the highest weight was observed for the most aggressive tumour P6PM and the lowest value for normal skin P4NS. In the validation samples this component was also linked to survival (*p*-value = 3.5e-3).

Several RICs showed linkage to neural tissue. As an example, both positive and negative top-contributing genes of RIC6 were linked to brain in the ARCHS4 tissue sets of Enrichr (both adj.*p*-values <1e-33). This component was as well associated with patient survival (*p*-value = 5.5e-3). The component indicates the ability of melanoma cells to show expression patterns specific for cells of the neural crest of human embryos and can be linked to motility of malignant melanocytes.

MiRNA component MIC1 showed an interesting bi-modal distribution in the discovery dataset (see two clusters in Fig. 2d) and was strongly linked to patient survival (Cox *p*-value = 9.4e-4), suggesting two subgroups of melanoma patients with different prognosis. This component most probably was linked to regulation of epithelial-mesenchymal transition (EMT), as many miRNA positively or negatively influencing the component are known to be EMT regulators or linked to metastasis formation: *miR-551*, *miR-206*, *miR-34a*, *miR-1269*, *miR-205*, *miR-876*, *miR-301b*, and *miR-365a*. Based on our analysis of the discovery TCGA dataset, these miRNA listed in Additional file 2 can be further investigated as potential survival markers for melanoma patients.

### ICA-derived biological networks

Given the promising results with regard to immune- and angiogenesis-related components, we performed text mining (described in Additional file 1: Supplementary Methods) on the terms “B-cell, miRNA and/or cluster”, “T-cell, miRNA and/or cluster” and “angiogenesis, miRNA and/or cluster”, and compiled a list of published miRNAs involved in immune responses and angiogenesis. For the shared top-contributing miRNAs from MIC20, 22, and 25 (Fig. 5 and Additional file 2: Supplementary Results), experimentally confirmed target genes were extracted (from miRTarBase [42]). In order to investigate possible miRNA-target gene interactions as an underlying biological reasoning for clustering, we next overlaid the extracted target genes with gene lists of connected RICs. Enrichment analysis was performed and final gene lists were analysed by STRING [43] to visualise potential protein-protein interactions for target genes of immune component cluster (Additional file 3: Fig. S8) and angiogenic component cluster (Additional file 3: Fig. S9). Overall, the networks showed a significant enrichment of interactions suggesting a non-random relation between top-contributing miRNAs and genes. STRING network analysis captured key biological interactions reflecting the ICA-based RICs and MICs, from which they were initially derived.

## Discussion

Here we investigated the applicability of ICA-based deconvolution of transcriptomes, originated from a large set of bulk melanoma samples, for acquiring clinically and biologically relevant information about new patients. ICA decomposes transcriptomic data into components that are characterised by two matrices: a matrix of metagenes, which shows how each gene contributes to each component, and the matrix of weights that represents the involvement of the components in each sample. Importantly, this analysis does not require any preliminary knowledge about biology or sample composition. Unlike other deconvolution methods that use signatures [9] or pure transcriptomic profiles [8], ICA is an assumption-free, unsupervised approach. The method directly works with the data from bulk samples without any preliminary assumption about the transcriptomes of the purified cell types. Among the components, one can expect to see not only those defined by “pure” tumours or stromal cells, but also those originating from tumour/stroma interactions including tumour-induced stromal cell reprogramming. One example of such interactions is angiogenesis, further discussed below.

We implemented a robust consensus ICA method and applied it to several datasets from patients with SKCM. These included (a) a large cohort of SKCM patients from TCGA used as discovery set; (b) an independent cohort of 44 patients with publicly available microarray mRNA data and (c) 5 in-house clinical investigation samples: 3 primary melanomas, a normal skin sample and a normal melanocyte cell line (NHEM). Both mRNA and miRNA datasets were obtained for the discovery and investigation samples. Despite the fact that different techniques were used for data acquisition, ICA was able to identify common signals in the datasets and properly allocate the new samples within the discovery set (Fig. 1). This was particularly evident for miRNA data where the discovery set was obtained by small RNA-seq and the new samples by qPCR arrays with PCA showing a strong difference between these two datasets. With ICA, technical biases in the data were isolated within several components and thus separated from biologically relevant signals leading to a better and more correct characterisation of the samples. Such batch correction, of course, could also be performed by other methods. We tested several correction methods together with standard dimensionality reduction methods and showed that overall, ICA performed best across them. We recently applied ICA-based batch correction on single-cell RNA-seq data and confirmed its usefulness [44].

The fact that ICA should be re-run for every series of new samples could be considered as a drawback of our approach. However, similarly to PCA, recalculation of the components does not require supervision and could be done automatically. In the case when investigation and discovery datasets come from the same distribution, one can use the matrix ***S*** obtained from the discovery dataset in order to define the weights (***M***) for the samples forming the investigation dataset (1). However, in reality, the variability in the data requires recalculation of the components for the new investigated samples.

We demonstrate here that the weights of independent components can be used as predictive features of patient subgroups and can be linked to patient survival. We also propose a method to select the number of components, based on the required classification task (Additional file 2 and Additional file 3: Fig. S10). While the ICA-based feature extraction method has been previously discussed (e.g. [12, 45]), no studies have been devoted, to our knowledge, to estimating patient prognosis using ICA-based data deconvolution. We combined weights of several significant components into a risk score, for which a high predictive power was shown both in the discovery cohort (460 patients with known survival status) and in the independent validation cohort (44 patients). Thus, the developed approach could help clinicians in estimating the risks and potentially optimising the selection of adequate treatment strategies. Three of the survival-associated components were connected to immune response. As expected, higher immune signal indicated lower risk for the patients [21]. Interestingly, all 4 skin-related mRNA components were also linked to survival but inversely, which is in agreement with previous observations of poor survival for patients of keratin subtype [18].

Next, the biological relevance of the components was examined in depth. We showed that only one subset of genes, either positively or negatively contributing, is strongly associated to biological functions (Additional file 3: Fig. S11). Components that represented signals from various cell subpopulations (e.g. different immune cells, stromal cells, melanocytes) and cellular processes (e.g. cell cycle) were identified. These signals were also detected in the new samples, providing hints of active processes and tissue composition of these samples. We associated mRNA and miRNA components that showed similar weight profiles in all the patients and hypothesised that such components were probably derived from the same cell types or process. This hypothesis was supported by our observations. Indeed, MIC20 was correlated with RIC2 and RIC25 – the components associated with leukocyte activity. Indeed, *miR-155*, one of the markers of immune cells [46], was found among the most contributing miRNAs of MIC20. Therefore, we could link all other top-contributing miRNAs within MIC20 to leukocytes and immune response and thus assign functions to these miRNAs.

Another group of components were linked to tumour-stromal interactions and angiogenesis. One of them, MIC22, contained an almost complete miRNA mega cluster, *miR-379/miR-410*, with 11 of 13 miRNAs significantly contributing. The cluster is located on chromosome 14 (14q32) in the so-called imprinted *DLK1-DIO3* region. Lower levels of this miRNA cluster have been described to favour neo-vascularisation [47] and shown to play a role in development, neonatal metabolic adaption but also in tumorigenesis. Deregulation of miRNAs in this locus has recently been shown to predict lung cancer patient outcome [48, 49]. Most miRNAs in this cluster (68%) were significantly downregulated in glioblastoma multiform, 61% downregulated in kidney renal clear cell carcinoma and 46% in breast invasive carcinoma indicating a tumour suppressive role of this miRNA cluster, especially in glioblastomas [50]. Moreover, it was shown that the *miR-379/miR-410* cluster was silenced in melanoma, which favoured tumorigenesis and metastasis [51].

Overall, we observed that ICA on miRNA expression data grouped together many miRNAs that belong to genetic clusters and by connecting MICs with genes (RICs), biological functions of miRNAs could be inferred. As an example, MIC11 represents a cluster on chrX q27.3 associated with early relapse in advanced stage ovarian cancer patients [41]. In our analysis, the miRNAs from this cluster were linked to activity of malignant melanocytes. All this is suggestive of a concerted role for miRNAs of a given cluster in regulating functionally related genes [52, 53].

The results for the ICA-derived biological networks implied that the combination of ICA with text mining (biological expressions enriched in statistically correlated RICs and MICs) potentially uncovers two hidden connections: biological reasons for statistical correlations and detection of those genes actually responsible for the biological link between MICs and RICs. This in turn might give new insights into the significance of biological processes active in cancer in general or in certain cancer subtypes.

Similarly to PCA or NMF, ICA could be integrated into standard analysis pipelines in the future. Unlike PCA, ICA could extract biologically-based signals. These signals are more stable than those obtained by NMF and can be further used to acquire clinically relevant information about new samples, thus helping patient diagnostics and prognostics.

## Conclusions

In conclusion, we used the consensus ICA method to combine transcriptomics data of melanoma patients with large public datasets. Here we showed successfully that the ICA-based decomposition separates true biologically relevant transcriptional signals from technical biases. The obtained ICA-based features were used to predict cancer subtypes and patient survival. We also showed how cellular composition and biological signals can be uncovered within new clinical samples. Transcriptional signals from immune cells, melanocytes, keratinocytes and stromal cells were identified and confirmed by comparison to published signatures. We demonstrated that some of the identified signals, including immune activity and cell proliferation, are linked to the aggressiveness of tumours and could influence patient survival. Finally, we were able to integrate miRNA and mRNA data, which allowed us to deduce biological functions of miRNAs.

## Supplementary information

**Additional file 1:** Supplementary Methods detailed description of data acquisition and independent component analysis. (DOCX 45 kb)

**Additional file 2:.** Supplementary Results automatic report generated for the discussed components by the presented consensus ICA tool (consICA). (PDF 1107 kb)

**Additional file 3: Fig. S1.** Flow chart of post-processing of ICA results to determine biological relevance of components and networks. **Fig. S2.** Schematic workflow of ICA application to the discovery, validation and investigation datasets. **Fig. S3.** Performance of principal component and independent components as feature selection methods. **Fig. S4.** Similarity between the identified components improves with the increase of number of runs for consensus ICA. **Fig. S5.** Correlating components to profiles defined in the previous studies. **Fig. S6.** Pearson correlations between the metagenes of three immune components RIC2, RIC25, RIC57 and one angiogenic component RIC49 on one side, and averaged expression profiles of single cell sub-populations published by Tirosh et al. **Fig. S7.** Behavior of the important RICs in the validation dataset (same order and RIC naming as in Fig. 6). **Fig. S8.** STRING networks based on overlapping MIC20-meta-target genes and RIC2 (A), RIC25 (B), RIC27 (C), RIC74 (D) metagenes, showing a significant protein interaction network (medium confidence:0.400, PPI enrichment *p*-values: < 1.0e-16, < 1.0e-16, < 1.0e-16, and 9.77e-06 accordingly) representing main players within immune response. **Fig. S9.** STRING networks based on overlapping: MIC22-target genes and RIC13 metagenes (A), MIC25-target genes and RIC13 metagenes (B), MIC25-target genes and RIC49 metagenes (C). **Fig. S10.** RNA-seq data preparation and metric selection based on discovery TCGA SKCM dataset. **Fig. S11.** (A) Number of significant positively (red) and negatively (blue) contributing genes in metagene of each of them RNA components before re-orientation. (PDF 2978 kb)

**Additional file 4: Table S1.** Survival data of TCGA SKCM covering patient ID, age at diagnosis, year of diagnosis, disease status at last contact, days to death, vital status, days to last contact after initial diagnosisdiagnosis, year of last contact, tumour free survival, survival in years and age at death. Data items have been either directly extracted from individual XML-files holding clinical information publicly available at GDC or calculated based on the extracted information. **Table S2.** Clinical data of TCGA SKCM covering patient ID, gender, sample type (primary tumour and metastatic) and publication based data for RNA-seq cluster (immune / keratin / MITF-low). **Table S3.** Parameters of clinical samples and controls in the investigation dataset. **Table S4.** Ct values obtained for the new samples using qPCR arrays. During analysis, all absent (NA) values and Ct > 36 were replaced by 36, value selected as detection limit. **Table S5.** Summary of the ICA results for mRNA data. Stability of each component after 1000 runs, results of the survival analysis, number of genes significantly involved (adj.*p*-value< 0.01) and number of enriched GO biological processes (adj.*p*-value< 0.01) are reported. Lower and higher estimates of log hazard ratio (LHR) correspond to 95% C.I. **Table S6.** Summary of the ICA results for miRNA data. Stability of each component after 1000 runs, results of the survival analysis and number of significantly involved miRNAs (adj.*p*-value< 0.01) are reported. (XLSX 122 kb)

## Data Availability

The developed tools are available at https://gitlab.com/biomodlih/consica. RNA-seq data for the samples used to build the investigation dataset are available by GEO accession number GSE116111 (https://www.ncbi.nlm.nih.gov/geo/). Ct-values for all quantified miRNAs are available in Additional file 4: Table S4. The results of ICA and their biological interpretation are presented in Additional file 2. The validation gene expression dataset used in this study is available from ArrayExpress under the accession number E-GEOD-19234.

## Abbreviations

ANOVA: Analysis of variance
EMT: Epithelial-mesenchymal transition
FPKM: Fragments per kilobase million
GEO: Gene Expression Omnibus
ICA: Independent component analysis
LHR: Log-hazard ratio
LOOCV: Leave-one-out cross-validation
MIC: microRNA independent component
miRNA: micro-RNA
NMF: Non-negative matrix factorization
PCA: Principal component analysis
qPCR: quantitative polymerase chain reaction
RIC: mRNA independent component
RNA-seq: RNA-sequencing
RS: Risk score
SKCM: Skin cutaneous melanoma
TCGA: The Cancer Genome Atlas
TPM: Transcripts per kilobase million
Tukey’s HSD: Tukey’s honest significant difference test
