# Supplementary material for: Deconvolution of transcriptomes and miRNomes by independent component analysis provides insights into biological processes and clinical outcomes of melanoma patients
Source: BMC Med Genomics. 2019 Sep 18;12:132. doi: 10.1186/s12920-019-0578-4 (PMC6751789; doi:10.1186/s12920-019-0578-4)
Supplement: Supplementary file 1 — Additional file 1: Supplementary Methods detailed description of data acquisition and independent component analysis. (DOCX 45 kb) [file 12920_2019_578_MOESM1_ESM.docx]

## SUPPLEMENTARY METHODS

### Detailed description of discovery and validation datasets preparation

Expression data. As a discovery dataset, we used the open-access TCGA skin cutaneous melanoma (SKCM) datasets, namely RNA-seq (HTSeq raw counts, FPKM and TPM) and miRNA-seq data (miRNA isoform read count) from the Genomic Data Commons (GDC) data portal of the National Cancer Institute of the National Institutes of Health (NIH, https://portal.gdc.cancer.gov/). The RNA-seq dataset comprises data from 468 different individuals (472 samples). Of those, 368 originated from metastatic samples (1 individual provided 2 samples) and 103 from primary melanoma tumours; one sample represented a solid normal tissue. MiRNA-seq data were available for 452 individuals, with 353 metastatic, 97 primary tumour and 2 normal skin tissue samples. The miRNA-isoform read counts data were collapsed per isoform and IDs were mapped to miRNA-names based on miRBase v. 21 (http://www.mirbase.org/).

A validation dataset of gene expression data was taken from Bogunovic et al. [1], available from ArrayExpress under E-GEOD-19234. This Affymetrix GeneChip Human Genome U133 Plus 2.0 microarray dataset consisted of 44 metastatic samples from melanoma patients accompanied by survival information. As microarray expression data have very different dynamic range compared to RNA-seq [2], we shifted and scaled the microarray data: the 5th percentile of expression was used as the lowest meaningful signal and was subtracted from microarray gene expression. All negative values were set to 0. The data were then scaled to unify the 75th percentile between discovery RNA-seq and validation microarray data.

Clinical data. To explore the possibility of assigning clinical traits to TCGA samples, we compiled a small dataset based on public TCGA data with “fail-safe” items covering gender and sample type (primary tumour and metastatic). Additionally we added publication-based data for RNA-seq clustering (immune / keratin / MITF-low) [3] as this information has been claimed to be relevant for disease prognosis. Survival data were extracted by parsing related information out of publicly available individual clinical data files (XML files provided by GDC). We extracted and processed the information assigned to the tags: bcr_patient_barcode, days_to_death, vital_status, year_of_initial_pathologic_diagnosis, age_at_initial_pathologic_diagnosis, days_to_last_followup and person_neoplasm_cancer_status. The full survival and clinical datasets are described in Additional file 4, Tables S1 and S2, respectively.

### Preparation of the investigation dataset: clinical samples, data acquisition and analysis

Melanoma biopsies of three Caucasian patients were collected after surgical resection at the Dermatology Department of the University Clinic Freiburg, Germany. All patients signed an informed written consent. Ethical approval of this study was obtained from the Comité National d’Ethique de Recherche Luxembourg (CNER-No. 201201/05) and from the German Ethik-Kommission der Albert-Ludwigs-Universität Freiburg (EK-Freiburg 196/09). Histological examination and estimation of the percentage of tumour cells within the lesion was performed by two independent pathologists (normal skin and NHEM cell line were free of tumour cells). Tissues from snap frozen biopsies were lysed in RLT buffer with a Qiagen TissueLyser (50 Hz, 5 min). DNA and total RNA were extracted using the Qiagen’s AllPrep Mini Kit according to supplied protocols. Quality and quantity of samples were measured with Nanodrop, gel electrophoresis and Qubit High Sensitivity Kit. RNA integrity was determined using the Agilent Bioanalyzer Nano chip.

The investigation dataset, represented by RNA-seq and miRNA qPCR array data, is composed of primary tumour samples of three melanoma patients and two control samples (one matched normal skin and a healthy melanocyte cell line, NHEM). Sample annotation is presented in Additional file 4, Table S3. Details of sample collection, preparation, transcriptome and miRNome analyses are described in Supplementary Methods. RNA-seq data for these samples are available by GEO accession number GSE116111 and Ct-values for all quantified miRNAs are available in Additional file 4, Table S4.

To harmonise miRNA annotation of qPCR arrays and TCGA-derived miRNA isoform read count data, we first re-annotated our qPCR arrays to miRNA version 21. To have comparable data between qPCR arrays and TCGA, we worked with miRNA isoform data referring to miRNA IDs, so that mapping of stem loop IDs to mature miRNA IDs was possible.

### Transcriptome analysis of clinical samples (test dataset)

RNA samples were sequenced on the Illumina sequencing platform. Paired-end transcriptome sequencing including library preparation was done at the Translational Genomics Research Institute (TGen), Arizona (USA). Sample preparation was performed using Illumina’s TruSeq Sample Prep Kit, amplification was done on Illumina’s cBot Cluster Generation System. PE reads (2x 83 bp) were generated on the Illumina HiSeq 2000.

Concerning the RNA-seq analysis of clinical samples, we followed the GDC bioinformatics pipeline with minor modifications, starting from quality-controlled fastq-files (https://docs.gdc.cancer.gov/Data/Bioinformatics_Pipelines/Expression_mRNA_Pipeline/). We used the GDC provided reference files (https://gdc.cancer.gov/about-data/data-harmonization-and-generation/gdc-reference-files/) for *STAR* alignment (star.index.genome.d1.vd1.gtfv22.tar.gz) and annotation (gencode.v22.annotation.gtf.gz).

The following steps were performed: i) initial *FastQC* run with visual inspection of reports , ii) applying *Trim Galore!* with main focus on adapter trimming (options: --fastqc -q 10 --length 35 --paired --retain_unpaired; unpaired reads have been discarded from further analysis) and less directed to quality trimming, iii) alignment of fastq-files 1st pass with *STAR* (v2.5.2b), iv) intermediate index generation with *STAR*, v) alignment 2nd pass, vi) counting reads of mapped counts has been achieved directly during the 2^nd^ alignment pass of STAR by applying the option *– quantMode TTTranscriptomeSAM GeneCounts.* We normalized expression data by the FPKM method provided by GDC (https://docs.gdc.cancer.gov/Data/Bioinformatics_Pipelines/Expression_mRNA_Pipeline/#fpkm), wherein gene length data used to process TCGA sample have been kindly provided by GDC on demand.

### MiRNome analysis of clinical samples

MiRNAs of clinical samples (Additional file 4, Table S3) were profiled by qPCR arrays. The clinical tumour samples as well as a NHEM control were profiled in duplicate using whole miRNome miScript miRNA qPCR arrays (Qiagen, v.16, 1066 miRNAs; based on miRBase v16). All kits, reagents and qPCR arrays were used according to the manufacturer’s instructions. Following RNA isolation, 5μl of the eluted total RNA was reverse transcribed in a 10μl reaction volume with the miScript II RT kit (Qiagen) using Hispec buffer. The 1:5 diluted cDNA was pre-amplified with the miScript PreAMP PCR kit (Qiagen) using the corresponding primer mixes (whole miRNome primer mix for whole miRNome qPCR arrays and custom primer mix for custom qPCR arrays). Pre-amplification control experiments were performed by RT-qPCR using primer assays for miR-16-5p, SNORD95, cel-39 and miRTC (internal miRNA reverse transcription control). Quality controlled pre-amplified cDNA was diluted 1:5 and further used for miScript whole miRNome and custom qPCR arrays (Qiagen). Real-time PCR detection on the qPCR arrays was carried out on a CFX384 Detection System (Bio-Rad).

For qPCR array data analysis, baselines and thresholds were adjusted as recommended by the supplier and Ct values were exported for analysis. Threshold cycle (Ct) values greater than 36, as well as primers with bad quality melting curves were assigned as 36 – such Ct value was considered as a zero level. In order to bring log counts of TCGA and experimental Ct values to consistent scales, we subtracted measured Ct values from 36; thus, low expression of miRNA corresponds to low values of 36 – Ct.

### Selection of optimal metrics for ICA of RNA-seq data and estimating the number of components

We started our analysis by selecting the optimal gene expression metrics suitable for ICA and considered thus four standard options: raw counts, library-size-normalized counts (DESeq2) [4], and two gene-length and library-size normalized measures: FPKM and TPM. ICA was performed on pre-filtered and log-transformed gene expression data, as described in the Methods section (discovery TCGA SKCM dataset, Additional file 3, Figures S10A,B); then, predictive abilities of the weight matrix resulting from consensus ICA was investigated. Interestingly, raw counts outperformed the three other metrics in terms of AUC when predicting our benchmark traits gender of the patients (Additional file 3, Figure S10C) and sample type: *primary* or *metastatic* (Additional file 3, Figure S10D). Library-size normalized counts showed similar results to raw counts separating *male* and *female* patients, but had a lower AUC for the sample type. ICA of FPKM and TPM always required more components to capture the difference between patient groups. ICA with only 20 components was able to capture *patient gender* assigning one of the components to it and allowing prediction with AUC>0.95, while TPM and FPKM required 40 and more components to perform similarly. *Tissue type* was well identified even with a small number of components. However, for the raw counts the AUC was better when using 52 or more components. Most probably, normalisation changes data structure and complicates signal decomposition by ICA. It should be noted, however, that despite the negative effect of normalization on this analysis, we used log-transformed data, as ICA did not perform well on highly skewed data [5]. Therefore, we used raw count values in log scale as an input to ICA-based deconvolution and patient classification.

There is no single scheme to define the exact number of components to be used, as it is task specific. One approach was recently published by Kairov et al [6], but it was aimed at maximizing the stability of the components and required several datasets to be used. The authors suggested that a higher number of components should not harm the analysis. We also only observed improvement of predictive power with growing number of components. In order to account for technical differences between sequencing platforms and not lose important biological information, we selected 80 components for RNA-seq data. The MiRNA dataset only had a limited number of features – 911, so we used a smaller number of components – only 40.

In order to check whether our selection was reasonable, we performed a series of nested cross-validations, excluding 20% of the discovery dataset from consideration, estimate the optimal number of components and train the random forest classifier on the remaining 80%. Then, we tested the classifier on the excluded 20%. We observed good and reproducible results. The averaged values are shown below with 95% confidence intervals.

- **Gender**: optimal number of components = 68 ± 8, accuracy = 0.976 ± 0.022
- **Sample type**: optimal number of components = 67 ± 9, accuracy = 0.873 ± 0.004
- **Subtype**: optimal number of components = 42 ± 4, accuracy = 0.889 ± 0.019

### Only one side of a component matters: redirecting the components

An interesting imbalance of the components was observed during the over-representation analysis of influential genes. Significance analysis resulted in approximately the same number of positively and negatively contributing genes (Additional file 3, Figure S11A). However, after functional annotation, only one group of genes, either positive or negative, showed the highest number of significant GO terms and lower adj.p-value (Additional file 3, Figure S11B). Thus, biologically-relevant independent components are linked to biological processes either by positively or by negatively contributing gene lists, but not both. In multiple runs, ICA assigns the gene involvement in the way that the product of ***S*** matrix column and ***M*** matrix row has a certain constant sign, whereas signs of ***S*** columns and ***M*** rows may change. To avoid this uncertainty, we oriented RIC in the way that positive influential genes correspond to the most statistically significant GO terms. An exception to this rule was observed for the component linked to gender: the negative list of genes showed a strong preference for the X chromosome (with XIST being the most significant member), while the positive list of genes came mainly from the Y chromosome (such as *KDM5D* and *DDX3Y*).

### miRNA targets form edge-enriched gene networks

Based on intermediate results suggesting a connection of network-related RICs to specific cell types, namely T- and B-cells or angiogenesis, we used these expressions as keywords, assuming a biological link between MICs and RICs. After automatically extracting miRNA-names and clusters from publication titles and abstracts by an inhouse Python script (available on demand), we compared those with the miRNA-metagenes comprised in the correlation-based networks, to identify a possible enrichment of miRNAs related to the proposed biological function. Additionally, we compared our miRNA-metagenes to *miRCancer* <http://mircancer.ecu.edu/> [7].

In order to explore the link or edges between MICs and RICs we extracted the target genes with a strong support from *miRTarBase* (<http://mirtarbase.mbc.nctu.edu.tw/php/index.php>, [8]) for those miRNA-metagenes mapping the miRNAs and clusters found by literature mining. Additionally, we filtered these target genes to ensure that they were part of the reference gene set based on top-contributing genes as determined by the mRNA-reference set. We then overlapped these target genes with the metagenes of the respective linked RIC and applied *Enrichr* tool through the automated Python-based API using the following reference gene set collections: KEGG_2016, GO_Biological_Process_2017b, GO_Cellular_Component_2017b, Jensen_TISSUES, Jensen_DISEASES (script available on demand). Moreover, we explored the overlapping target- and metagenes by STRING (<https://string-db.org/>, [9]) to detect significantly enriched protein-protein interaction networks. Both the results of the link-analysis between RICs and MICs as well as the investigation of MIC-metagenes have been finalised by visual inspection and evaluation by biological experts. A schematic illustration is shown in Additional file 3, Figure S1.

## REFERENCES

[1] Bogunovic D, O'Neill DW, Belitskaya-Levy I, Vacic V, Yu YL, Adams S, et al. Immune profile and mitotic index of metastatic melanoma lesions enhance clinical staging in predicting patient survival. Proc Natl Acad Sci U S A 2009;106:20429-34.

[2] Nazarov PV, Muller A, Kaoma T, Nicot N, Maximo C, Birembaut P, et al. RNA sequencing and transcriptome arrays analyses show opposing results for alternative splicing in patient derived samples. BMC Genomics 2017;18:443.

[3] Cancer Genome Atlas N. Genomic Classification of Cutaneous Melanoma. Cell 2015;161:1681-96.

[4] Anders S, Huber W. Differential expression analysis for sequence count data. Genome Biol 2010;11:R106.

[5] Nazarov PV, Gobin M, Zinovyev A, van Dyck E, Vallar L. Decomposition of transcriptional signal from tumours using independent component analysis. 15th European Conference on Computational Biology (ECCB) 2016.

[6] Kairov U, Cantini L, Greco A, Molkenov A, Czerwinska U, Barillot E, et al. Determining the optimal number of independent components for reproducible transcriptomic data analysis. BMC Genomics 2017;18:712.

[7] Xie B, Ding Q, Han H, Wu D. miRCancer: a microRNA-cancer association database constructed by text mining on literature. Bioinformatics 2013;29:638-44.

[8] Chou CH, Shrestha S, Yang CD, Chang NW, Lin YL, Liao KW, et al. miRTarBase update 2018: a resource for experimentally validated microRNA-target interactions. Nucleic Acids Res 2018;46:D296-D302.

[9] Szklarczyk D, Morris JH, Cook H, Kuhn M, Wyder S, Simonovic M, et al. The STRING database in 2017: quality-controlled protein-protein association networks, made broadly accessible. Nucleic Acids Res 2017;45:D362-D8.
