# Supplementary material for: Deconvolution of transcriptomes and miRNomes by independent component analysis provides insights into biological processes and clinical outcomes of melanoma patients
Source: BMC Med Genomics. 2019 Sep 18;12:132. doi: 10.1186/s12920-019-0578-4 (PMC6751789; doi:10.1186/s12920-019-0578-4)

Supplementary Results

This is an automatic report, generated for the components discussed in the paper by the consensus ICA tool (consICA)

https://gitlab.com/biomodlih/consica

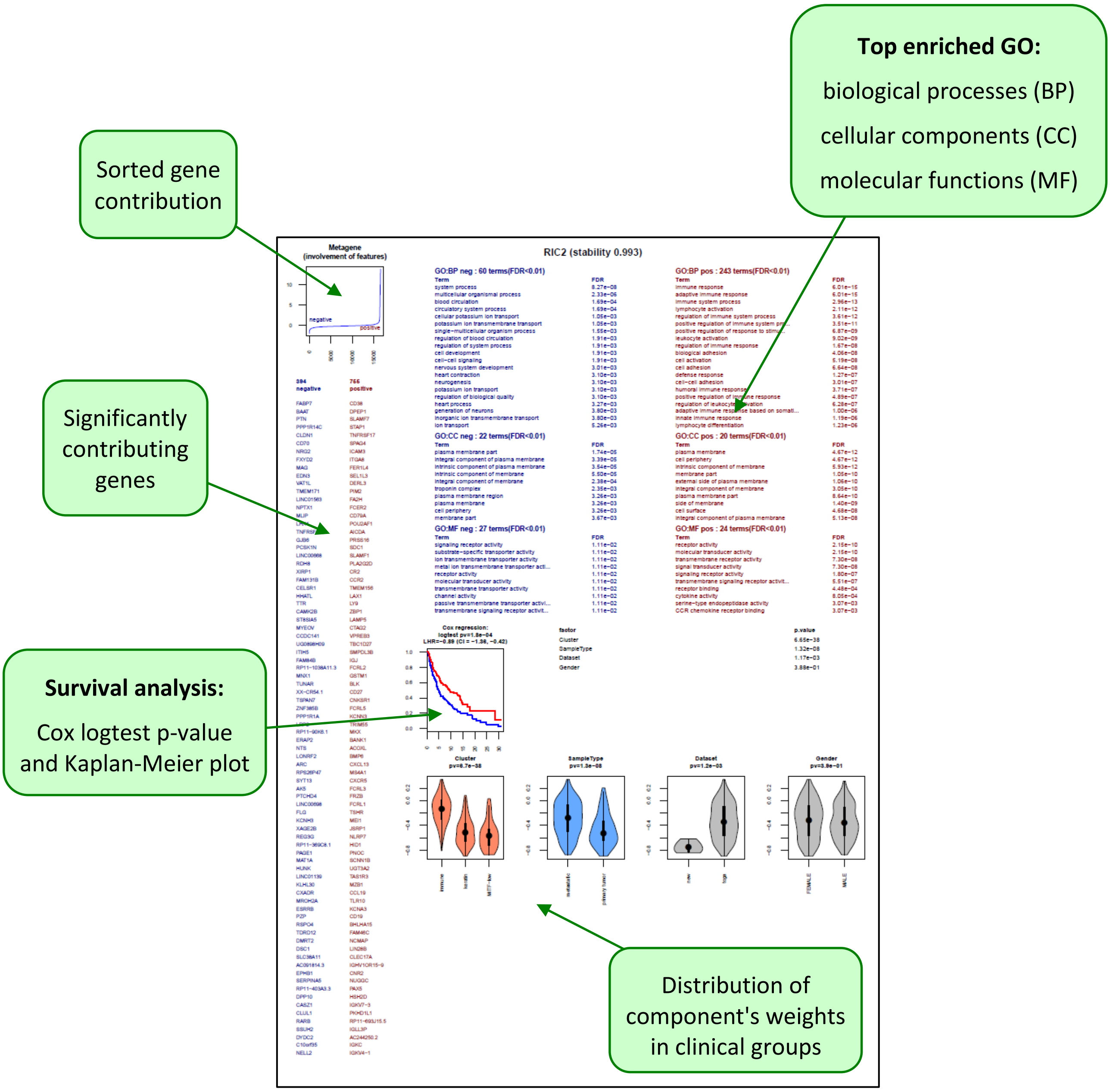

RIC2 (stability 0.993)

Metagene
(involvement of features)

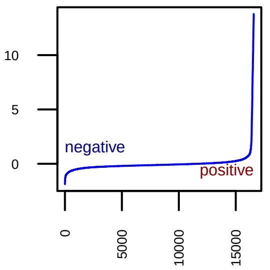

394 negative	755 positive
FABP7	CD38
BAAT	DPEP1
PTN	SLAMF7
PPP1R14C	STAP1
CLDN1	TNFRSF17
CD70	SPAG4
NRG2	ICAM3
FXYD2	ITGA8
MAG	FER1L4
EDN3	SEL1L3
VAT1L	DERL3
TMEM171	PIM2
LINC01563	FA2H
NPTX1	FCER2
MLIP	CD79A
LHX1	POU2AF1
TNFRSF10D	AICDA
GJB6	PRSS16
PCSK1N	SDC1
LINC00668	SLAMF1
RDH8	PLA2G2D
XIRP1	CR2
FAM131B	CCR2
CELSR1	TMEM156
HHATL	LAX1
TTR	LY9
CAMK2B	ZBP1
ST8SIA5	LAMP5
MYEOV	CTAG2
CCDC141	VPREB3
UG0898H09	TBC1D27
ITIH5	SMPDL3B
FAM84B	IGJ
RP11−1038A11.3	FCRL2
MNX1	GSTM1
TUNAR	BLK
XX−CR54.1	CD27
TSPAN7	CNKSR1
ZNF385B	FCRL5
PPP1R1A	KCNN3
LRP2	TRIM55
RP11−90K6.1	MKX
ERAP2	BANK1
NTS	ACOXL
LONRF2	BMP6
ARC	CXCL13
RPS26P47	MS4A1
SYT13	CXCR5
AK5	FCRL3
PTCHD4	FRZB
LINC00698	FCRL1
FLG	TSHR
KCNH3	MEI1
XAGE2B	JSRP1
REG3G	NLRP7
RP11−369C8.1	HID1
PAGE1	PNOC
MAT1A	SCNN1B
HUNK	UGT3A2
LINC01139	TAS1R3
KLHL30	MZB1
CXADR	CCL19
MROH2A	TLR10
ESRRB	KCNA3
PZP	CD19
RSPO4	BHLHA15
TDRD12	FAM46C
DMRT2	NCMAP
DSC1	LIN28B
SLC38A11	CLEC17A
AC091814.3	IGHV1OR15−9
EPHB1	CNR2
SERPINA5	NUGGC
RP11−403A3.3	PAX5
DPP10	HSH2D
CASZ1	IGKV7−3
CLUL1	PKHD1L1
RARB	RP11−693J15.5
SSUH2	IGLL3P
DYDC2	AC244250.2
C10orf35	IGKC
NELL2	IGKV4−1

GO:BP neg : 60 terms(FDR<0.01)

Term	FDR
system process	8.27e−08
multicellular organismal process	2.33e−06
blood circulation	1.69e−04
circulatory system process	1.69e−04
cellular potassium ion transport	1.05e−03
potassium ion transmembrane transport	1.05e−03
single−multicellular organism process	1.55e−03
regulation of blood circulation	1.91e−03
regulation of system process	1.91e−03
cell development	1.91e−03
cell−cell signaling	1.91e−03
nervous system development	3.01e−03
heart contraction	3.10e−03
neurogenesis	3.10e−03
potassium ion transport	3.10e−03
regulation of biological quality	3.10e−03
heart process	3.27e−03
generation of neurons	3.80e−03
inorganic ion transmembrane transport	3.80e−03
ion transport	5.26e−03

GO:BP pos : 243 terms(FDR<0.01)

Term	FDR
immune response	6.01e−15
adaptive immune response	6.01e−15
immune system process	2.96e−13
lymphocyte activation	2.11e−12
regulation of immune system process	3.61e−12
positive regulation of immune system pro...	3.51e−11
positive regulation of response to stimu...	6.87e−09
leukocyte activation	9.02e−09
regulation of immune response	1.67e−08
biological adhesion	4.06e−08
cell activation	5.19e−08
cell adhesion	6.64e−08
defense response	1.27e−07
cell−cell adhesion	3.01e−07
humoral immune response	3.71e−07
positive regulation of immune response	4.89e−07
regulation of leukocyte activation	6.28e−07
adaptive immune response based on somati...	1.00e−06
innate immune response	1.19e−06
lymphocyte differentiation	1.23e−06

GO:CC neg : 22 terms(FDR<0.01)

Term	FDR
plasma membrane part	1.74e−05
integral component of plasma membrane	3.39e−05
intrinsic component of plasma membrane	3.54e−05
intrinsic component of membrane	5.50e−05
integral component of membrane	2.38e−04
troponin complex	2.35e−03
plasma membrane region	3.26e−03
plasma membrane	3.26e−03
cell periphery	3.26e−03
membrane part	3.67e−03

GO:CC pos : 20 terms(FDR<0.01)

Term	FDR
plasma membrane	4.67e−12
cell periphery	4.67e−12
intrinsic component of membrane	5.93e−12
membrane part	1.05e−10
external side of plasma membrane	1.06e−10
integral component of membrane	3.05e−10
plasma membrane part	8.64e−10
side of membrane	1.40e−09
cell surface	4.68e−08
integral component of plasma membrane	5.13e−08

GO:MF neg : 27 terms(FDR<0.01)

Term	FDR
signaling receptor activity	1.11e−02
substrate−specific transporter activity	1.11e−02
ion transmembrane transporter activity	1.11e−02
metal ion transmembrane transporter acti...	1.11e−02
receptor activity	1.11e−02
molecular transducer activity	1.11e−02
transmembrane transporter activity	1.11e−02
channel activity	1.11e−02
passive transmembrane transporter activi...	1.11e−02
transmembrane signaling receptor activit...	1.11e−02

GO:MF pos : 24 terms(FDR<0.01)

Term	FDR
receptor activity	2.15e−10
molecular transducer activity	2.15e−10
transmembrane receptor activity	7.30e−08
signal transducer activity	7.30e−08
signaling receptor activity	1.80e−07
transmembrane signaling receptor activit...	5.51e−07
receptor binding	4.48e−04
cytokine activity	8.05e−04
serine−type endopeptidase activity	3.07e−03
CCR chemokine receptor binding	3.07e−03

Cox regression:
logtest pv=1.8e−04
LHR=−0.89 (CI = −1.36, −0.42)

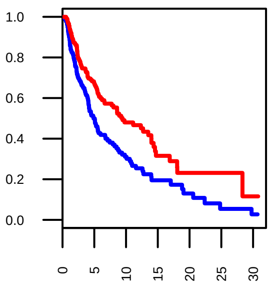

factor	p.value
Cluster	6.65e−38
SampleType	1.32e−08
Dataset	1.17e−03
Gender	3.88e−01

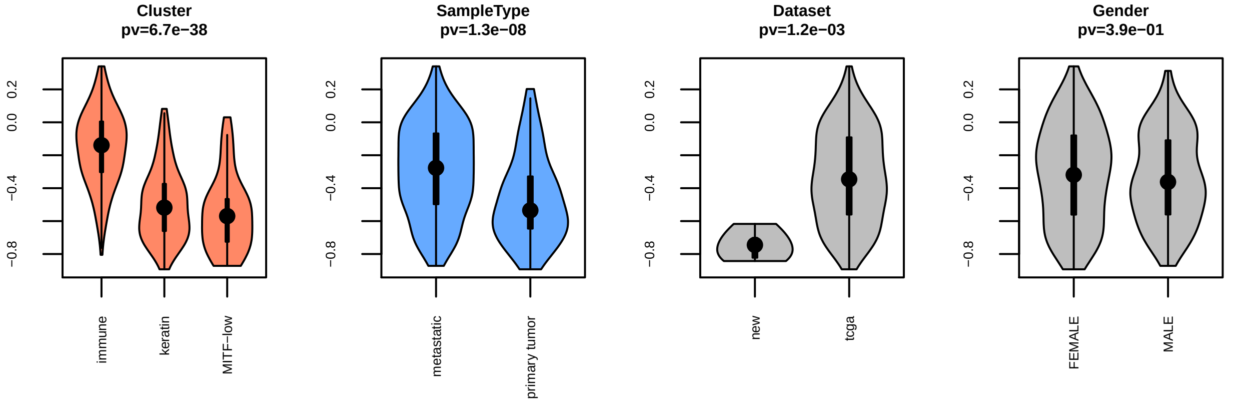

RIC3 (stability 0.995)

Metagene
(involvement of features)

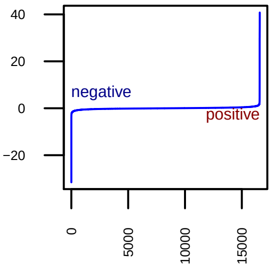

498 negative	431 positive
XIST	KDM5D
PAGE2	DDX3Y
PPP1R3C	ZFY
ASB11	ADCY2
HERC2P3	DDX43
MAGEB1	PRKY
NLRP2	USP9Y
PAGE2B	POMC
PRSS21	EDN3
CNTN3	RPS4Y1
MAGEB2	TXLNGY
PCSK2	NLGN4Y
CPSF1P1	TSPY2
COL9A1	UTY
SLITRK6	EIF1AY
MKRN3	GYG2P1
CRABP1	IGHV1−69
PDLIM4	RP11−98L5.4
VCX	RP4−610C12.3
HOXD11	TTTY15
TDRD12	RBMY2QP
HIST1H2AE	RP11−333A23.4
RP11−299H22.3	OVCH1−AS1
HOXC12	SPESP1
ENPP5	TSPY1
HOXD10	LHX1
ABHD12B	RPS4Y2
PSPHP1	SH3GL2
XG	CTC−523E23.5
CCL13	SCN7A
RP1−90G24.6	RP11−12M5.3
CSMD1	HAMP
AC016708.2	CTNNA2
DSG2	MARVELD2
FAM178B	PEX5L
HCN1	SYN2
AL035610.1	TMEM233
CXCL1	XAGE2B
CCL22	RP11−1109M24.5
KLK2	MAGEC2
HDHD1	FAM163A
ZRSR2	NRK
SSX1	RP11−575F12.3
CXCL8	RP4−644L1.2
APOC2	ADARB2
KDM6A	TRPM6
PCDHGB5	RP11−63E9.1
UGT2B7	TSPAN8
HOXB8	HORMAD1
DDX3X	PCDHGA12
RP11−60L3.1	SLC30A8
SYT5	RP11−529E10.7
FDCSP	IGHG4
GYG2	RP4−610C12.4
MYH14	ERAP2
FLJ36000	RP11−488I20.8
GEMIN8	LINC00944
TSPYL5	MUC15
ADAMTS16	FBXL21
THNSL2	CBLN4
CA5BP1	CTD−2380F24.1
ZFX	PTCHD3
ROS1	MKRN9P
RP11−706O15.1	HS3ST2
HAS1	CSF2RA
RPS4X	SLC24A4
COL11A2	RP11−599J14.2
RAB33A	SIM2
GRPR	CACNA1A
TRAPPC2	EVA1A
ARSD	RP11−66B24.7
NLGN4X	NPPC
SYT1	EN2
PCDHGA3	CSDC2
MYL10	TRABD2B
HOXD13	COCH
ACAN	SAMD5
RP11−431J24.2	HDHD3
VCX3A	ENKUR
HPSE2	CAP2
GLRA2	KIF25
RP11−218E20.3	TBX18

GO:BP neg : 24 terms(FDR<0.01)

Term	FDR
embryonic skeletal system development	2.11e−02
embryonic skeletal system morphogenesis	2.11e−02
multicellular organismal process	2.14e−02
anatomical structure development	2.14e−02
developmental process	2.17e−02
anterior/posterior pattern specification	2.36e−02
antimicrobial humoral response	2.82e−02
chemokine−mediated signaling pathway	2.82e−02
antibacterial humoral response	3.91e−02
positive regulation of granulocyte chemo...	3.91e−02
single−organism developmental process	5.26e−02
G−protein coupled receptor signaling pat...	5.26e−02
adult behavior	5.48e−02
nervous system development	5.48e−02
serotonin receptor signaling pathway	5.48e−02
G−protein coupled serotonin receptor sig...	5.48e−02
multicellular organism development	5.48e−02
embryonic organ morphogenesis	6.26e−02
chemical synaptic transmission, postsyna...	6.63e−02
system process	6.63e−02

GO:BP pos : 5 terms(FDR<0.01)

Term	FDR
multicellular organismal process	2.41e−03
single−multicellular organism process	5.04e−03
cell−cell signaling	8.42e−02
neurological system process	8.42e−02
anatomical structure development	8.42e−02

GO:CC neg : 11 terms(FDR<0.01)

Term	FDR
proteinaceous extracellular matrix	2.47e−03
extracellular matrix	2.47e−03
nucleosome	2.75e−03
DNA packaging complex	6.60e−03
integral component of plasma membrane	6.60e−03
intrinsic component of plasma membrane	8.86e−03
extracellular region part	3.05e−02
extracellular region	3.05e−02
extracellular space	3.05e−02
collagen trimer	5.87e−02

GO:CC pos : 13 terms(FDR<0.01)

Term	FDR
neuron part	9.17e−03
cell periphery	9.17e−03
extracellular space	9.17e−03
integral component of plasma membrane	9.17e−03
plasma membrane	1.24e−02
presynapse	1.24e−02
plasma membrane part	1.24e−02
intrinsic component of plasma membrane	1.24e−02
perikaryon	1.75e−02
synapse	2.67e−02

GO:MF neg : 22 terms(FDR<0.01)

Term	FDR
chemokine activity	6.01e−03
extracellular matrix structural constitu...	1.00e−02
extracellular matrix structural constitu...	1.00e−02
chemokine receptor binding	1.00e−02
cytokine activity	1.00e−02
CXCR chemokine receptor binding	1.00e−02
transmembrane receptor activity	1.91e−02
signaling receptor activity	1.91e−02
neurotransmitter receptor activity	1.91e−02
G−protein coupled receptor binding	1.91e−02

GO:MF pos : 1 terms(FDR<0.01)

Term	FDR
hormone activity	4.72e−02

Cox regression:
logtest pv=1.9e−01
LHR=0.72 (CI = −0.35, 1.79)

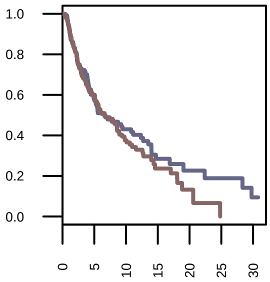

factor	p.value
Gender	3.25e−226
Dataset	1.61e−01
Cluster	2.11e−01
SampleType	2.16e−01

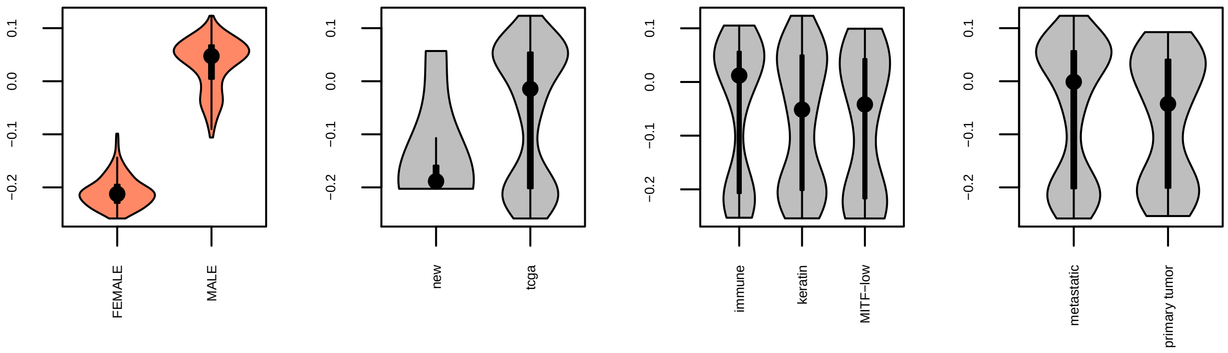

RIC4 (stability 0.935)

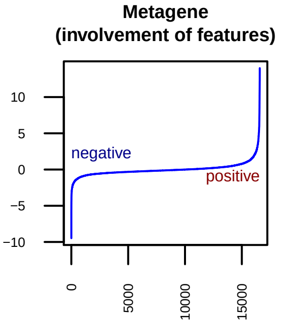

359 negative	875 positive
MAGEA4	ABCB5
MAEL	ASB4
RP11−366F6.2	CDH1
PASD1	USH2A
PRSS21	MAGEC2
ILDR2	MCOLN3
CTCFL	RASGRF1
VAX1	CDH3
RP11−1220K2.2	CTNNA2
CT45A1	TYR
AP001065.15	DCT
MAGEB2	TDRD3
CLEC2L	RPH3A
TDRD9	BIRC7
SOHLH1	GPR143
FAR2P1	BMX
FREM1	LUZP4
SAGE1	GAPDHS
PAGE1	TYRP1
DLX6	BCHE
P3H2	MORC1
COL9A1	LPPR5
PNMA5	MMP8
ASPG	MLANA
RAP1GAP2	LDB3
CT45A10	IL13RA2
LY6K	TRIM51
LRP1B	PRDM7
DLX6−AS1	OR7C1
DPEP3	DPP6
HOXC12	FCRLA
SLC6A10P	ALDH3B2
CADM3	DYDC2
DMRT2	TRPM1
TCAM1P	SYT6
LHFPL4	TCN1
POU6F2	HRK
ZFR2	BAAT
SYNM	DNASE2B
FRMPD3	CYP1A1
EXTL1	SPACA3
XDH	CHRNA6
SAMD12	SNTG1
COL4A5	PMP2
TLX1	TRIM48
ALDH1A3	MAT1A
LINC01019	CYSLTR2
MAPK4	GRM1
SERPINA5	MAGEC1
MAGEA10	KIT
GAGE2A	TRIM63
ITGA9	IP6K3
LOXL4	WDR63
LPAR1	LRRTM1
SFRP5	XIRP2
PLCXD3	SYNPR
ENTPD2	HPGD
DISP2	SLC45A2
TUNAR	GPR98
KANK4	BAALC
KCNS1	ASB11
GCNT1	KCNC2
HNF4G	PKD1L2
MAGEA8	MS4A8
WNK4	D4S234E
NACAD	FSTL5
PDE1C	PCSK9
MIR4697HG	IL1RAPL1
TDRD12	UGT2B7
RP3−410C9.2	DEC1
GPR12	GALNTL6
ARHGEF4	OR9G1
ADARB2	KLHL38
CCDC141	ENTHD1
CH17−437K3.1	RIMS2
UG0898H09	NCKAP5
ISL1	CA8
FOXG1	ANKRD30B
GALNT8	NXPH4
RGS9	LINC00518
MAPK15	KRTAP19−1
TCF24	ROBO2

GO:BP neg : 97 terms(FDR<0.01)

Term	FDR
multicellular organismal process	7.22e−08
single−multicellular organism process	1.28e−06
nervous system development	1.25e−05
anatomical structure development	2.44e−05
multicellular organism development	4.21e−05
developmental process	2.36e−04
neurological system process	2.36e−04
single−organism developmental process	2.44e−04
anatomical structure morphogenesis	2.84e−04
system development	3.46e−04
negative regulation of neurogenesis	1.08e−03
generation of neurons	1.50e−03
cell development	1.90e−03
regulation of neurogenesis	1.90e−03
locomotory behavior	1.90e−03
negative regulation of nervous system de...	2.26e−03
regulation of nervous system development	2.39e−03
neurogenesis	3.26e−03
animal organ morphogenesis	3.98e−03
negative regulation of cell development	3.98e−03

GO:CC neg : 25 terms(FDR<0.01)

Term	FDR
neuron part	5.13e−03
proteinaceous extracellular matrix	6.97e−03
extracellular matrix	1.16e−02
neuronal cell body	2.66e−02
cell body	4.40e−02
cation channel complex	4.58e−02
ion channel complex	4.58e−02
integral component of plasma membrane	4.58e−02
somatodendritic compartment	4.58e−02
synapse part	4.58e−02

GO:MF neg : 2 terms(FDR<0.01)

Term	FDR
gated channel activity	5.58e−02
metal ion transmembrane transporter acti...	9.23e−02

GO:BP pos : 81 terms(FDR<0.01)

Term	FDR
sensory perception	5.26e−08
multicellular organismal process	1.28e−07
system process	4.21e−06
neurological system process	7.14e−06
phenol−containing compound biosynthetic ...	1.23e−05
melanin biosynthetic process	1.23e−05
melanin metabolic process	2.02e−05
secondary metabolite biosynthetic proces...	3.20e−05
ion transmembrane transport	9.19e−05
system development	9.62e−05
developmental pigmentation	1.03e−04
transmembrane transport	1.03e−04
single−multicellular organism process	1.39e−04
visual perception	1.40e−04
sensory perception of light stimulus	1.90e−04
secondary metabolic process	3.10e−04
single−organism process	4.33e−04
multicellular organism development	7.43e−04
detection of stimulus involved in sensor...	7.76e−04
ion transport	1.05e−03

GO:CC pos : 15 terms(FDR<0.01)

Term	FDR
plasma membrane	4.77e−13
cell periphery	4.77e−13
intrinsic component of membrane	1.77e−12
integral component of membrane	8.71e−12
membrane part	2.24e−09
plasma membrane part	2.47e−09
integral component of plasma membrane	1.20e−08
intrinsic component of plasma membrane	1.88e−08
melanosome membrane	3.30e−04
chitosome	3.30e−04

GO:MF pos : 28 terms(FDR<0.01)

Term	FDR
transmembrane transporter activity	3.69e−06
channel activity	8.87e−06
passive transmembrane transporter activi...	8.87e−06
transporter activity	3.76e−05
calcium ion binding	7.04e−05
substrate−specific channel activity	7.87e−05
signaling receptor activity	2.15e−04
ion transmembrane transporter activity	2.15e−04
ion channel activity	2.97e−04
receptor activity	2.97e−04

Cox regression:
logtest pv=5.4e−03
LHR=1.13 (CI = 0.32, 1.94)

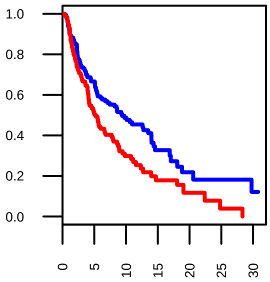

factor	p.value
Cluster	7.28e−14
SampleType	2.33e−05
Dataset	8.09e−02
Gender	2.46e−01

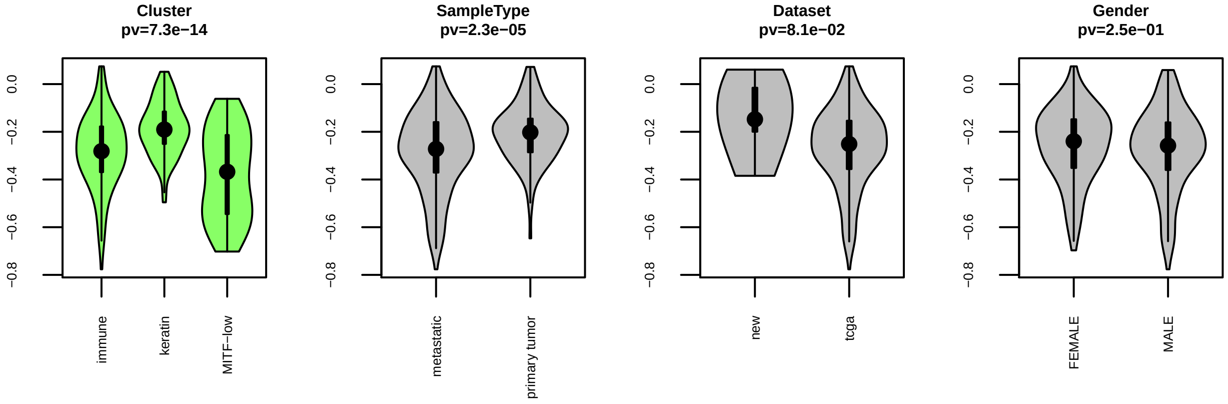

RIC5 (stability 0.993)

Metagene
(involvement of features)

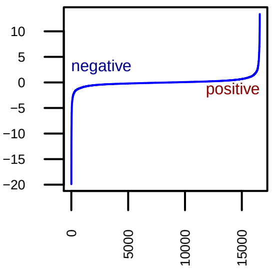

685 negative	779 positive
BPIFB1	WNT16
KRT4	DLX3
KRT19	PPP2R2C
KRT13	HAL
MUC21	APOB
CEACAM6	SLC15A1
SPRR3	PLA2G3
PIGR	SALL1
CRNN	STMN2
MUC4	EPHX3
STATH	CASP14
SLC34A2	SLC1A6
DMBT1	CNFN
AGR2	FOXN1
CEACAM5	CWH43
SFTPB	ELMOD1
BPIFB2	UNC93A
PRH2	IRX4
KRT7	ARG1
PRB3	TP53AIP1
ELF3	LYPD3
CXCL17	FAM83C
FOLR1	SLURP1
SCGB3A1	TUBA4A
PIP	CPA4
WFDC2	BBOX1
LCN2	KLK8
PRB4	KRT34
SCGB3A2	SLC6A11
NTS	RHBG
SCGB1A1	DSG1
MUC16	DSC1
SFTPA2	IL36G
CLCA4	IL36RN
MUC5B	POU2F3
GSTA1	RDH12
TMPRSS2	IL22RA1
TMPRSS11A	CRABP2
PPP1R1B	FLG2
SFTPA1	S100A7
DUOX2	FLG
CXCL6	ABCA12
TSPAN1	GSDMC
CTSE	CDHR1
UPK1B	LYPD6B
EPCAM	PLA2G2F
DSG3	CDA
KRT6A	PLA2G4D
CYP4B1	LCE2B
TMPRSS11E	SPRR2G
MMP7	PGLYRP3
FAM3D	DMKN
TGM3	HS3ST6
CLDN3	LCE3D
KLK3	PGLYRP4
SLPI	S100A9
LTF	ELOVL7
NKX2−1	GPR111
MB	RAET1E
KRT5	AMER2
OLFM4	SLC39A2
LPO	RNASE7
BMP3	SERPINA12
LIPH	ASPG
KRT15	SERPINB7
ZG16B	SERPINB12
KRT8	HTR3A
MS4A15	CHP2
MMP12	KLK5
ODAM	KRT1
SPDEF	ACER1
SLC44A4	GSDMA
CST5	DNASE1L2
GABRP	WFDC12
PAX9	PLA2G4F
VSIG2	KLK7
CLDN10	COL22A1
TRIM29	CRCT1
MYH7	SDR9C7
WIF1	KRT75
FAM3B	KRT9
FDCSP	CYP4F22

GO:BP neg : 294 terms(FDR<0.01)

Term	FDR
multicellular organismal process	2.71e−17
single−multicellular organism process	8.27e−14
tissue development	5.01e−12
system process	1.39e−10
anatomical structure development	4.81e−10
developmental process	4.26e−09
single−organism developmental process	4.73e−09
system development	8.46e−09
epithelial cell differentiation	1.65e−08
multicellular organism development	1.65e−08
epithelium development	1.16e−07
anatomical structure morphogenesis	1.38e−07
humoral immune response	1.85e−07
sensory perception	1.72e−06
neurological system process	2.51e−06
antimicrobial humoral response	3.80e−06
animal organ development	3.80e−06
defense response to bacterium	5.60e−06
cornification	8.71e−06
negative regulation of peptidase activit...	1.50e−05

GO:BP pos : 77 terms(FDR<0.01)

Term	FDR
epidermis development	2.51e−28
keratinocyte differentiation	2.51e−28
skin development	2.51e−28
epidermal cell differentiation	2.51e−28
keratinization	2.51e−28
cornification	2.51e−28
epithelial cell differentiation	9.02e−26
peptide cross−linking	4.70e−25
epithelium development	1.55e−15
tissue development	6.32e−13
cell differentiation	1.37e−07
single−multicellular organism process	3.38e−07
multicellular organismal process	5.44e−07
cellular developmental process	7.20e−07
establishment of skin barrier	1.80e−06
regulation of water loss via skin	5.83e−06
anatomical structure development	1.24e−05
animal organ development	2.84e−05
antimicrobial humoral response	6.25e−05
defense response to bacterium	7.22e−05

GO:CC neg : 48 terms(FDR<0.01)

Term	FDR
extracellular region	9.17e−29
extracellular space	9.17e−29
extracellular region part	1.47e−23
cell periphery	7.79e−09
plasma membrane	1.06e−08
extracellular exosome	1.26e−08
plasma membrane part	1.26e−08
extracellular organelle	1.34e−08
extracellular vesicle	1.34e−08
proteinaceous extracellular matrix	1.08e−07

GO:CC pos : 26 terms(FDR<0.01)

Term	FDR
cornified envelope	1.83e−28
extracellular region	8.43e−12
plasma membrane	6.72e−11
extracellular space	7.79e−11
cell periphery	9.53e−11
intermediate filament	1.83e−06
intermediate filament cytoskeleton	7.33e−06
extracellular region part	4.81e−05
epidermal lamellar body	8.15e−04
keratin filament	1.25e−03

GO:MF neg : 40 terms(FDR<0.01)

Term	FDR
serine−type endopeptidase activity	4.64e−05
endopeptidase inhibitor activity	4.64e−05
peptidase inhibitor activity	4.64e−05
peptidase regulator activity	4.64e−05
endopeptidase regulator activity	4.64e−05
serine−type peptidase activity	2.00e−04
serine hydrolase activity	2.45e−04
receptor binding	4.35e−04
cytokine activity	1.00e−03
heparin binding	1.16e−03

GO:MF pos : 48 terms(FDR<0.01)

Term	FDR
structural molecule activity	6.01e−07
structural constituent of epidermis	4.08e−04
serine−type endopeptidase inhibitor acti...	1.22e−03
endopeptidase inhibitor activity	1.50e−03
peptidase inhibitor activity	1.98e−03
endopeptidase regulator activity	2.08e−03
lipase activity	3.19e−03
alcohol binding	2.48e−02
sterol binding	2.48e−02
metal ion transmembrane transporter acti...	2.52e−02

Cox regression:
logtest pv=5.8e−03
LHR=1.54 (CI = 0.52, 2.56)

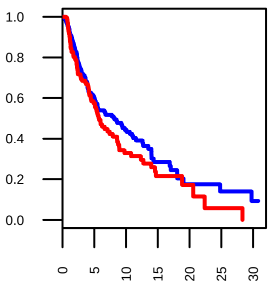

factor	p.value
SampleType	1.05e−43
Cluster	6.35e−16
Dataset	1.35e−04
Gender	6.14e−01

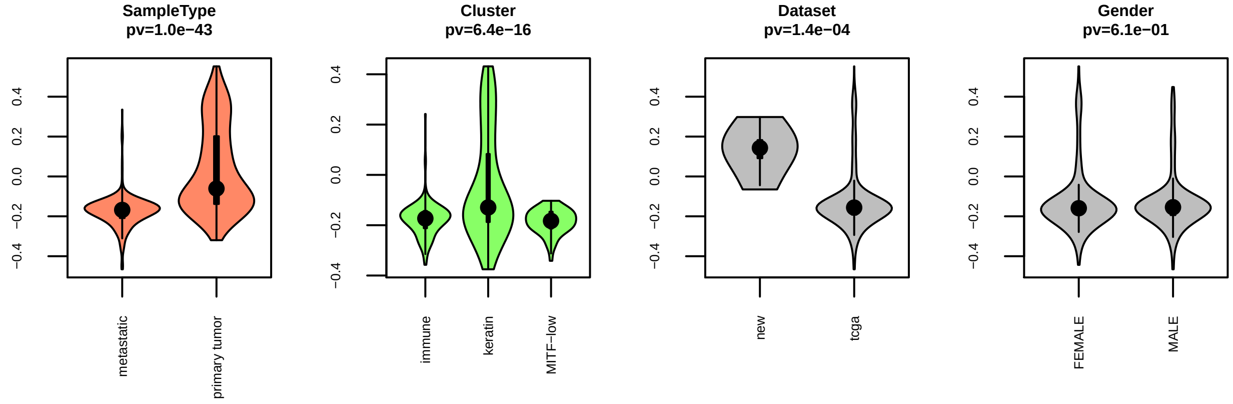

RIC6 (stability 0.593)

Metagene (involvement of features)

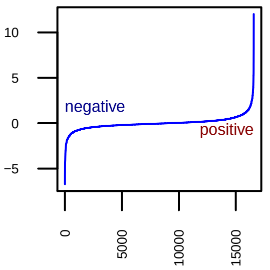

600 negative	886 positive
FRG2DP	KLHL13
CA8	ASB4
TYRP1	HHATL
OCA2	MYOC
GRIK2	CDH1
MGAT5B	MCOLN3
TRIM48	MPPED2
PMP2	PHKA1
ROBO2	C1QTNF3
RNF182	TFAP2C
PAEP	FXYD3
MAL	TF
SLC38A8	CYP26A1
SLC16A6	CRTAC1
AC010967.2	HCN2
ABCB4	GLRA2
LINC01198	PRSS33
FSTL5	CORO2B
SLC7A4	EYA1
WIPF3	SFRP1
RP6−24A23.7	HPN
ST8SIA2	SCN1B
SLC5A4	PPARGC1A
LINC00052	MGP
DLGAP1	TPD52L1
BMPR1B	EVA1A
WDR72	OLFM3
INSC	SPP1
LYPD1	PYROXD2
LINC01502	GJB6
MLIP	HTN1
ITGB8	SSX1
SUSD5	ATP1B2
COBL	RNASE1
COL19A1	VSTM2L
CHADL	LGALS12
NPPC	TEX15
ENTHD1	CRB1
RP11−63E9.1	TBX3
KCNS3	AMHR2
IRS4	PI15
RP6−24A23.3	LOXL4
SLAMF9	PRKG2
DPP6	FRAS1
ADCY2	ITGA10
DKK1	ATP1B1
OPRD1	REG3G
PHACTR3	PLA1A
NCAM2	RP11−35N6.1
NOV	HMGA2
FCRLA	CLEC1A
ARX	LGI4
CTNND2	CHST9
PRUNE2	C4orf19
MTCL1	PIEZO2
GPM6A	CNTNAP5
PRSS12	GRIP1
SLITRK5	TSPAN7
SLC24A4	HKDC1
LCE2A	NBL1
LINC01293	HOXB13
MMP8	UROC1
AC108142.1	SLC9C2
NR4A3	CADPS
CLDN14	ETNPPL
EPS8L2	HPGD
GFRA3	HCN1
CFAP61	FREM1
CXADR	ASB11
DNAJC6	GJB2
KRTAP19−1	NELL1
KCNN2	KCNC2
KCNH1	MS4A8
WDR17	MYO5B
SLC35F1	TTYH1
DNER	GDNF
RAMP1	PCDH7
ABCC2	TTLL6
RP11−317M11.1	SOSTDC1
EPHA8	AQP4
CCDC171	CYP7B1
PCYT1B	LRRTM4

GO:BP neg : 50 terms(FDR<0.01)

Term	FDR
multicellular organismal process	5.11e−04
generation of neurons	7.02e−04
neuron differentiation	7.02e−04
neuron projection development	7.27e−04
neurogenesis	7.27e−04
neuron development	7.27e−04
single−organism process	8.81e−04
anatomical structure development	1.84e−03
sensory perception	1.84e−03
multicellular organism development	2.41e−03
nervous system development	2.91e−03
axon development	2.91e−03
G−protein coupled receptor signaling pat...	2.91e−03
cell morphogenesis involved in neuron di...	2.91e−03
single−multicellular organism process	2.91e−03
cell projection morphogenesis	2.91e−03
neurological system process	3.34e−03
neuron projection morphogenesis	3.34e−03
cell adhesion	4.59e−03
biological adhesion	4.99e−03

GO:CC neg : 55 terms(FDR<0.01)

Term	FDR
intrinsic component of membrane	1.78e−13
integral component of membrane	1.19e−11
membrane part	8.55e−11
intrinsic component of plasma membrane	2.70e−09
integral component of plasma membrane	4.03e−09
cell periphery	7.33e−09
plasma membrane	9.69e−09
plasma membrane part	1.24e−08
proteinaceous extracellular matrix	1.38e−06
neuron part	8.07e−05

GO:MF neg : 8 terms(FDR<0.01)

Term	FDR
receptor activity	3.86e−11
molecular transducer activity	3.86e−11
signaling receptor activity	4.87e−08
transmembrane receptor activity	3.01e−07
transmembrane signaling receptor activit...	3.01e−07
signal transducer activity	6.94e−06
receptor binding	6.13e−04
G−protein coupled receptor activity	1.88e−02

GO:BP pos : 304 terms(FDR<0.01)

Term	FDR
multicellular organismal process	9.17e−14
single−multicellular organism process	1.43e−12
system development	1.45e−11
system process	1.05e−10
multicellular organism development	2.01e−10
single−organism process	3.51e−09
anatomical structure development	9.67e−09
single organism signaling	1.82e−08
signaling	2.17e−08
sensory perception	2.71e−08
cell communication	3.83e−08
anatomical structure morphogenesis	5.39e−08
regulation of multicellular organismal p...	7.63e−08
developmental process	8.38e−08
sensory perception of mechanical stimulu...	1.30e−07
single−organism developmental process	1.97e−07
animal organ development	4.68e−07
nervous system development	4.68e−07
neurological system process	6.01e−07
regulation of developmental process	1.58e−06

GO:CC pos : 43 terms(FDR<0.01)

Term	FDR
plasma membrane part	4.03e−13
cell periphery	1.28e−12
plasma membrane	1.83e−12
integral component of plasma membrane	3.02e−11
intrinsic component of plasma membrane	7.33e−11
extracellular space	1.19e−08
extracellular region	1.31e−08
intrinsic component of membrane	4.12e−08
integral component of membrane	2.85e−07
membrane part	1.15e−06

GO:MF pos : 49 terms(FDR<0.01)

Term	FDR
receptor activity	4.29e−06
molecular transducer activity	4.29e−06
metal ion transmembrane transporter acti...	4.29e−06
channel activity	9.20e−06
passive transmembrane transporter activi...	9.20e−06
transmembrane receptor activity	9.20e−06
substrate−specific channel activity	9.20e−06
ion channel activity	1.61e−05
cation channel activity	2.05e−05
transmembrane transporter activity	2.64e−05

Cox regression: logtest pv=5.5e−03 LHR=−1.45 (CI = −2.49, −0.41)

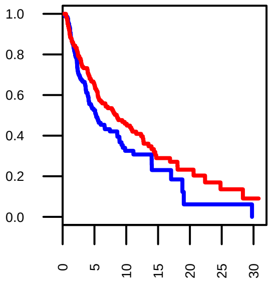

factor	p.value
Cluster	4.49e−06
SampleType	2.23e−02
Gender	7.25e−02
Dataset	4.75e−01

Cluster pv=4.5e−06

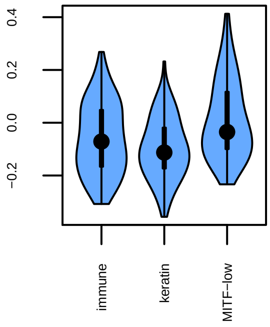

SampleType pv=2.2e−02

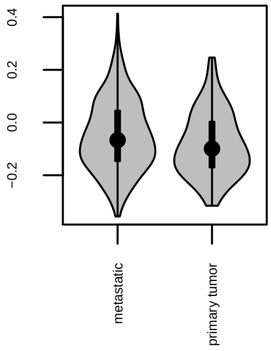

Gender pv=7.3e−02

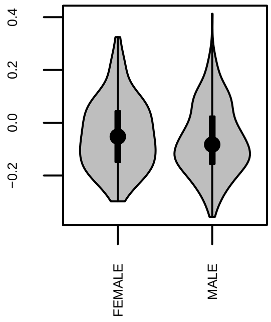

Dataset pv=4.7e−01

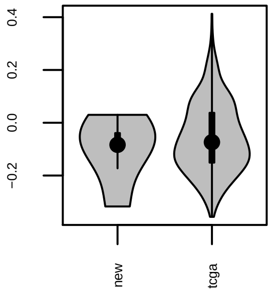

RIC7 (stability 0.996)

Metagene (involvement of features)

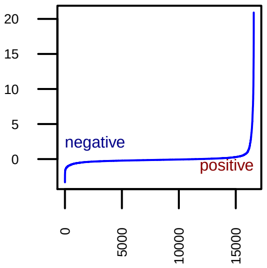

556 negative	908 positive
ADIPOQ	PRSS22
WT1	TTC22
PCK1	PLEKHG6
ADH1B	PRSS3
DLK1	IL20RA
FGA	CLCA4
C7	CYP24A1
ALB	RAB27B
FABP4	BARX2
GRIK2	EPN3
COL22A1	PRSS8
NRK	LAMA3
FGG	SERPINB3
TUSC5	PKP2
GSTM1	LAMC2
IGHV1−2	COL17A1
RBP4	FGFR2
APOA2	FGFR3
IGLV2−8	PITX1
DPP10	HES2
APOB	ST6GALNAC1
CR2	TP63
IGHV3−7	PPP2R2C
TIMP4	ATP12A
MAGEA10	CAMSAP3
HP	NEBL
GABRB3	BPIFB2
MME	PKP1
RP11−54O7.17	GRHL2
CHI3L1	HAL
PPIAP29	IGSF9
RIMS2	CEACAM6
RP1−309I22.2	FAT2
FCER2	TMPRSS11E
OGN	SULT2B1
CNTN4	SLC15A1
ASPA	TMEM40
WT1−AS	NOS1
SOX11	MYH7
CTNNA2	TGM1
RP3−333A15.1	GABRP
AMBP	KRT31
MMP9	DSP
PLIN1	PLA2G3
IGFBP1	SLC5A1
RP11−366L20.2	KCNK10
NLRP11	FERMT1
INHBB	OLFM4
DDIT4L	HAS3
FMN2	ESRP2
RP11−1220K2.2	TGM5
ZNF560	RHOV
RP11−475C16.1	KLC3
APOH	EPHX3
LINC01152	CASP14
AHSG	SLC1A6
PRG4	CEACAM5
TNFSF11	CNFN
SFRP4	AGR2
GATA4	KRT23
SYT14	ALDH3A1
LMO3	FOXN1
HS3ST5	CWH43
TMEM132D	SCNN1A
CDH15	ENDOU
ROBO2	FZD10
AC010970.2	UNC93A
C2orf70	IRX4
CBLN1	UPK1B
FGB	IL1A
PCDHGA12	ITGB6
CNTN6	IRF6
CYSLTR2	PPL
GFRA2	TP53AIP1
GABRB2	CSTA
FAM135B	GJB6
RPL41	PI3
VAT1L	SLPI
UCHL1	POF1B
TMEM100	LYPD3
CDO1	CXCL6
LOXL4	EREG

GO:BP neg : 317 terms(FDR<0.01)

Term	FDR
cell−cell signaling	2.11e−06
cell communication	2.18e−06
single organism signaling	3.42e−06
signaling	3.42e−06
multicellular organismal process	3.91e−06
system process	8.52e−06
hormone transport	9.24e−06
signal release	9.77e−06
hormone secretion	1.44e−05
regulation of hormone levels	1.50e−05
regulation of ion transport	2.05e−05
single−multicellular organism process	6.64e−05
response to external stimulus	1.39e−04
triglyceride metabolic process	3.22e−04
regulation of biological quality	4.11e−04
anatomical structure morphogenesis	4.70e−04
regulation of hormone secretion	4.86e−04
regulation of multicellular organismal p...	4.86e−04
regulation of system process	4.86e−04
neurological system process	4.86e−04

GO:BP pos : 165 terms(FDR<0.01)

Term	FDR
epidermis development	1.50e−28
keratinocyte differentiation	1.50e−28
skin development	1.50e−28
epidermal cell differentiation	1.50e−28
keratinization	1.50e−28
cornification	1.50e−28
epithelial cell differentiation	1.50e−28
epithelium development	1.50e−28
tissue development	1.50e−28
peptide cross−linking	1.50e−28
multicellular organismal process	1.91e−20
single−multicellular organism process	1.38e−18
cell differentiation	7.86e−16
animal organ development	2.15e−14
anatomical structure development	2.26e−14
system development	2.26e−14
cellular developmental process	2.39e−13
establishment of skin barrier	4.51e−13
cell−cell adhesion via plasma−membrane a...	1.11e−12
multicellular organism development	1.13e−12

GO:CC neg : 41 terms(FDR<0.01)

Term	FDR
extracellular space	3.67e−08
neuron projection terminus	2.75e−04
synapse part	2.75e−04
extracellular region	2.75e−04
synapse	3.97e−04
blood microparticle	3.97e−04
cell periphery	8.38e−04
synaptic membrane	1.44e−03
chylomicron	1.85e−03
presynapse	2.12e−03

GO:CC pos : 49 terms(FDR<0.01)

Term	FDR
cornified envelope	9.17e−29
extracellular region	9.17e−29
extracellular space	1.22e−26
cell periphery	4.58e−26
plasma membrane	2.71e−25
extracellular region part	6.11e−23
intermediate filament	5.50e−14
intermediate filament cytoskeleton	1.01e−13
plasma membrane part	4.48e−12
desmosome	4.95e−11

GO:MF neg : 23 terms(FDR<0.01)

Term	FDR
peptidase inhibitor activity	7.09e−03
receptor binding	7.09e−03
receptor activity	7.09e−03
molecular transducer activity	7.09e−03
endopeptidase inhibitor activity	7.13e−03
signaling receptor activity	7.87e−03
endopeptidase regulator activity	8.59e−03
high−density lipoprotein particle recept...	1.18e−02
peptidase regulator activity	2.42e−02
signal transducer activity	2.42e−02

GO:MF pos : 59 terms(FDR<0.01)

Term	FDR
structural molecule activity	3.86e−15
serine−type endopeptidase inhibitor acti...	8.73e−11
calcium ion binding	8.73e−11
serine−type endopeptidase activity	1.82e−10
serine−type peptidase activity	3.52e−10
serine hydrolase activity	5.37e−10
endopeptidase inhibitor activity	9.81e−09
peptidase inhibitor activity	2.04e−08
endopeptidase regulator activity	2.72e−08
structural constituent of epidermis	3.65e−07

Cox regression: logtest pv=8.9e−06 LHR=1.13 (CI = 0.68, 1.58)

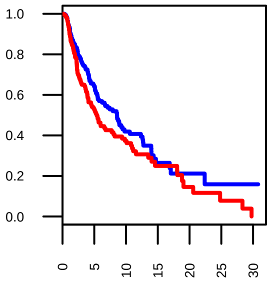

factor	p.value
SampleType	3.46e−40
Cluster	1.02e−23
Dataset	1.40e−04
Gender	1.46e−01

SampleType pv=3.5e−40

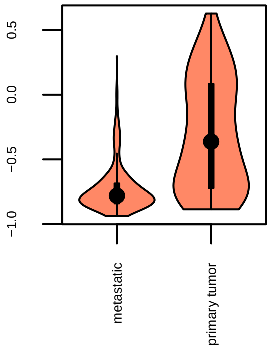

Cluster pv=1.0e−23

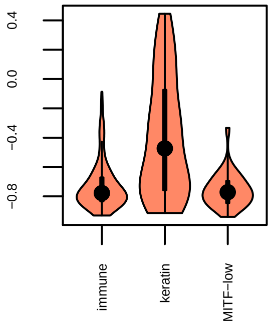

Dataset pv=1.4e−04

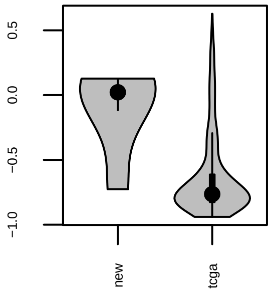

Gender pv=1.5e−01

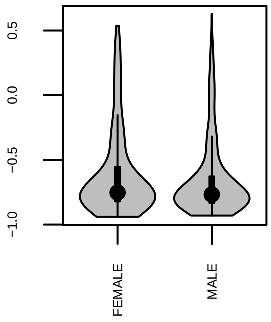

RIC13 (stability 0.977)

Metagene
(involvement of features)

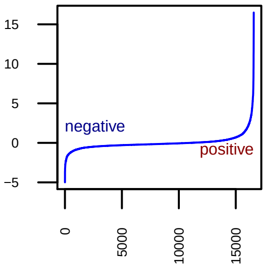

571 negative	1296 positive
SHISA3	TNMD
MT3	HSPB6
BMP7	MEOX1
MMP12	SELE
FOXF1	SEMA3G
FOXG1	FMO1
MAPK4	MRC2
SLAMF9	SYT7
MT1H	DCN
RP11−865I6.2	LTF
ESPN	IL20RA
FCGBP	IGF1
SIGLEC12	IBSP
NXPH4	TLL1
FAM163B	VCAN
PRIMA1	ELN
KCNG1	PTGER3
PEX5L	CHRDL2
ESM1	RIMBP2
ISL1	COL11A1
REN	WISP2
OCA2	SNCAIP
FGF23	COL17A1
TF	NGEF
MARCO	GAL
ZIC2	FGF10
ART4	CA12
HHATL	CAPN6
PDE4C	FBLN1
INHA	AMPH
TCN1	FAP
CD5L	ADCYAP1R1
LINC01447	RUNX1T1
IGF2BP1	PKP1
COL22A1	SERTAD4
SERPINA5	EPYC
CYSLTR2	PPEF1
NCAM1	ADAMTS2
CP	MMP2
TCERG1L	NID2
FIBCD1	CPXM1
RP11−298D21.1	TBX5
ALAS2	CCDC80
PAGE5	TBX15
RXFP1	MMP11
CA9	CTSG
LRP2	MMP9
SERPINA3	ISM1
HAMP	SYNDIG1
PGBD5	LIPG
DNAH11	MXRA5
RDH8	CHRDL1
NDRG1	SRPX2
IP6K3	FGF14
VEGFA	HTR2A
GRIN2B	MEDAG
CCL24	ZNF423
TOX3	CRISPLD2
RP11−320G24.1	PDGFRL
HBB	STMN2
FLRT3	HAS1
LINC00689	COMP
PLD5	PTN
CXCL5	WNT2
KCNQ2	SFRP4
CXCL8	MEOX2
SIGLEC11	RARRES2
CDH12	OGN
DPYSL5	ASPN
TNFRSF11B	ECM2
AK4	CXCL12
GP1BA	EBF3
PTGS2	COL1A1
S100A8	TSPAN11
SERPINB2	ACSS3
FGFBP2	COL12A1
FAM163A	SIM1
ZCCHC12	SMOC2
KEL	PRPH2
OR7C1	LOX
KRT8	THBS4
IL1R2	PDGFRB

GO:BP neg : 170 terms(FDR<0.01)

Term	FDR
multicellular organismal process	5.41e−09
single−multicellular organism process	7.52e−08
nervous system development	9.52e−07
ion transport	4.51e−06
system development	9.32e−06
chemotaxis	7.30e−05
taxis	7.30e−05
multicellular organism development	3.01e−04
anion transport	3.01e−04
response to external stimulus	3.76e−04
anatomical structure development	7.11e−04
axonogenesis	1.13e−03
single−organism process	1.13e−03
chemical homeostasis	1.29e−03
transmembrane transport	1.42e−03
cellular ion homeostasis	1.42e−03
developmental process	1.42e−03
neurogenesis	1.42e−03
axon guidance	1.50e−03
cellular chemical homeostasis	1.50e−03

GO:BP pos : 1037 terms(FDR<0.01)

Term	FDR
multicellular organismal process	1.67e−28
single−multicellular organism process	1.67e−28
extracellular matrix organization	1.67e−28
extracellular structure organization	1.67e−28
multicellular organism development	1.67e−28
single−organism developmental process	1.67e−28
anatomical structure development	1.67e−28
developmental process	1.67e−28
system development	1.67e−28
anatomical structure morphogenesis	3.31e−27
regulation of multicellular organismal p...	2.19e−26
animal organ development	2.51e−26
skeletal system development	3.12e−25
biological adhesion	4.51e−25
cell adhesion	1.80e−24
system process	4.70e−24
circulatory system development	6.63e−24
multicellular organismal macromolecule m...	1.42e−23
vasculature development	5.14e−23
collagen metabolic process	1.58e−22

GO:CC neg : 23 terms(FDR<0.01)

Term	FDR
intrinsic component of membrane	8.07e−09
integral component of membrane	2.20e−08
plasma membrane	5.50e−08
cell periphery	5.50e−08
extracellular space	8.07e−08
membrane part	8.25e−07
integral component of plasma membrane	8.90e−06
intrinsic component of plasma membrane	8.94e−06
plasma membrane part	5.91e−05
neuron part	2.02e−03

GO:CC pos : 78 terms(FDR<0.01)

Term	FDR
proteinaceous extracellular matrix	3.67e−29
extracellular matrix	3.67e−29
extracellular space	3.67e−29
extracellular region	3.67e−29
extracellular region part	3.67e−29
cell periphery	2.63e−25
plasma membrane	8.90e−25
intrinsic component of plasma membrane	1.01e−24
integral component of plasma membrane	3.67e−24
plasma membrane part	7.70e−24

GO:MF neg : 54 terms(FDR<0.01)

Term	FDR
transmembrane transporter activity	4.08e−05
substrate−specific transmembrane transpo...	4.08e−05
substrate−specific transporter activity	1.12e−04
transporter activity	1.40e−04
transmembrane receptor activity	1.42e−04
ion transmembrane transporter activity	1.42e−04
substrate−specific channel activity	1.42e−04
carbohydrate binding	1.42e−04
receptor activity	1.42e−04
molecular transducer activity	1.42e−04

GO:MF pos : 104 terms(FDR<0.01)

Term	FDR
receptor activity	4.08e−25
molecular transducer activity	4.08e−25
transmembrane receptor activity	5.01e−22
signaling receptor activity	1.82e−21
calcium ion binding	6.01e−21
transmembrane signaling receptor activit...	6.58e−21
glycosaminoglycan binding	9.66e−19
heparin binding	9.66e−19
sulfur compound binding	1.72e−16
extracellular matrix structural constitu...	1.12e−15

Cox regression:
logtest pv=3.3e−01
LHR=0.35 (CI = −0.35, 1.04)

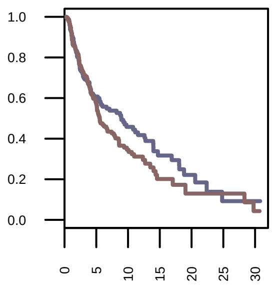

factor	p.value
Cluster	1.46e−02
Gender	2.72e−01
SampleType	4.35e−01
Dataset	9.88e−01

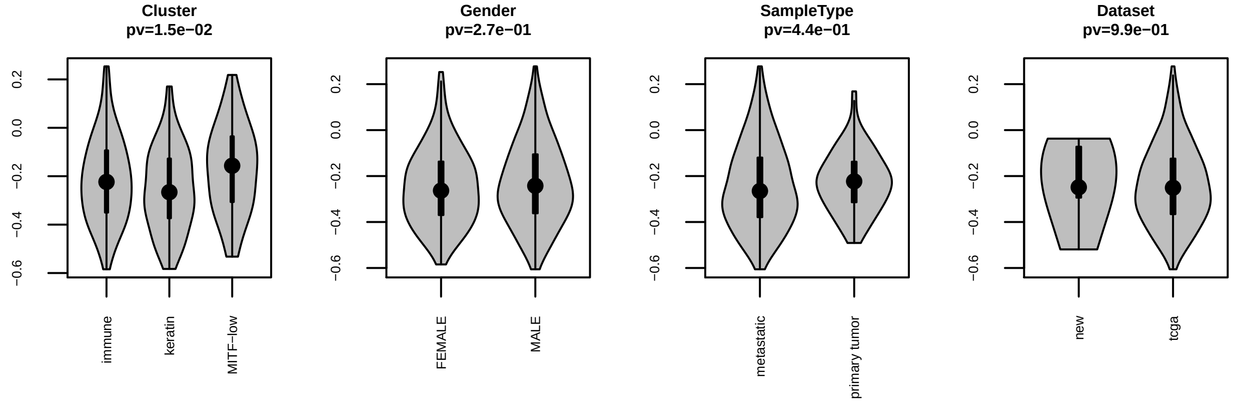

RIC16 (stability 0.495)

Metagene (involvement of features)

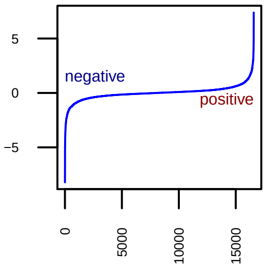

869 negative	780 positive
CHST9	WNT16
PMEL	ASB4
CDH1	TFAP2B
PKLR	ACPP
TRPM1	ZIC2
DCT	ADRB1
BCAN	MAGEC2
CST2	ARHGAP6
LGI3	TRHDE
FTLP14	ACTN2
RP11−429E11.2	COL19A1
RTN4RL1	ST8SIA5
FAM69C	PCYT1B
RP11−348B17.1	SRPX2
EPHA5	SFRP1
LINC01443	PTN
RDH8	NPTX2
LINC01531	MEOX2
TRIM63	SOD3
GJB2	COL9A1
MAGEL2	WISP3
SLC45A2	PCDHB2
MMP8	DLX2
LINC00282	MUC5B
SFRP5	GRIA2
CADPS	ITIH5
PRKG2	IL13RA2
AQP4	EDN3
FAM196B	OTOR
GPR143	MCHR1
ADCY2	HOXD11
SV2B	ANO3
FXYD3	DGKB
POU3F1	MYPN
ACSBG1	ABCA8
RP11−84D1.2	ITGA10
PASD1	TAGLN3
GAPDHS	HAPLN1
TSPAN10	GABRR1
CDH3	SYTL5
TMEM215	PPP1R1C
CRTAC1	GRIA4
MLPH	ABCA9
LIN28A	ABCA6
PRSS33	SLC24A2
RP11−615I2.2	ART3
RAP1GAP	LRRC52
CST5	SNED1
NRG3	XIRP2
LINC01317	SCRG1
MLANA	HNF4G
ALDH3B2	IL1RAPL1
TUNAR	NEGR1
MAL	PCSK1
TRIM58	MRGPRX4
CACNA1D	MRGPRX3
D4S234E	PCDH9
LRRTM1	SLITRK6
SFTPC	FGF7P2
ESRRG	AGMO
CHRNB2	RELN
TKTL1	MYBPC1
RAB3B	SLC30A10
SNCB	C6orf141
AC006262.5	RBM20
KIT	VIT
IRX6	MT1M
PLEKHA6	LINC00189
UG0898H09	AC012512.1
ABCB5	RP5−884M6.1
ILDR2	MEOX2−AS1
CA14	ERVMER61−1
ADAMTS16	LINC00326
MBP	C5orf58
ETNPPL	LINC01239
FAM167B	GAPDHP14
AFF3	RP11−408N14.1
RIPK4	RP11−280O1.2
GYG2	FCGR2C
CPEB1	RP11−13A1.1
NOV	CASC9
CNDP1	RP11−230G5.2

GO:BP neg : 80 terms(FDR<0.01)

Term	FDR
multicellular organismal process	1.43e−07
sensory perception	1.43e−07
system process	7.02e−07
neurological system process	2.38e−05
single−multicellular organism process	2.38e−05
anatomical structure development	2.38e−05
melanin biosynthetic process	4.30e−05
melanin metabolic process	7.14e−05
developmental pigmentation	8.85e−05
secondary metabolite biosynthetic proces...	1.04e−04
phenol−containing compound metabolic pro...	1.50e−04
single−organism developmental process	1.50e−04
system development	1.85e−04
developmental process	2.04e−04
multicellular organism development	2.11e−04
nervous system development	2.26e−04
pigmentation	9.73e−04
organic hydroxy compound metabolic proce...	1.17e−03
cell−cell signaling	1.19e−03
visual perception	1.20e−03

GO:BP pos : 249 terms(FDR<0.01)

Term	FDR
single−multicellular organism process	5.34e−14
multicellular organismal process	5.34e−14
nervous system development	3.16e−13
single−organism process	1.65e−12
developmental process	1.95e−11
anatomical structure development	3.76e−11
multicellular organism development	1.93e−10
single−organism developmental process	8.46e−10
system development	1.16e−09
chemical synaptic transmission	1.16e−09
anterograde trans−synaptic signaling	1.16e−09
synaptic signaling	1.16e−09
trans−synaptic signaling	1.16e−09
system process	1.61e−09
cell adhesion	3.51e−09
biological adhesion	4.23e−09
cell−cell signaling	1.06e−08
synapse organization	1.25e−08
cell communication	2.53e−08
single organism signaling	8.27e−08

GO:CC neg : 31 terms(FDR<0.01)

Term	FDR
plasma membrane	5.68e−11
cell periphery	8.34e−11
plasma membrane part	2.32e−10
plasma membrane region	1.92e−08
intrinsic component of membrane	3.45e−08
membrane part	1.47e−07
integral component of membrane	2.09e−07
melanosome membrane	8.35e−07
chitosome	8.35e−07
extracellular space	1.34e−03

GO:CC pos : 48 terms(FDR<0.01)

Term	FDR
plasma membrane	8.62e−16
cell periphery	8.62e−16
integral component of plasma membrane	1.47e−15
intrinsic component of plasma membrane	8.25e−15
extracellular space	9.16e−12
intrinsic component of membrane	9.16e−12
plasma membrane part	2.23e−11
integral component of membrane	7.79e−11
membrane part	2.24e−08
proteinaceous extracellular matrix	4.22e−07

GO:MF neg : 26 terms(FDR<0.01)

Term	FDR
transmembrane transporter activity	5.15e−04
ion transmembrane transporter activity	6.30e−04
channel activity	6.30e−04
passive transmembrane transporter activi...	6.30e−04
transporter activity	6.30e−04
substrate−specific transmembrane transpo...	6.30e−04
calcium ion binding	6.75e−04
cation transmembrane transporter activit...	8.59e−04
substrate−specific channel activity	1.43e−03
ion channel activity	2.40e−03

GO:MF pos : 66 terms(FDR<0.01)

Term	FDR
receptor activity	1.98e−06
molecular transducer activity	1.98e−06
signaling receptor activity	1.98e−06
transmembrane signaling receptor activit...	1.98e−06
transmembrane receptor activity	1.98e−06
ion channel activity	2.43e−06
substrate−specific channel activity	3.25e−06
calcium ion binding	1.18e−05
channel activity	1.41e−05
passive transmembrane transporter activi...	1.41e−05

Cox regression:
logtest pv=5.1e−04
LHR=−1.60 (CI = −2.53, −0.66)

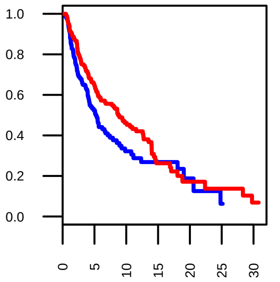

factor	p.value
Cluster	7.75e−35
SampleType	1.12e−05
Gender	6.39e−01
Dataset	6.76e−01

Cluster
pv=7.7e−35

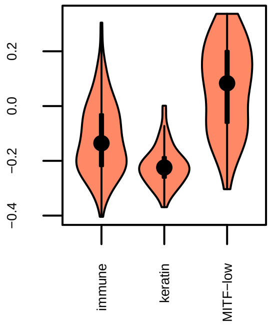

SampleType
pv=1.1e−05

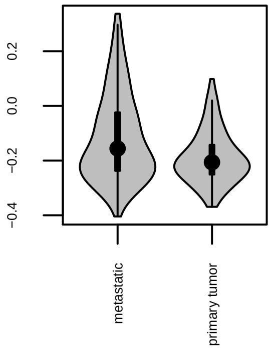

Gender
pv=6.4e−01

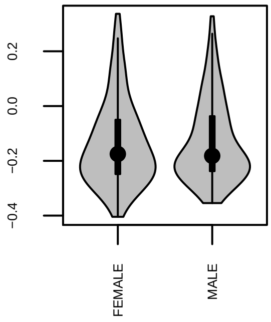

Dataset
pv=6.8e−01

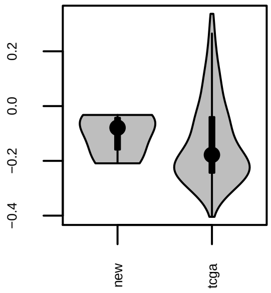

RIC19 (stability 0.924)

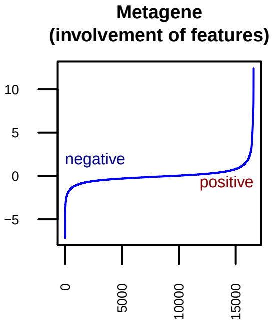

459 negative	801 positive
SPRR3	KRT33A
RP11−63E9.1	TFAP2B
BAAT	LTF
SPRR2A	BARX2
RP11−369C8.1	GABRP
VGF	KRT31
SPINK6	SEZ6L
LOXL4	PNPLA5
TMEM26	CPNE6
DEFB4A	FA2H
S100A12	TOX3
CBLN4	KRT32
COL11A2	SULT1E1
NMRK2	SCGB2A2
SFTPA1	GPRC5D
SULT1C2	SLC27A6
NPTX1	SLC9A2
OGDHL	PRB2
AL035610.1	SPDEF
PTPRD	STATH
FSTL5	GFAP
LINC00473	CA6
ONECUT2	KRT34
C10orf99	KRT33B
NEFL	PPP1R1B
S100A9	GPR12
PPP1R3C	HMGCS2
TMPRSS11D	SAA2
SYNPR	CLDN10
HTR3A	MRAP2
SNTG1	KRT85
IGFL1	KRT7
RP11−488I20.8	PIK3C2G
EPGN	LGR5
SLC35F1	KRT71
AC110781.3	STAC2
IGF2	SLC25A48
CYTL1	AWAT2
CCNYL2	TRIM55
CXCL17	THRSP
FMO3	ADCY8
S100A7A	WIF1
CFI	SLC34A2
EPYC	TCHH
SFTPA2	PIP
STK32B	DCD
DMGDH	KRT82
RP1−47M23.3	ZG16B
FRG2DP	PM20D1
DUOXA1	CLDN19
BHMT2	PI16
RASL10B	TRPV6
RHCG	CLDN3
PPEF1	MOGAT2
RP11−218E20.3	LMO1
UNC80	KLK1
SERPINB4	CTNND2
LINC00654	CST1
PITX1	KRT86
FLJ16779	KRT74
IGKV5−2	KRT83
HOXA10	KRT19
RP11−706O15.3	KRT15
IGHD	KRTAP4−4
IL6	KRT27
S100A8	MUCL1
COL20A1	ARPP21
FUT3	SAA1
SPINK7	KRT28
EPDR1	DSG4
HOXA7	DES
LRRC4	CALML5
SCML4	PSAPL1
MMP13	C5orf46
LINC01314	CCDC129
PKHD1	KRTAP9−7
NLGN4X	KRTAP11−1
AC092652.1	TCHHL1
FOS	KRTAP8−1
MTRNR2L1	B3GALT5
KRT13	SPRR4
SFTPB	DGAT2L6

GO:BP neg : 87 terms(FDR<0.01)

Term	FDR
multicellular organismal process	2.56e−07
single−multicellular organism process	7.52e−07
cornification	1.40e−06
tissue development	1.20e−05
response to corticosteroid	1.01e−04
keratinization	1.01e−04
anatomical structure development	1.01e−04
negative regulation of endopeptidase act...	1.01e−04
system development	1.01e−04
response to glucocorticoid	1.01e−04
negative regulation of peptidase activit...	1.27e−04
regulation of endopeptidase activity	1.39e−04
multicellular organism development	1.39e−04
keratinocyte differentiation	1.40e−04
epithelium development	1.70e−04
inflammatory response	3.10e−04
regulation of peptidase activity	3.76e−04
peptide cross−linking	3.76e−04
single−organism process	3.76e−04
epidermal cell differentiation	3.76e−04

GO:BP pos : 51 terms(FDR<0.01)

Term	FDR
keratinization	1.88e−28
keratinocyte differentiation	1.88e−28
epidermis development	1.88e−28
skin development	1.88e−28
epidermal cell differentiation	1.88e−28
epithelial cell differentiation	1.88e−28
epithelium development	1.88e−28
tissue development	1.88e−28
cornification	1.84e−26
multicellular organismal process	7.97e−24
cell differentiation	1.64e−18
single−multicellular organism process	1.88e−18
animal organ development	3.12e−18
system development	5.26e−18
cellular developmental process	7.72e−16
anatomical structure development	3.20e−15
multicellular organism development	4.60e−15
developmental process	2.84e−13
single−organism developmental process	5.38e−13
molting cycle	3.01e−10

GO:CC neg : 10 terms(FDR<0.01)

Term	FDR
extracellular space	1.37e−18
extracellular region	3.67e−11
extracellular region part	2.75e−09
extracellular matrix	1.83e−08
cornified envelope	6.42e−07
proteinaceous extracellular matrix	6.42e−07
cell periphery	7.33e−05
plasma membrane	8.48e−05
intrinsic component of membrane	1.98e−02
collagen trimer	3.85e−02

GO:CC pos : 27 terms(FDR<0.01)

Term	FDR
intermediate filament	6.11e−29
keratin filament	6.11e−29
intermediate filament cytoskeleton	6.11e−29
polymeric cytoskeletal fiber	8.25e−27
supramolecular fiber	2.16e−25
supramolecular polymer	3.93e−25
supramolecular complex	3.93e−25
extracellular space	1.05e−16
extracellular region	2.65e−16
extracellular region part	4.40e−08

GO:MF neg : 15 terms(FDR<0.01)

Term	FDR
serine−type endopeptidase inhibitor acti...	1.93e−05
endopeptidase inhibitor activity	1.93e−05
peptidase inhibitor activity	2.04e−05
endopeptidase regulator activity	2.04e−05
serine−type endopeptidase activity	2.83e−05
receptor binding	7.16e−05
peptidase regulator activity	7.36e−05
serine−type peptidase activity	1.40e−04
serine hydrolase activity	1.67e−04
growth factor activity	4.29e−04

GO:MF pos : 17 terms(FDR<0.01)

Term	FDR
structural molecule activity	1.12e−05
peptidase inhibitor activity	1.16e−03
endopeptidase inhibitor activity	8.44e−03
endopeptidase regulator activity	1.03e−02
calcium ion binding	1.03e−02
peptidase regulator activity	1.93e−02
enzyme inhibitor activity	2.27e−02
neuropeptide receptor binding	2.90e−02
hormone activity	3.24e−02
structural constituent of epidermis	4.04e−02

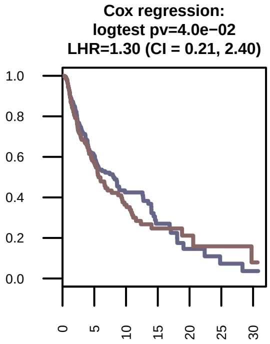

factor	p.value
Dataset	2.47e−10
Cluster	8.19e−07
SampleType	1.57e−06
Gender	8.07e−01

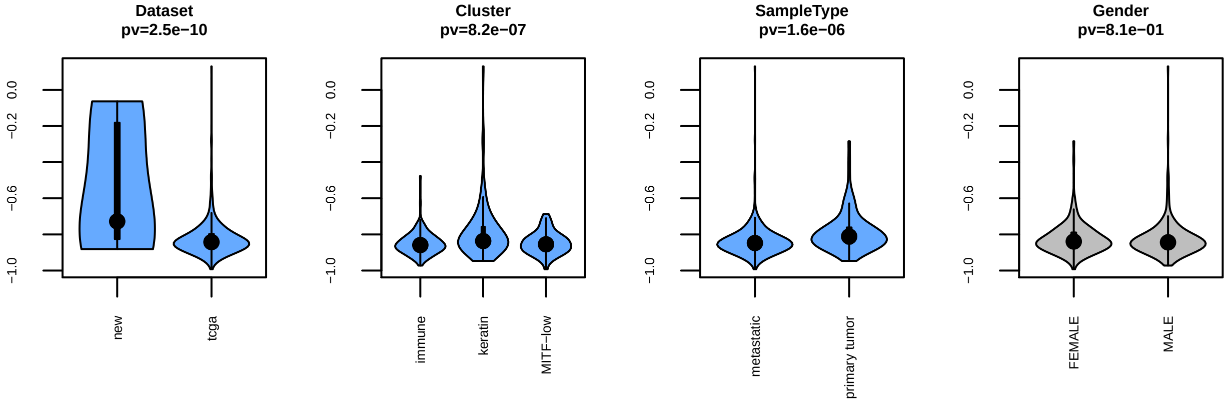

RIC25 (stability 0.993)

Metagene (involvement of features)

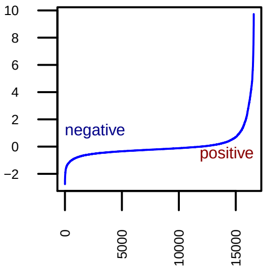

373 negative	1466 positive
KLHL14	FGR
CSF3	CD38
CXCL5	ITGAL
ADRA1B	TTC22
CNTN1	CEACAM21
IGKV1D−39	IL32
IGKV1D−33	TRAF3IP3
PRSS50	ETV7
GABRB2	CD4
IGHV7−81	TYROBP
IGKV1−33	ALOX5
LY6K	CD6
IGKV1OR2−108	WAS
IGHV3−43	MARCO
FABP4	CD74
CDH15	BIRC3
IGHJ2	TYMP
NOG	SLAMF7
IGKV1D−27	SH2D2A
LINC00282	TNFRSF1B
PRSS35	MSR1
HOXC12	ADAM28
IGHV1OR15−9	BARX2
RP11−488I20.8	LCP2
THBS4	TNFRSF9
AC108142.1	TNIP3
COCH	LY75
MAGEC1	PARP12
PRPH	PRKCQ
DCX	CD84
ATP12A	SPI1
SCN7A	DAPP1
USH2A	ACAP1
CASQ2	CD5L
COLCA1	TBX21
IGKV2OR22−4	IPCEF1
RGS5	ICAM3
HOOK1	APBB1IP
BMX	CST7
DLX5	P2RY10
SERPINA9	SP140
TPSD1	PTPRC
OCLN	LRP2
GRIN2A	FYB
TSPEAR	FCN1
ABCA4	PILRA
ZNF215	CASS4
CYTL1	SIGLEC1
IGLV3−16	SIRPG
TSPEAR−AS1	OAS1
COL14A1	LAG3
RIMS2	LYZ
C20orf166−AS1	CD209
IGHV1OR15−2	CECR1
SHISA3	TREM2
SLC38A11	IL12RB1
AKR1C1	CYTH4
CTTNBP2	LGALS2
PTPRN	APOL4
TMEM132D	APOL1
RP11−284F21.10	NCF4
ADCYAP1R1	CSF2RB
IGKV1−39	IL2RB
SULT1C4	GZMH
PMP2	GZMB
GDF7	SLA2
DENND2A	SIRPB1
IGKV1D−43	HCK
IGKV1OR2−6	SAMHD1
SMCO3	PPP1R16B
CHRDL2	TLR8
REN	CD40LG
RP5−1198O20.4	TNFSF13B
DNAJA4	IRG1
FRZB	CORO1A
RP11−284F21.9	IL21R
PDLIM3	CD37
LINC00707	IL4I1
TENM3	LILRB1
IGHE	LILRA1
CTSG	RASAL3
NGF	EBI3

GO:BP neg : 60 terms(FDR<0.01)

Term	FDR
multicellular organismal process	6.32e−08
anatomical structure development	1.20e−07
multicellular organism development	1.20e−07
system development	1.20e−07
single−multicellular organism process	1.26e−07
single−organism developmental process	1.35e−06
cell differentiation	2.79e−06
developmental process	3.38e−06
cellular developmental process	1.67e−05
animal organ development	1.14e−04
cell−cell signaling	5.33e−04
tissue development	2.38e−03
positive regulation of nervous system de...	6.01e−03
response to jasmonic acid	6.01e−03
cellular response to jasmonic acid stimu...	6.01e−03
regulation of hormone levels	9.02e−03
nervous system development	1.24e−02
generation of neurons	1.92e−02
neurogenesis	2.14e−02
regulation of multicellular organismal p...	2.86e−02

GO:BP pos : 1358 terms(FDR<0.01)

Term	FDR
immune response	1.71e−29
immune system process	1.71e−29
defense response	1.71e−29
regulation of immune system process	1.71e−29
innate immune response	1.71e−29
leukocyte activation	1.71e−29
regulation of immune response	1.71e−29
cell activation	1.71e−29
positive regulation of immune system pro...	1.71e−29
lymphocyte activation	1.71e−29
immune effector process	1.71e−29
adaptive immune response	1.71e−29
T cell activation	1.71e−29
positive regulation of immune response	1.71e−29
cytokine−mediated signaling pathway	1.71e−29
regulation of leukocyte activation	1.71e−29
inflammatory response	1.71e−29
regulation of cell activation	1.71e−29
cellular response to cytokine stimulus	1.71e−29
response to other organism	1.71e−29

GO:CC neg : 13 terms(FDR<0.01)

Term	FDR
extracellular space	2.38e−11
extracellular region	2.93e−09
extracellular region part	2.38e−07
proteinaceous extracellular matrix	1.80e−04
extracellular matrix	1.80e−04
integral component of plasma membrane	1.56e−02
plasma membrane part	1.86e−02
intrinsic component of plasma membrane	2.75e−02
ion channel complex	3.12e−02
transmembrane transporter complex	3.12e−02

GO:CC pos : 99 terms(FDR<0.01)

Term	FDR
cell periphery	2.04e−29
plasma membrane	2.04e−29
plasma membrane part	2.04e−29
intrinsic component of membrane	2.04e−29
intrinsic component of plasma membrane	2.04e−29
integral component of plasma membrane	2.04e−29
integral component of membrane	2.04e−29
membrane part	2.04e−29
side of membrane	2.04e−29
external side of plasma membrane	2.20e−27

GO:MF neg : 6 terms(FDR<0.01)

Term	FDR
growth factor activity	7.30e−06
cytokine activity	4.64e−03
phenanthrene 9,10−monooxygenase activity	4.64e−03
ketosteroid monooxygenase activity	4.64e−03
trans−1,2−dihydrobenzene−1,2−diol dehydr...	4.64e−03
receptor binding	1.15e−02

GO:MF pos : 117 terms(FDR<0.01)

Term	FDR
receptor activity	7.16e−29
molecular transducer activity	7.16e−29
transmembrane receptor activity	7.16e−29
transmembrane signaling receptor activit...	7.16e−29
signaling receptor activity	7.16e−29
signal transducer activity	7.16e−29
antigen binding	7.36e−19
cytokine receptor activity	2.31e−14
cytokine activity	1.86e−12
peptide antigen binding	8.16e−12

Cox regression: logtest pv=2.8e−07 LHR=−1.13 (CI = −1.57, −0.70)

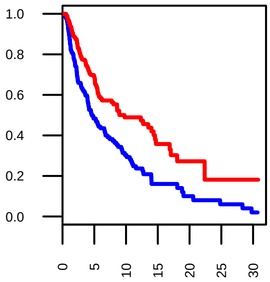

factor	p.value
Cluster	9.33e−53
SampleType	4.37e−05
Dataset	2.24e−03
Gender	9.44e−02

Cluster pv=9.3e−53

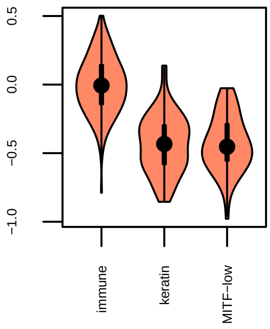

SampleType pv=4.4e−05

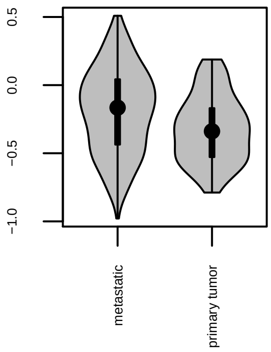

Dataset pv=2.2e−03

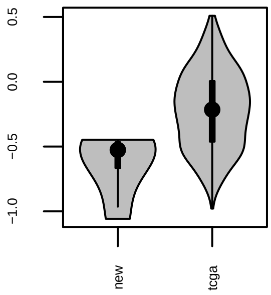

Gender pv=9.4e−02

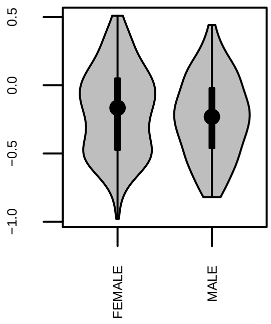

RIC27 (stability 0.929)

Metagene (involvement of features)

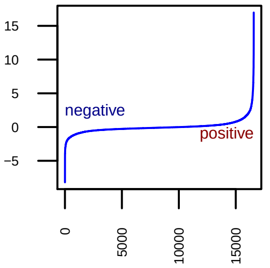

714 negative	1177 positive
COL11A1	MEOX1
MMP13	CD79B
PRKG2	CD22
EPYC	LTF
HAPLN1	STAP1
MXRA5Y	CP
AMER2	ELN
WISP2	NPFFR2
IBSP	PKP2
GJB2	LAMC2
CYP11A1	SNCAIP
IRG1	RASGRP2
SIX2	CAPN6
EGFL6	CDH17
GSTM1	COL4A4
SOX1	TCF7
MT1H	COL19A1
COL6A6	P2RX5
SDC1	SLCO1A2
WNT2	MYBPC2
MMP3	ACHE
TNFAIP6	FMO2
IFNG	GALNT16
SFRP2	TCL1A
CA12	SYNDIG1
MEGF10	VSIG1
GLDN	CHRDL1
DIO2	CD40LG
SPATA18	CCL22
WT1	FOXF1
WNT7B	FCER2
RP11−738O11.13	CLEC4M
GZMH	SHD
GBP1P1	CD79A
AQP9	GRIK5
MFAP2	VIPR2
DCC	CCL24
DHRS2	MEOX2
GBP7	PTGDS
GPR84	SLC1A2
ITGBL1	ART4
VSNL1	AICDA
ACOXL	TREML2
PTPRD	C7
PTK6	LIFR
WBSCR17	VIPR1
CTAG2	CD207
WT1−AS	TNR
VSIG4	PLA2G2D
CNTN6	CR2
CDCP1	LRMP
WFDC2	BCL11A
PAX1	TNFSF11
WIF1	CLU
IGHG1	DPPA4
EDIL3	PAEP
CCL8	ITIH5
KCNA2	CHST8
CXCL3	POF1B
APLN	PACSIN1
MXRA5	C3
TIMP4	CCR7
KLK10	RGS13
MME	TSPAN8
F7	VPREB3
NRG3	TBC1D27
ROR2	LBP
ZIC1	TNNT3
ANO5	NAPSB
MUM1L1	RAI2
HTRA3	FCRL2
FAM19A5	ALDH3B2
SIM1	NTS
MEG3	TTC9
ALX4	DSC1
GLRB	ADAMTS8
FREM1	DTX1
XG	TESPA1
CMTM5	EDAR
CCL4L1	STAB2
IGKC	BLK
NALCN	IL33

GO:BP neg : 234 terms(FDR<0.01)

Term	FDR
multicellular organismal process	7.52e−14
single−multicellular organism process	1.65e−12
multicellular organism development	4.76e−10
chemokine−mediated signaling pathway	1.05e−09
anatomical structure development	1.11e−09
defense response	1.78e−09
single−organism developmental process	3.01e−09
developmental process	3.76e−09
system development	5.85e−09
single−organism process	2.11e−08
neutrophil chemotaxis	2.73e−08
neutrophil migration	1.25e−07
leukocyte chemotaxis	1.27e−07
response to external stimulus	2.47e−07
cell chemotaxis	2.51e−07
chemotaxis	4.04e−07
taxis	4.07e−07
immune response	4.84e−07
inflammatory response	6.01e−07
granulocyte chemotaxis	1.20e−06

GO:BP pos : 577 terms(FDR<0.01)

Term	FDR
signaling	9.02e−16
single organism signaling	9.02e−16
multicellular organismal process	1.09e−15
response to stimulus	1.09e−15
cell communication	4.51e−15
single−multicellular organism process	4.51e−15
lymphocyte activation	6.44e−14
G−protein coupled receptor signaling pat...	4.34e−13
single−organism process	4.34e−13
adaptive immune response	4.81e−13
system process	6.15e−13
biological adhesion	5.39e−12
signal transduction	6.36e−12
cell adhesion	7.09e−12
immune system process	1.70e−11
lymphocyte differentiation	1.97e−11
immune response	2.65e−11
cell−cell adhesion	4.34e−11
regulation of multicellular organismal p...	9.50e−11
leukocyte proliferation	4.29e−10

GO:CC neg : 49 terms(FDR<0.01)

Term	FDR
extracellular space	6.78e−14
extracellular region	1.10e−12
intrinsic component of membrane	3.67e−11
proteinaceous extracellular matrix	1.05e−10
plasma membrane part	2.24e−10
intrinsic component of plasma membrane	2.29e−10
collagen trimer	2.29e−10
integral component of plasma membrane	2.29e−10
cell periphery	4.48e−10
plasma membrane	6.97e−10

GO:CC pos : 46 terms(FDR<0.01)

Term	FDR
cell periphery	3.06e−29
plasma membrane	3.06e−29
plasma membrane part	3.06e−29
intrinsic component of membrane	3.06e−29
membrane part	3.06e−29
integral component of membrane	3.06e−29
intrinsic component of plasma membrane	3.21e−27
integral component of plasma membrane	3.21e−27
extracellular space	7.54e−17
extracellular region	3.30e−13

GO:MF neg : 55 terms(FDR<0.01)

Term	FDR
chemokine activity	7.30e−11
chemokine receptor binding	6.87e−09
cytokine activity	6.87e−09
serine−type peptidase activity	7.84e−07
serine hydrolase activity	9.45e−07
receptor binding	1.22e−06
cytokine receptor binding	2.39e−06
serine−type endopeptidase activity	2.74e−06
CXCR chemokine receptor binding	3.90e−06
receptor activity	3.90e−06

GO:MF pos : 97 terms(FDR<0.01)

Term	FDR
receptor activity	8.59e−29
molecular transducer activity	8.59e−29
signaling receptor activity	8.59e−29
transmembrane signaling receptor activit...	8.59e−29
transmembrane receptor activity	8.59e−29
signal transducer activity	2.22e−22
G−protein coupled receptor activity	1.59e−12
substrate−specific channel activity	2.52e−08
channel activity	3.18e−08
passive transmembrane transporter activi...	3.18e−08

Cox regression:
logtest pv=6.2e−02
LHR=−0.76 (CI = −1.59, 0.06)

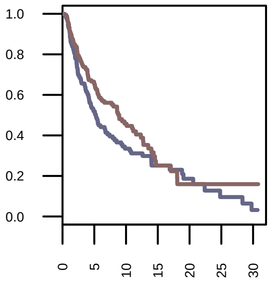

factor	p.value
Cluster	2.12e−20
SampleType	5.53e−07
Dataset	5.38e−01
Gender	9.41e−01

Cluster
pv=2.1e−20

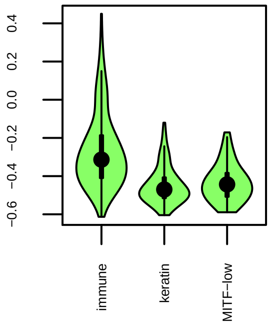

SampleType
pv=5.5e−07

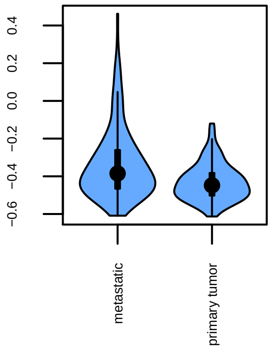

Dataset
pv=5.4e−01

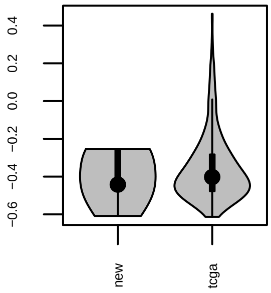

Gender
pv=9.4e−01

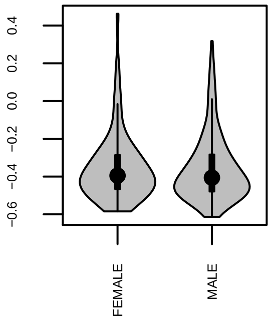

RIC28 (stability 0.952)

Metagene (involvement of features)

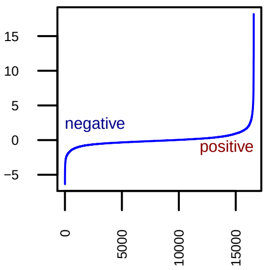

393 negative	790 positive
KRT13	SELE
KRT4	GPRC5A
SIGLEC8	SLC11A1
C7	MARCO
SYT6	BIRC3
NRG3	PTPRN
RP6−24A23.7	CHRDL2
CXCR2P1	LAMC2
CDH7	PAGE1
CRNN	PTGS2
ZIC1	CA12
BTNL9	CXCL2
FLG2	IL11
SCGB3A1	OSM
SFRP4	CCL22
PSG4	AQP9
ZIC4	HAS1
RP6−24A23.3	TFPI2
IRS4	SERPINE1
LCE1A	TYRP1
TUBA3C	DKK1
KCNJ5	CSF3
TRIM67	CCL2
MSMB	CCL8
ALK	ODAM
ELFN1−AS1	AREG
SLC7A4	WNT5A
SEC14L6	IL1A
LHX2	CCL20
LPPR5	MMP8
SLC40A1	G0S2
ELFN2	PI3
GJA3	TREM1
PON3	CXCL6
DNASE2B	EREG
KRT1	IL1B
LINC01419	FFAR2
GSG1L	LIF
DGKI	APOBEC3A
KLK2	VGF
CUX2	LBP
PCSK2	CHI3L1
CLCA4	SAA2
NOVA1	SLC19A3
TMEM132E	IL6
LINC01579	IL1RN
AC012512.1	IER3
FMO2	CH25H
LRRC4B	MYPN
PLA2G4F	GPR84
CHIT1	CYR61
MUC21	S100A8
PODN	NFKBIZ
AC108142.1	LCN2
GRIK2	PLEKHS1
KLK4	MMP3
BPIFB2	TDRD9
LCE1B	KCNJ15
EBF2	CD1A
SLC30A10	SLAMF9
AC145110.1	FCGR3B
SLC5A4	IL24
PLA2G2D	S100A9
SPRR3	S100A12
LINC01021	IGFN1
OMD	PTX3
CBLN4	CXCL3
PHF21B	CXCL5
GRIK3	CXCL1
WT1	S100P
HCN1	MMP10
CDKN2A	NNMT
CHST8	C15orf48
CYP4B1	GREM1
RP11−94H18.1	ANGPTL4
DIRAS2	CXCL8
PPM1E	FPR2
LAMP5	LRG1
ITGAD	SOSTDC1
ST8SIA2	KCNK3
FAT3	CCL11
FRAS1	SAA1

GO:BP neg : 29 terms(FDR<0.01)

Term	FDR
peptide cross−linking	4.21e−08
skin development	3.11e−04
regulation of postsynaptic membrane pote...	3.11e−04
epidermal cell differentiation	5.41e−04
keratinization	5.41e−04
epidermis development	8.52e−04
multicellular organismal process	9.88e−04
multicellular organism development	1.39e−03
excitatory postsynaptic potential	1.40e−03
keratinocyte differentiation	1.65e−03
single−multicellular organism process	1.88e−03
anatomical structure development	1.88e−03
chemical synaptic transmission, postsyna...	3.47e−03
system development	3.54e−03
neurological system process	4.01e−03
developmental process	4.13e−03
single−organism developmental process	7.08e−03
nervous system development	1.50e−02
behavior	2.85e−02
cornification	3.76e−02

GO:BP pos : 1021 terms(FDR<0.01)

Term	FDR
inflammatory response	5.01e−28
defense response	5.01e−28
response to external stimulus	5.01e−28
response to stimulus	7.89e−24
immune response	9.52e−24
immune system process	9.52e−24
response to chemical	2.36e−21
leukocyte migration	1.26e−20
response to bacterium	2.17e−20
multicellular organismal process	4.06e−20
response to cytokine	1.08e−18
response to lipopolysaccharide	9.27e−18
cell migration	1.05e−17
cell motility	1.32e−17
localization of cell	1.32e−17
response to molecule of bacterial origin	1.32e−17
locomotion	4.09e−17
response to other organism	4.09e−17
response to external biotic stimulus	4.27e−17
cell chemotaxis	7.52e−17

GO:CC neg : 6 terms(FDR<0.01)

Term	FDR
cornified envelope	9.16e−06
extracellular space	2.93e−04
intrinsic component of membrane	8.71e−03
extracellular region	8.71e−03
proteinaceous extracellular matrix	2.97e−02
integral component of membrane	4.89e−02

GO:CC pos : 48 terms(FDR<0.01)

Term	FDR
extracellular space	1.83e−28
extracellular region	8.52e−24
extracellular region part	5.07e−16
plasma membrane part	5.50e−13
plasma membrane	1.28e−12
cell periphery	1.65e−12
intrinsic component of plasma membrane	6.81e−12
integral component of plasma membrane	2.98e−11
proteinaceous extracellular matrix	4.48e−11
extracellular matrix	1.67e−10

GO:MF neg : 0 terms(FDR<0.01)

GO:MF pos : 64 terms(FDR<0.01)

Term	FDR
cytokine activity	3.65e−23
receptor binding	3.01e−18
receptor activity	7.94e−14
molecular transducer activity	7.94e−14
growth factor activity	1.29e−13
cytokine receptor binding	4.94e−13
chemokine activity	9.20e−11
chemokine receptor binding	1.02e−09
G−protein coupled receptor binding	1.38e−09
signaling receptor activity	1.50e−09

Cox regression: logtest pv=1.9e−01 LHR=−0.74 (CI = −1.84, 0.37)

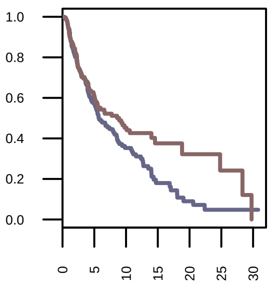

factor	p.value
SampleType	1.61e−06
Gender	2.43e−01
Dataset	6.16e−01
Cluster	6.90e−01

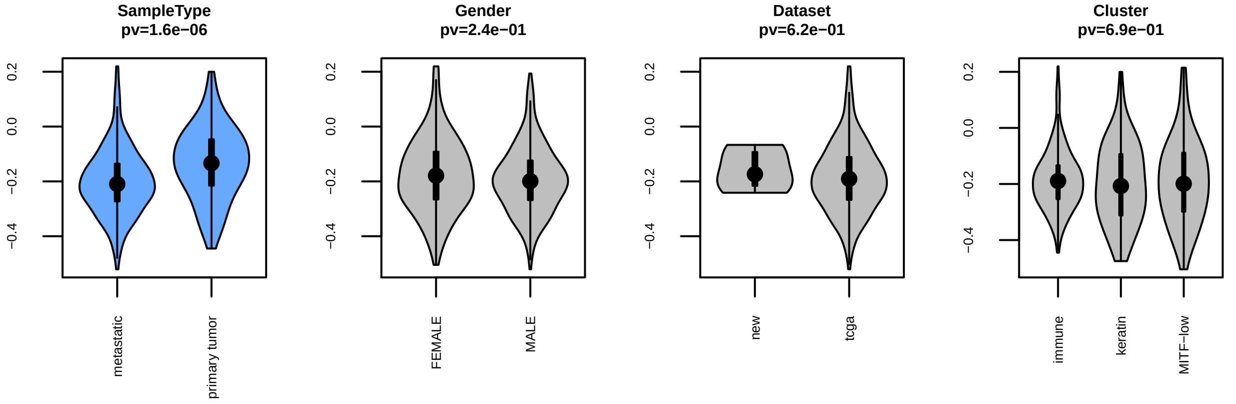

RIC31 (stability 0.913)

Metagene (involvement of features)

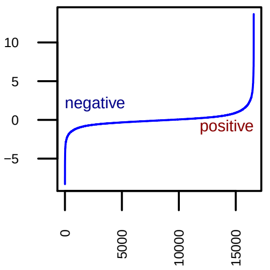

397 negative	772 positive
SPRR3	TNMD
OLIG2	PRSS22
CRNN	DNAH5
KRT13	ROS1
SPRR2A	PRSS8
KRT6A	CAPN6
KRT6C	GRHL2
NR5A1	CEACAM6
MAT1A	PGC
SPRR2D	MSLN
TLX1	ESRP2
SPRR2E	AGR2
CNTNAP5	FOLR1
A2ML1	SCNN1A
KRT16	ITGB6
LBP	EPCAM
KIF25−AS1	SFTPA1
CLCA4	C4BPA
SPRR1B	SLPI
PRKG2	ATP13A4
CMTM5	AP1M2
COL20A1	CRB3
SPRR1A	NAPSA
CKM	RAB25
RP11−513O17.2	VSTM2L
JPH4	SFTPD
UG0898H09	KRT7
KRT14	NKX2−1
DSG3	MMP7
KRT4	ANXA3
CHL1−AS2	CYP4B1
LINC01198	MAGEA4
FABP7	SCGB1A1
SV2A	TMEM132D
LINC01235	SLC34A2
PTPRZ1	PAGE5
ZNF648	LAD1
SLC30A3	SCGB3A1
ITPRIPL1	AQP5
CCDC26	CCDC64B
CD5L	PDZK1IP1
SLC38A8	PIGR
TMPRSS11A	ELF3
DDX25	SCGB3A2
LY6D	GRIK2
RP3−323A16.1	HCN1
FXYD7	CHMP4C
SLCO1A2	CLDN3
LINC01502	SCNN1G
SPESP1	ELFN2
SPATA31C2	KLK11
TSPEAR−AS1	SCNN1B
NTS	SFTPC
ADAMTS4	FSTL5
KCNJ10	SFTPB
MOG	IRX2
NKX2−2	SDR16C5
CALML3	SOSTDC1
PAGE1	KNDC1
RP1−40E16.9	AQP4
KRT78	SLC26A9
SBSN	CD164L2
OLIG1	CST6
F5	KDF1
BMX	MAPK15
MYH7	C1orf116
GRIN2B	SLITRK6
KRT5	SFTPA2
LIN28B	C5orf38
LINC01317	DMBT1
TRIM58	CLDN4
RP11−476K15.1	CXCL17
SPRR2F	CTSE
FAM101A	KRT16P2
MCHR1	SFTA3
HIST1H2AG	CYP2B7P
DPY19L2	SLC22A31
JAKMIP2	RP11−488I20.9
SPDYC	SLC6A14
CLGN	RAB27B
CPB1	CLDN18
FAM198A	TSPAN1

GO:BP neg : 29 terms(FDR<0.01)

Term	FDR
cornification	4.06e−15
keratinization	1.43e−10
keratinocyte differentiation	4.71e−09
multicellular organismal process	2.22e−08
epidermal cell differentiation	1.14e−07
single−multicellular organism process	3.76e−07
skin development	7.71e−07
multicellular organism development	7.71e−07
system development	1.10e−06
epidermis development	4.96e−06
anatomical structure development	9.90e−06
peptide cross−linking	9.90e−06
single−organism developmental process	6.36e−05
developmental process	1.00e−04
cell differentiation	1.00e−04
animal organ development	2.73e−04
epithelial cell differentiation	3.27e−04
cellular developmental process	6.52e−04
tissue development	3.17e−03
single−organism cellular process	5.08e−03

GO:CC neg : 10 terms(FDR<0.01)

Term	FDR
cornified envelope	2.02e−09
extracellular region	1.01e−05
cell periphery	1.04e−03
extracellular region part	2.02e−03
extracellular space	3.15e−03
plasma membrane	4.58e−03
integral component of plasma membrane	1.18e−02
plasma membrane part	2.44e−02
intrinsic component of plasma membrane	2.44e−02
keratin filament	4.95e−02

GO:MF neg : 1 terms(FDR<0.01)

Term	FDR
structural molecule activity	2.36e−02

GO:BP pos : 193 terms(FDR<0.01)

Term	FDR
epithelial cell differentiation	1.28e−17
epidermis development	2.00e−15
epithelium development	2.00e−15
skin development	5.64e−15
tissue development	6.32e−15
multicellular organismal process	1.15e−11
keratinocyte differentiation	9.02e−11
single−multicellular organism process	1.42e−10
keratinization	1.42e−10
epidermal cell differentiation	1.80e−10
animal organ development	5.06e−09
cornification	3.63e−08
anatomical structure development	5.78e−08
multicellular organism development	1.07e−07
system development	1.60e−07
peptide cross−linking	3.20e−07
pattern specification process	7.43e−07
single−organism developmental process	1.11e−06
single−organism process	1.11e−06
developmental process	1.58e−06

GO:CC pos : 82 terms(FDR<0.01)

Term	FDR
extracellular region	1.50e−16
cell periphery	1.47e−15
plasma membrane	1.10e−14
extracellular space	2.84e−14
plasma membrane part	6.60e−12
cornified envelope	1.74e−10
extracellular region part	2.07e−10
intrinsic component of plasma membrane	4.35e−10
integral component of plasma membrane	1.96e−09
intrinsic component of membrane	3.48e−09

GO:MF pos : 40 terms(FDR<0.01)

Term	FDR
receptor activity	1.87e−08
molecular transducer activity	1.87e−08
transmembrane receptor activity	1.72e−07
transmembrane signaling receptor activit...	2.79e−05
signaling receptor activity	5.41e−05
endopeptidase inhibitor activity	3.72e−04
peptidase inhibitor activity	5.40e−04
endopeptidase regulator activity	5.90e−04
G−protein coupled receptor activity	6.68e−04
structural molecule activity	1.42e−03

Cox regression: logtest pv=2.2e−02 LHR=1.27 (CI = 0.26, 2.28)

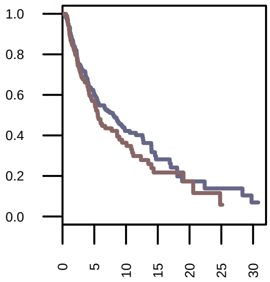

factor	p.value
SampleType	6.15e−18
Cluster	3.08e−17
Dataset	2.44e−04
Gender	2.28e−01

SampleType pv=6.1e−18

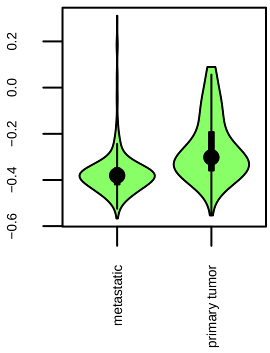

Cluster pv=3.1e−17

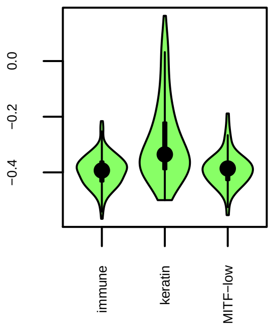

Dataset pv=2.4e−04

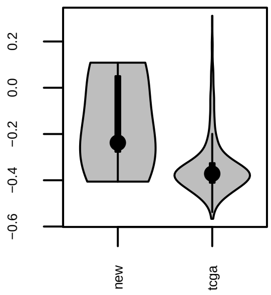

Gender pv=2.3e−01

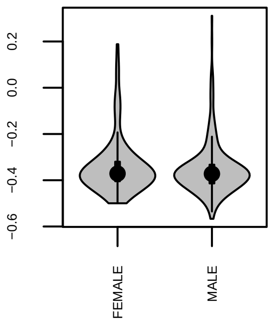

RIC37 (stability 0.760)

Metagene
(involvement of features)

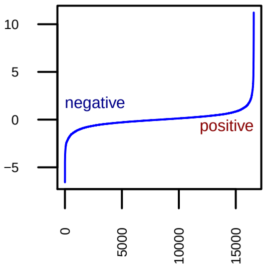

375 negative	392 positive
CSMD1	OAS1
NRTN	DDX58
GATA4	OAS3
PRKG2	OAS2
HPGD	IFIH1
DPP10	IFIT3
MYOM2	IFIT2
CPN1	IFI6
KIAA1456	BST2
RP11−599J14.2	XAF1
BAI1	RSAD2
DPP10−AS1	CMPK2
RP11−252C15.1	TCN1
TNFRSF10B	OASL
RP11−317N12.1	DDX60
RP13−735L24.1	IFI44L
NEFM	IFI44
MYT1	HERC6
LINC00221	HERC5
LONRF1	GFRA3
NELL1	LGSN
C8orf48	MX1
EPHX2	IFI27
CHIT1	DDX60L
KBTBD11	MX2
RNF175	USP18
SLC18A1	IRF7
SLC7A2	IFIT1
DUSP4	IFITM1
BAAT	ISG15
HR	RP11−197K6.1
CLN8	HELZ2
CYTL1	SAMD9L
NEIL2	LINC00462
CTB−36O1.7	LRRTM1
LINC01447	RP11−119J18.1
DPYSL5	BANCR
CD1E	ANGPTL7
OPRD1	SLC25A48
FRG2DP	COL2A1
LIN28A	NRXN3
HLA−DQB1−AS1	MYEOV
CTD−3006G17.2	EPSTI1
LINC00681	PARP9
AQP4	GBP1P1
CDO1	CCNYL2
ASAH1	TTC39A
DSCR8	RNF128
D4S234E	DHX58
DCLK1	LINC01426
CSPG4	APOBEC3A
DLC1	CXCL11
MTMR7	IFITM3
RP11−734I18.1	POU4F1
LGI3	DTX3L
MAGEA1	TRIM22
PNMA2	PARP12
PRR9	CTD−2521M24.9
RP11−582J16.5	ASB11
CHST9	PARP14
RP11−182J1.14	IGF2BP1
UBXN8	STEAP1B
ATP6V1B2	SAMD9
PEG3	UBE2L6
DSCAML1	PAGE5
BIN3	GRIN2B
STAR	ZNF560
AC110781.3	RP11−588H23.3
HBA2	RTP4
PBK	RP11−231N3.1
HS3ST5	TMEM255A
PRSS56	NKX2−5
CLDN11	BATF2
RP11−796A5.3	LRRTM4
EGR3	DAPL1
GRM7	CST2
C5orf17	IFI35
LIN28B	CCL8
GPAT2	SP110
WT1	PSMB9
CTD−2336O2.1	PTPRU
LZTS1	AP000593.7

GO:BP neg : 0 terms(FDR<0.01)

GO:BP pos : 132 terms(FDR<0.01)

Term	FDR
response to type I interferon	5.01e−28
type I interferon signaling pathway	5.01e−28
cellular response to type I interferon	5.01e−28
defense response to virus	2.67e−22
defense response to other organism	2.47e−21
defense response	1.33e−19
response to virus	3.01e−19
cytokine−mediated signaling pathway	1.84e−18
innate immune response	2.51e−17
response to cytokine	3.31e−17
response to interferon−gamma	7.79e−17
cellular response to cytokine stimulus	1.88e−16
interferon−gamma−mediated signaling path...	9.02e−15
response to other organism	3.11e−14
response to external biotic stimulus	3.11e−14
response to biotic stimulus	2.73e−13
cellular response to interferon−gamma	2.83e−13
negative regulation of viral genome repl...	9.19e−13
negative regulation of viral process	1.19e−12
negative regulation of viral life cycle	1.73e−12

GO:CC neg : 19 terms(FDR<0.01)

Term	FDR
plasma membrane part	5.87e−03
integral component of membrane	5.87e−03
intrinsic component of membrane	5.87e−03
intrinsic component of plasma membrane	7.79e−03
integral component of plasma membrane	1.06e−02
plasma membrane	1.44e−02
cell periphery	1.44e−02
postsynapse	1.54e−02
neurofibrillary tangle	1.77e−02
membrane part	1.80e−02

GO:CC pos : 8 terms(FDR<0.01)

Term	FDR
MHC class I protein complex	4.22e−06
nucleosome	6.05e−04
DNA packaging complex	1.71e−03
MHC protein complex	2.20e−03
extracellular region	1.06e−02
phagocytic vesicle membrane	2.44e−02
integral component of lumenal side of en...	3.44e−02
lumenal side of endoplasmic reticulum me...	3.44e−02

GO:MF neg : 5 terms(FDR<0.01)

Term	FDR
receptor activity	3.65e−04
molecular transducer activity	3.65e−04
transmembrane signaling receptor activit...	1.61e−03
transmembrane receptor activity	1.61e−03
signaling receptor activity	3.26e−03

GO:MF pos : 19 terms(FDR<0.01)

Term	FDR
transmitter−gated ion channel activity	9.02e−04
transmitter−gated channel activity	9.02e−04
double−stranded RNA binding	9.02e−04
peptide antigen binding	6.23e−03
2'−5'−oligoadenylate synthetase activity	7.36e−03
extracellular ligand−gated ion channel a...	7.36e−03
neurotransmitter receptor activity	7.36e−03
ionotropic glutamate receptor activity	8.59e−03
extracellular−glutamate−gated ion channe...	8.59e−03
TAP binding	1.60e−02

Cox regression:
logtest pv=4.5e−01
LHR=−0.69 (CI = −2.46, 1.09)

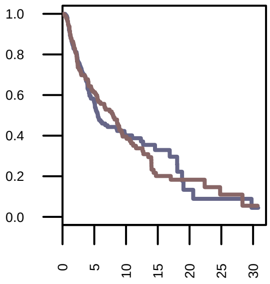

factor	p.value
Dataset	4.96e−06
SampleType	4.12e−04
Cluster	3.29e−01
Gender	5.17e−01

Dataset
pv=5.0e−06

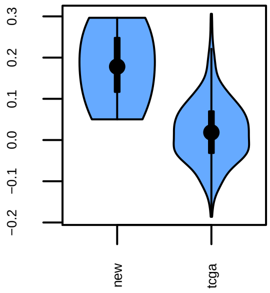

SampleType
pv=4.1e−04

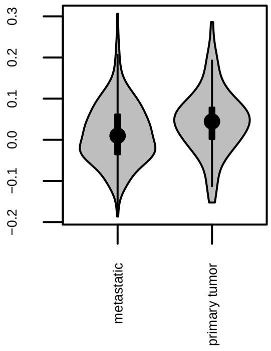

Cluster
pv=3.3e−01

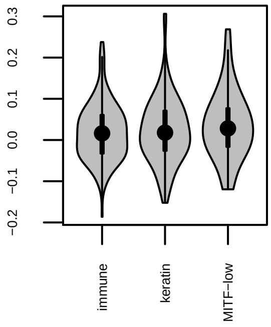

Gender
pv=5.2e−01

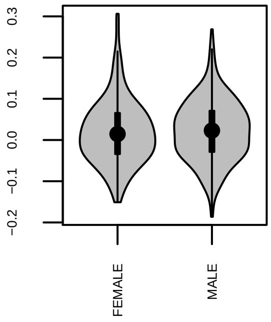

RIC49 (stability 0.813)

Metagene (involvement of features)

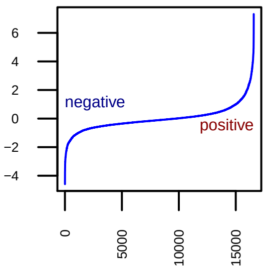

295 negative	912 positive
CNTN3	MYOC
TFAP2B	FLT4
LINC01317	ANO2
SEMA3E	TIE1
ENTHD1	GPR116
PLA2G2A	MRVI1
LINC01563	FOLH1
RNF182	ANGPT2
LRRC15	VWF
PSAT1	MGP
BANCR	SMOC2
CNR1	CDH6
SHC4	MYCT1
ACTN2	COL21A1
ATP8A2	CTAG2
FDCSP	F2RL3
NMRK2	DLL4
RP11−438B23.2	CNN1
MMP13	NGF
FXYD7	RERG
RP5−884M6.1	TRPC6
KLF14	ENPEP
GFRA1	TINAGL1
MDGA2	HAPLN1
PCK1	CXorf36
FHAD1	PAMR1
MAGEA11	ESAM
ADCY2	JPH2
RP4−718J7.4	SPARCL1
SOX9	ROBO4
AC009784.3	SLC6A1
ACTBP8	ACAN
RFPL2	FRZB
RNLS	ACTG2
UGT2B7	INHBB
SOX9−AS1	LMOD1
CILP	DCLK3
PNLIPRP3	HEYL
NTNG1	ESM1
ESRRB	AVPR1A
POU3F3	PPP1R14A
CD109	SDPR
MIR4697HG	NPNT
CXCL14	GPRC5C
SLC13A3	APLN
PLEKHS1	CD34
GS1−309P15.2	CLEC14A
TRPM1	MKRN3
AIM2	CDH5
FABP6	CLDN5
BCL2A1	NDUFA4L2
CRB1	GABRD
WISP2	PEAR1
OTOR	HBA2
B4GALNT4	NRARP
FRMD5	LRRC10B
RP11−348B17.1	OLIG2
LTK	EBF2
GRIN2A	TMEM233
NLRP11	HBB
RP11−63E9.1	GPIHBP1
GPR158	CALCRL
PCLO	ABCC9
RP11−347E10.1	PLVAP
GRIK2	MAPK4
SFRP2	FOXC2
IGSF9B	PLN
GPR150	SSTR1
C8orf46	TAGLN
VEPH1	GUCY1A2
GLDC	GRIK3
DRD2	FOXS1
MMP7	NAA11
ITGB8	BCL6B
BZRAP1	CYP4X1
RP5−907D15.4	KCNJ8
CPSF1P1	PODXL
IGHV2−70	ACTA2
TCAM1P	GPR4
ZNF280A	NXPH4
TMTC2	GJA4
LINC01579	LINC01436

GO:BP neg : 80 terms(FDR<0.01)

Term	FDR
single−multicellular organism process	1.13e−03
multicellular organismal process	1.35e−03
developmental process	1.47e−03
cell−cell signaling	1.47e−03
regulation of membrane potential	2.35e−03
regulation of peptide hormone secretion	2.58e−03
single−organism developmental process	2.58e−03
peptide hormone secretion	3.01e−03
multicellular organism development	3.01e−03
anatomical structure development	3.31e−03
system process	6.97e−03
nervous system development	7.52e−03
system development	7.52e−03
regulation of peptide secretion	7.91e−03
skeletal system development	7.91e−03
insulin secretion	7.91e−03
single organism signaling	7.91e−03
signaling	7.91e−03
regulation of insulin secretion	7.91e−03
cell communication	8.27e−03

GO:BP pos : 982 terms(FDR<0.01)

Term	FDR
multicellular organismal process	1.50e−27
single−multicellular organism process	8.27e−27
circulatory system development	1.65e−25
blood vessel development	6.01e−24
angiogenesis	6.01e−24
cardiovascular system development	6.01e−24
vasculature development	6.44e−24
multicellular organism development	2.26e−23
system development	2.41e−22
anatomical structure development	2.41e−22
developmental process	8.47e−22
blood vessel morphogenesis	9.65e−22
single−organism developmental process	1.62e−21
anatomical structure morphogenesis	3.01e−20
system process	1.20e−19
single organism signaling	6.01e−19
cell communication	6.19e−19
signaling	8.35e−19
anatomical structure formation involved ...	2.37e−17
circulatory system process	1.43e−16

GO:CC neg : 28 terms(FDR<0.01)

Term	FDR
plasma membrane part	4.58e−05
integral component of plasma membrane	4.58e−05
intrinsic component of plasma membrane	4.58e−05
cell periphery	4.58e−05
plasma membrane	1.17e−04
synapse	9.47e−04
intrinsic component of membrane	1.86e−03
synapse part	1.86e−03
neuron part	6.17e−03
myosin filament	6.17e−03

GO:CC pos : 49 terms(FDR<0.01)

Term	FDR
cell periphery	1.04e−25
plasma membrane	6.78e−25
extracellular space	2.26e−23
plasma membrane part	1.01e−21
intrinsic component of plasma membrane	2.05e−21
extracellular matrix	1.96e−20
integral component of plasma membrane	7.86e−20
intrinsic component of membrane	5.04e−17
extracellular region	1.85e−16
proteinaceous extracellular matrix	2.38e−16

GO:MF neg : 22 terms(FDR<0.01)

Term	FDR
metal ion transmembrane transporter acti...	4.72e−03
cation channel activity	4.72e−03
voltage−gated cation channel activity	7.01e−03
channel activity	9.88e−03
passive transmembrane transporter activi...	9.88e−03
transmembrane signaling receptor activit...	9.88e−03
inorganic cation transmembrane transport...	9.88e−03
gated channel activity	9.88e−03
ion channel activity	9.88e−03
signaling receptor activity	9.88e−03

GO:MF pos : 89 terms(FDR<0.01)

Term	FDR
transmembrane receptor activity	7.16e−16
receptor activity	7.16e−16
molecular transducer activity	7.16e−16
signaling receptor activity	1.93e−15
transmembrane signaling receptor activit...	2.92e−15
growth factor binding	1.00e−11
G−protein coupled receptor activity	1.10e−11
receptor binding	1.34e−10
glycosaminoglycan binding	1.43e−10
signal transducer activity	3.05e−09

Cox regression: logtest pv=2.4e−01 LHR=0.62 (CI = −0.41, 1.66)

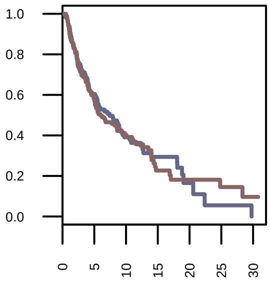

factor	p.value
SampleType	1.60e−03
Cluster	2.04e−02
Dataset	6.26e−02
Gender	4.30e−01

SampleType pv=1.6e−03

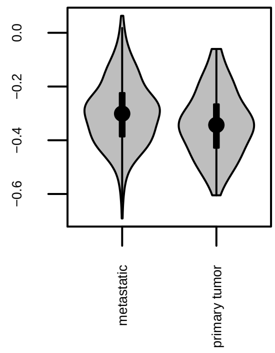

Cluster pv=2.0e−02

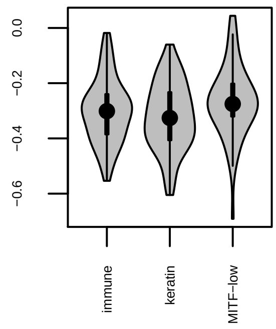

Dataset pv=6.3e−02

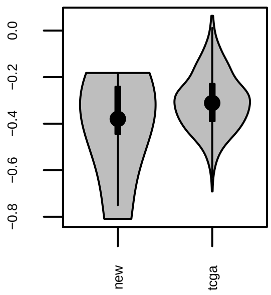

Gender pv=4.3e−01

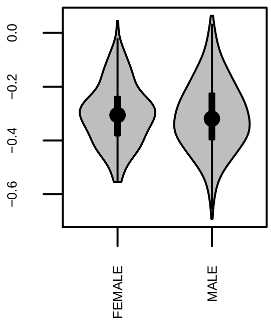

RIC55 (stability 0.963)

Metagene
(involvement of features)

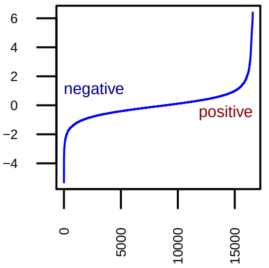

113 negative	383 positive
LINC01021	DEPDC1
TCERG1L	POLQ
GLB1L2	ASPM
CST1	RAD54L
CLUL1	BIRC5
LINC00648	MYBL2
SEZ6L2	FAM83D
FAM155B	NCAPG
PCDHGA7	FOXM1
FBXO2	TTK
LINC00086	KIF20A
PODNL1	CDC20
ABCC11	NEK2
SHC2	NCAPH
CD177	DLGAP5
EDA2R	FAM64A
PTCHD4	SGOL1
ZFP57	TOP2A
PNMA3	TROAP
EDN3	ESPL1
RP11−3L21.2	NUF2
DNAH5	MKI67
PTGDS	SKA1
APOD	CCNB2
HOXC12	KIF15
LRAT	SKA3
SERPINI1	KIAA0101
ADARB2	PLK1
RP5−1007F24.1	PBK
SPATA18	CKAP2L
CELF5	ESCO2
CCDC184	RRM2
SOD3	EXO1
RORC	AURKB
TRIML2	KIF18B
RP11−295M3.4	ERCC6L
COL8A1	ANLN
CRTAC1	MCM10
SCG3	CLSPN
TRAM1L1	CDC45
CHGB	CENPF
KCNJ13	KIF14
CDH19	HJURP
PCSK2	BUB1B
SALL1	UBE2C
ANGPTL7	GSG2
HCN2	GTSE1
NANOS1	CASC5
GRIA2	TICRR
NPTXR	CDCA5
CNTN4	TERT
LDHD	FAM111B
LYNX1	SPC25
RP11−760H22.2	MELK
AC005330.2	KIFC1
PRUNE2	KIF4A
RGS11	CDC25C
PLAG1	NUSAP1
EEF1A2	ASF1B
DPP6	CENPE
MTRNR2L1	KIF2C
SYNPR	DTL
C16orf45	DIAPH3
FAM135B	CDCA7
SERPINA3	ARHGAP11A
TPD52L1	E2F7
SNED1	E2F8
HCN1	CENPA
ATP1B2	PRC1
SGSM1	UHRF1
CDK5R2	KIF18A
SEPP1	IQGAP3
SERPINA3	CDCA8
NME5	PKMYT1
GABRB1	TPX2
TMEM151A	CDCA2
KLF8	ORC1
GDF15	HMMR
GALNT14	TK1
RP6−24A23.7	BUB1
RP6−24A23.3	CEP55
HPN−AS1	CDK1

GO:BP neg : 0 terms(FDR<0.01)

GO:BP pos : 388 terms(FDR<0.01)

Term	FDR
cell cycle	6.54e−29
cell cycle process	6.54e−29
mitotic cell cycle	6.54e−29
mitotic cell cycle process	6.54e−29
nuclear division	6.54e−29
organelle fission	6.54e−29
chromosome segregation	6.54e−29
mitotic nuclear division	6.54e−29
nuclear chromosome segregation	6.54e−29
cell division	6.54e−29
chromosome organization	6.54e−29
sister chromatid segregation	6.54e−29
DNA metabolic process	6.54e−29
DNA replication	6.54e−29
cell cycle phase transition	6.54e−29
mitotic cell cycle phase transition	6.54e−29
regulation of cell cycle	6.54e−29
organelle organization	6.54e−29
sister chromatid cohesion	6.54e−29
DNA−dependent DNA replication	6.54e−29

GO:CC neg : 1 terms(FDR<0.01)

Term	FDR
HCN channel complex	7.88e−02

GO:CC pos : 120 terms(FDR<0.01)

Term	FDR
chromosome	1.53e−29
chromosomal part	1.53e−29
chromosomal region	1.53e−29
condensed chromosome	1.53e−29
chromosome, centromeric region	1.53e−29
condensed chromosome, centromeric region	1.53e−29
kinetochore	1.53e−29
non−membrane−bounded organelle	1.53e−29
intracellular non−membrane−bounded organ...	1.53e−29
condensed chromosome kinetochore	1.53e−29

GO:MF neg : 3 terms(FDR<0.01)

Term	FDR
intracellular cAMP activated cation chan...	6.87e−02
ligand−gated ion channel activity	6.87e−02
ligand−gated channel activity	6.87e−02

GO:MF pos : 67 terms(FDR<0.01)

Term	FDR
single−stranded DNA−dependent ATPase act...	2.32e−11
DNA−dependent ATPase activity	1.46e−10
DNA helicase activity	3.72e−10
ATP binding	2.04e−09
adenyl ribonucleotide binding	5.15e−09
adenyl nucleotide binding	6.44e−09
ATPase activity	1.10e−08
chromatin binding	2.84e−08
microtubule motor activity	3.58e−08
DNA binding	7.73e−08

Cox regression:
logtest pv=3.0e−02
LHR=1.09 (CI = 0.10, 2.07)

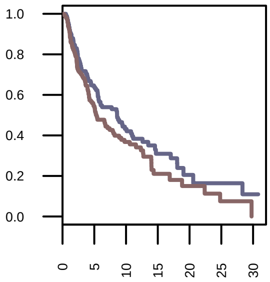

factor	p.value
SampleType	7.41e−02
Gender	1.17e−01
Dataset	3.12e−01
Cluster	3.13e−01

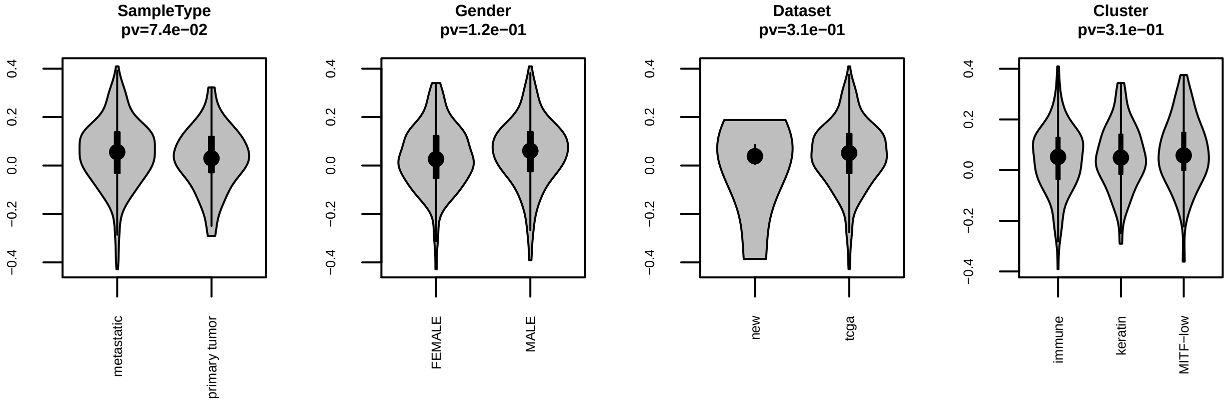

RIC57 (stability 0.805)

Metagene (involvement of features)

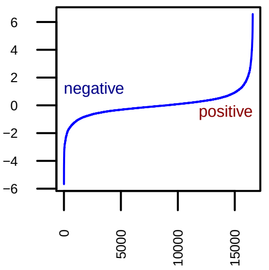

356 negative	607 positive
FRG2DP	STAB1
LGALS17A	SLC11A1
CXCL11	MARCO
ART3	MSR1
CXCL13	CD84
IDO1	CD209
GBP1P1	TREM2
IFNG	AQP9
BEX1	CD33
XIRP1	LILRB5
OR2I1P	HAMP
RAB39B	MS4A4A
LINC01474	LY86
RDH8	IGSF21
CTLA4	SPP1
ZBED2	KCNJ5
GBP4	ADORA3
COL22A1	HS3ST2
EXOC3L4	F13A1
CXCL9	TREM1
FBXO2	NKX2−2
TOX	SIGLEC9
RP11−84D1.2	ALOX5AP
PRSS12	LYVE1
AC145110.1	CD180
SNCB	SLCO2B1
GBP1	SLC2A5
MAGEB2	VSIG4
ICOS	CCR1
CXCL10	DCSTAMP
EOMES	CYBB
CEACAM1	FOLR2
BAAT	LAIR1
MKRN3	CD300A
PPP1R3C	SIGLEC7
UBD	ITGAM
GLB1L3	CD14
MS4A15	GPR34
EFS	OLR1
PKLR	CD163
STEAP2	CSF1R
ETV7	LILRB4
CD5L	FPR3
KIAA1244	TLR7
MIAT	C5AR1
OR2A1−AS1	MPEG1
TSPEAR	CR1
KIAA1045	FCGR3A
PYGO1	SEPP1
TMEM215	CLEC5A
CTC−453G23.5	MRC1
ZFP57	FCGBP
TSPEAR−AS1	SIRPB2
PDCD1	LILRA2
BNC1	CD86
CKMT2	C1QC
TSPEAR−AS2	FBP1
BBOX1	LRRC25
GZMB	NPTX1
BARX2	C3AR1
C1orf106	EYA4
PSPHP1	C1QB
BCAN	LILRA5
MYH14	CD300C
ACY3	OSCAR
HCG4P5	MS4A6A
GZMH	PILRA
LPAR3	SIGLEC1
FAM19A5	LAPTM5
HCP5	CD163L1
ZNF503−AS1	ATP6V0D2
RP11−3L21.2	PRIMA1
KIF12	FPR1
MYT1	FCER1A
MMP3	TYROBP
CDKN2A	RNASE6
DTHD1	SIGLEC14
RP1−40E16.9	ALOX5
LINC01314	ITGB2
FASLG	CMKLR1
RP11−1094M14.8	LILRA6
HKDC1	LILRB1

GO:BP neg : 133 terms(FDR<0.01)

Term	FDR
defense response	8.42e−10
immune system process	3.16e−07
immune response	8.52e−07
T cell activation	1.13e−06
type I interferon signaling pathway	9.52e−06
cellular response to type I interferon	9.52e−06
innate immune response	9.88e−06
response to type I interferon	1.43e−05
lymphocyte activation	1.84e−05
cytokine−mediated signaling pathway	1.91e−05
regulation of immune system process	1.91e−05
regulation of T cell activation	4.01e−05
defense response to virus	4.01e−05
response to other organism	4.01e−05
response to external biotic stimulus	4.01e−05
response to biotic stimulus	4.32e−05
response to interferon−gamma	4.51e−05
regulation of leukocyte cell−cell adhesi...	7.18e−05
response to cytokine	7.91e−05
cellular response to cytokine stimulus	9.77e−05

GO:BP pos : 790 terms(FDR<0.01)

Term	FDR
immune response	2.15e−28
immune system process	2.15e−28
defense response	2.15e−28
leukocyte activation	2.15e−28
immune effector process	2.15e−28
cell activation	2.15e−28
leukocyte mediated immunity	2.15e−28
regulation of immune system process	3.76e−27
inflammatory response	7.35e−27
regulation of immune response	2.41e−26
leukocyte activation involved in immune ...	6.15e−26
cell activation involved in immune respo...	7.27e−26
myeloid leukocyte activation	1.50e−24
response to stimulus	4.19e−22
regulated exocytosis	6.32e−22
secretion	1.32e−21
secretion by cell	4.42e−21
positive regulation of immune system pro...	5.51e−21
leukocyte degranulation	1.66e−20
innate immune response	2.78e−20

GO:CC neg : 4 terms(FDR<0.01)

Term	FDR
side of membrane	6.97e−03
plasma membrane part	7.33e−03
external side of plasma membrane	1.15e−02
T cell receptor complex	1.15e−02

GO:CC pos : 70 terms(FDR<0.01)

Term	FDR
plasma membrane	2.29e−29
cell periphery	2.29e−29
membrane part	2.29e−29
plasma membrane part	2.29e−29
intrinsic component of membrane	2.29e−29
integral component of membrane	2.29e−29
integral component of plasma membrane	2.29e−29
intrinsic component of plasma membrane	2.29e−29
secretory granule	3.05e−20
secretory vesicle	7.33e−20

GO:MF neg : 7 terms(FDR<0.01)

Term	FDR
cytokine activity	1.59e−04
CXCR3 chemokine receptor binding	2.79e−04
chemokine activity	2.72e−03
chemokine receptor binding	1.37e−02
cytokine receptor binding	1.37e−02
CXCR chemokine receptor binding	5.73e−02
MHC protein binding	8.59e−02

GO:MF pos : 71 terms(FDR<0.01)

Term	FDR
receptor activity	2.15e−28
molecular transducer activity	2.15e−28
signaling receptor activity	9.88e−22
transmembrane receptor activity	1.29e−20
transmembrane signaling receptor activit...	2.58e−20
signal transducer activity	3.51e−18
carbohydrate binding	9.81e−08
cargo receptor activity	1.40e−06
G−protein coupled receptor activity	2.10e−06
signaling pattern recognition receptor a...	5.73e−06

Cox regression: logtest pv=5.3e−01 LHR=0.45 (CI = −0.96, 1.86)

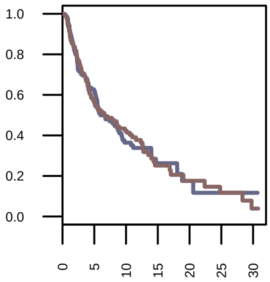

factor	p.value
Dataset	9.83e−08
SampleType	1.11e−05
Cluster	3.20e−04
Gender	6.28e−01

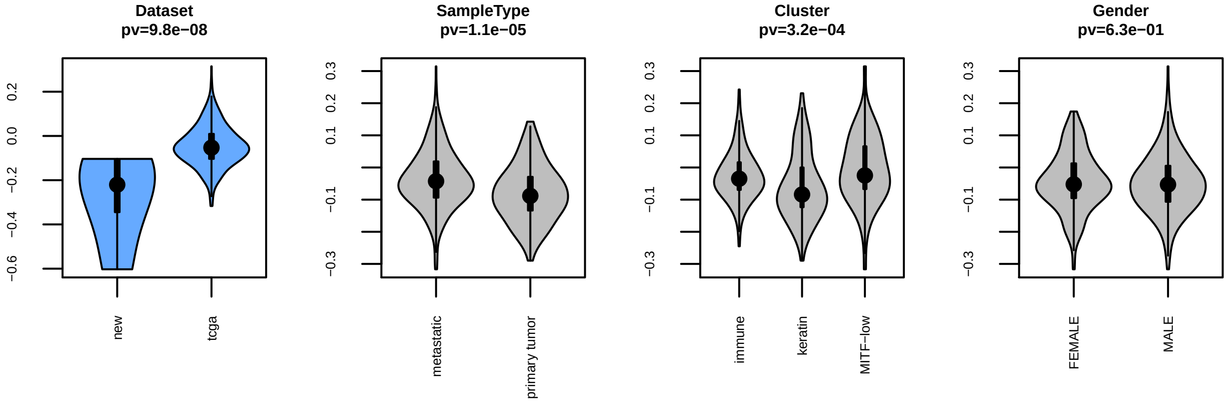

RIC74 (stability 0.532)

Metagene (involvement of features)

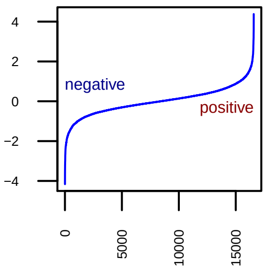

100 negative	108 positive
BFSP2	CCNYL2
PIP5K1B	DAPL1
ROBO2	SOSTDC1
B3GAT1	APCDD1L
PLA1A	GFRA3
IGKV2−29	RASGEF1C
MAGEC1	LRRTM4
RIMS4	ST8SIA6−AS1
PRRT4	FZD9
LRRC4B	LINC00839
SYT6	BVES
UGT8	CHRDL1
KLHL14	NCAM1
IGLL3P	C1QL4
OLFM3	OTX1
TFF2	NPY1R
DNASE2B	IL22RA1
TMEM255A	GPR143
CDH15	GAL
RP11−307C19.2	IGFN1
TTLL6	IDI2−AS1
TTC39A	CCL21
ATP12A	DCAF12L1
SLC17A9	EFHD1
TRGV5	RP11−809C18.3
CD79B	APCDD1L−AS1
CAMK2B	COL20A1
DERL3	IGLV3−1
VSTM2L	GRIA1
ANO5	AC110781.3
LINC01474	CASC15
AMPH	RP11−865I6.2
KLHL6	FERMT1
MME	CDH12
IGKV1D−33	DSCR8
IGKV1D−12	ST8SIA6
IGKV2D−30	TET1
IGKV2D−28	ISM1
LINC00494	NKX2−5
MYO5B	LINC01436
JPH4	AHNAK2
RP11−81H14.2	PTPLA
EAF2	POPDC3
FAM78A	ABCC11
CLEC11A	PALM3
TPSB2	KC6
KLK1	BOLA3−AS1
SPP1	MAT1A
PKLR	TFAP2B
CNR1	ERVMER61−1
PTGDS	AHRR
NUDT10	SOX2
CD22	REN
SH2D1B	KCNIP3
IGKV1D−43	RP5−984P4.6
MTND4P12	HNF4G
DCX	ABCA8
IL24	CDHR3
SYT3	TDRP
EYA1	C10orf82
IGKV1D−39	RAC3
CTA−292E10.9	DLX2
TF	RP11−132A1.4
HSPA7	RGR
GRAP	CCL19
ATP1A4	MTRNR2L1
COCH	SOX9−AS1
CXCL1	HS6ST3
KCNK10	HOXC8
IGKV1−39	ZNF239
TMEM71	RP11−838N2.5
SHANK1	LOXL4
RP11−404F10.2	FBN3
KIF17	ADH1B
RP4−765C7.2	OGDHL
KBTBD8	MEX3A
CLEC12B	TRPM1
IGKV2OR22−4	BAIAP2L1
HAO2	ROR1−AS1
IGLV9−49	SLC45A2
RP5−1086K13.1	PHYHIP
PRSS30P	LINC01249

GO:BP neg : 0 terms(FDR<0.01)

GO:BP pos : 0 terms(FDR<0.01)

GO:CC neg : 0 terms(FDR<0.01)

GO:CC pos : 0 terms(FDR<0.01)

GO:MF neg : 0 terms(FDR<0.01)

GO:MF pos : 0 terms(FDR<0.01)

Cox regression:
logtest pv=1.2e−05
LHR=3.27 (CI = 1.75, 4.79)

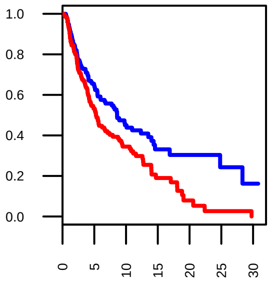

factor	p.value
Cluster	4.99e−17
SampleType	2.08e−05
Gender	1.18e−02
Dataset	4.59e−02

Cluster
pv=5.0e−17

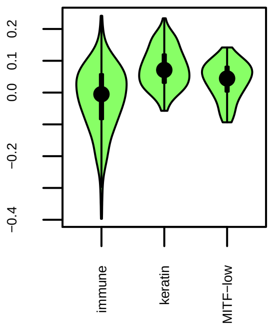

SampleType
pv=2.1e−05

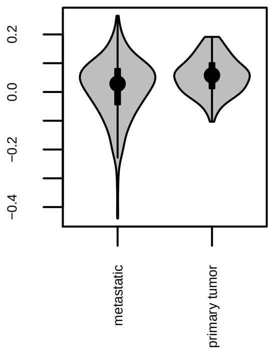

Gender
pv=1.2e−02

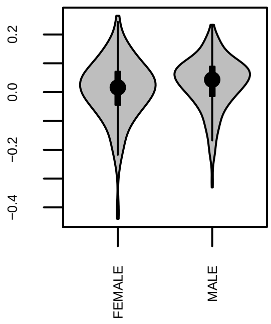

Dataset
pv=4.6e−02

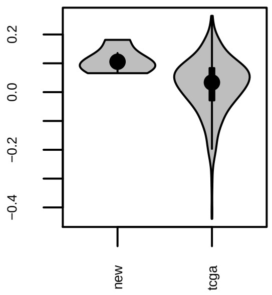

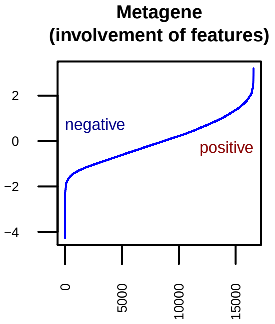

0
negative

0
positive

RIC75 (stability 0.914)

GO:BP neg : 0 terms(FDR<0.01)

GO:BP pos : 0 terms(FDR<0.01)

GO:CC neg : 0 terms(FDR<0.01)

GO:CC pos : 0 terms(FDR<0.01)

GO:MF neg : 0 terms(FDR<0.01)

GO:MF pos : 0 terms(FDR<0.01)

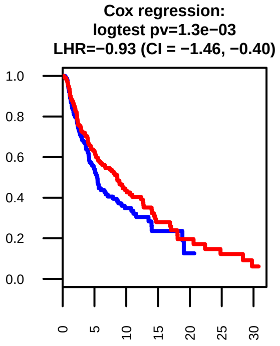

factor	p.value
Cluster	1.69e−08
Dataset	1.07e−07
SampleType	2.19e−05
Gender	1.11e−01

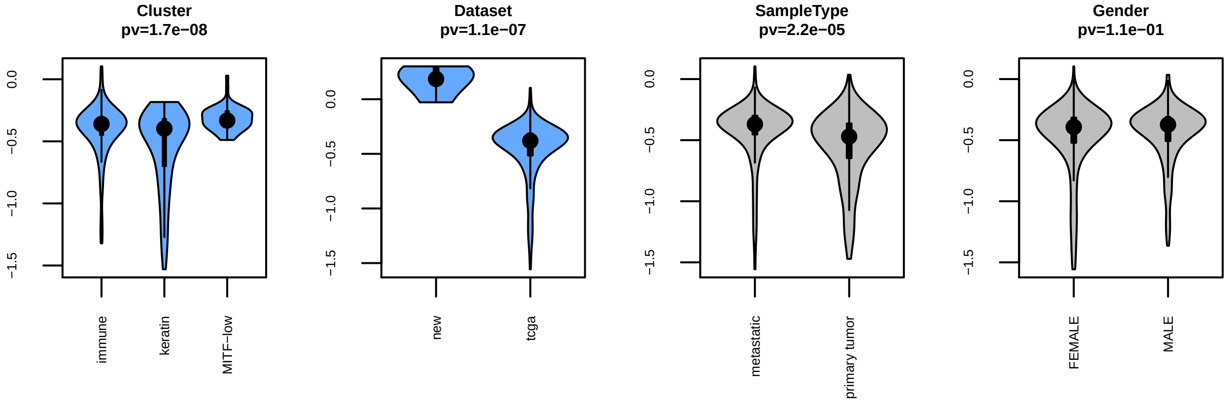

RIC79 (stability 0.704)

Metagene (involvement of features)

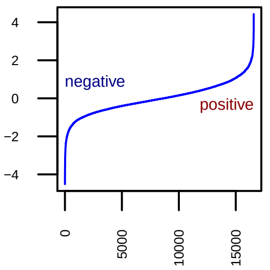

22 negative

TYRP1
RP11−284F21.7
CLUL1
CTAG2
RP11−706O15.3
COL9A1
SLC30A2
RP11−284F21.10
PHKA1
RP11−284F21.9
SLC6A17
ONECUT2
MT1A
GAPDHS
KLF14
MNX1
SULT4A1
MMACHC
GSG1L
IGSF9B
PAEP
HIST1H3H

26 positive

PRSS50
WFDC1
DAPL1
FXYD7
PPP1R14C
PSPHP1
RP11−369C8.1
RP11−575F12.3
COCH
ABCA6
KCNS3
LRRTM1
RGS9
SHANK2
TNNT1
AKR1C1
MAMDC2
SMPDL3B
PPAP2C
DCC
ARHGEF16
SIK1
SIX3
PLEKHH2
SLC38A11
RNF43

GO:BP neg : 0 terms(FDR<0.01)

GO:BP pos : 0 terms(FDR<0.01)

GO:CC neg : 0 terms(FDR<0.01)

GO:CC pos : 0 terms(FDR<0.01)

GO:MF neg : 0 terms(FDR<0.01)

GO:MF pos : 0 terms(FDR<0.01)

Cox regression: logtest pv=5.2e−03 LHR=−1.60 (CI = −2.73, −0.47)

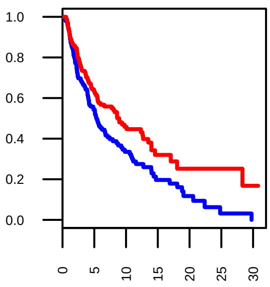

factor	p.value
Cluster	1.27e−11
SampleType	1.44e−02
Dataset	6.26e−01
Gender	7.80e−01

Cluster pv=1.3e−11 **SampleType pv=1.4e−02** **Dataset pv=6.3e−01** **Gender pv=7.8e−01**

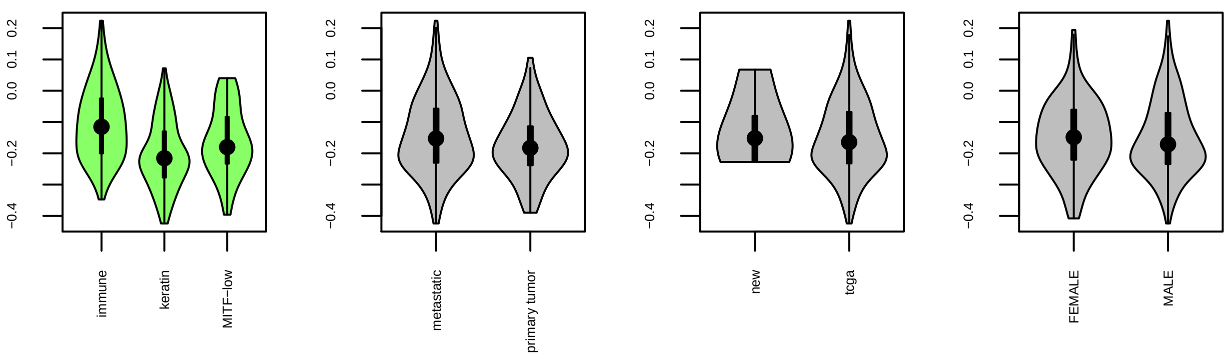

MIC1 (stability 0.934)

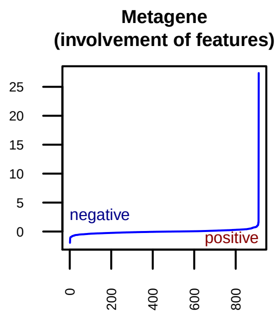

8 negative	10 positive
hsa−miR−205−5p	hsa−miR−146a−3p
hsa−miR−199b−5p	hsa−miR−338−5p
hsa−miR−876−5p	hsa−miR−551b−3p
hsa−miR−1266−5p	hsa−miR−598−3p
hsa−miR−301b−3p	hsa−miR−206
hsa−miR−3690	hsa−miR−34a−5p
hsa−miR−365a−3p	hsa−miR−338−3p
hsa−miR−125b−1−3p	hsa−miR−146a−5p
	hsa−miR−1269a
	hsa−miR−573

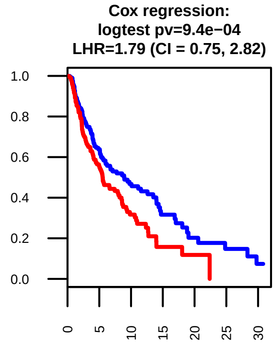

factor	p.value
Cluster	1.03e−01
SampleType	7.73e−01
Dataset	7.83e−01
Gender	8.70e−01

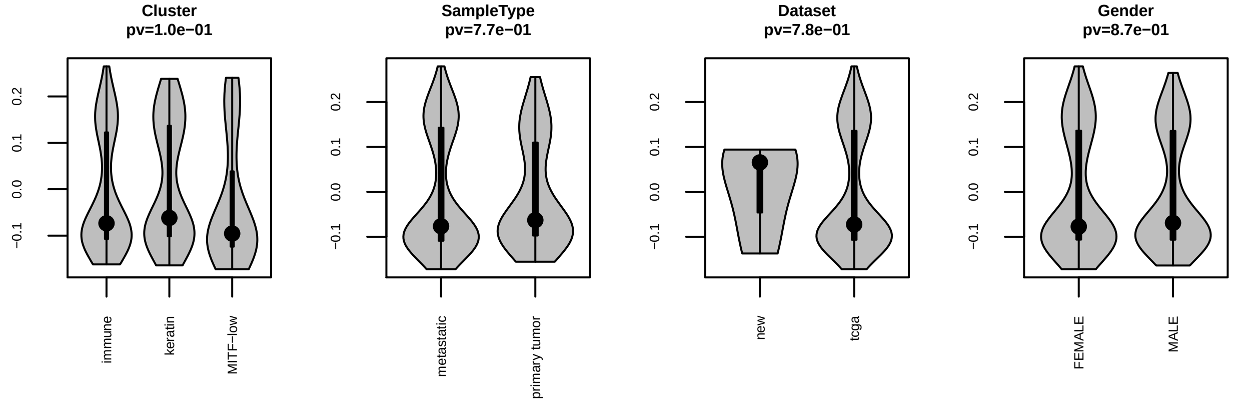

MIC9 (stability 0.988)

Metagene (involvement of features)

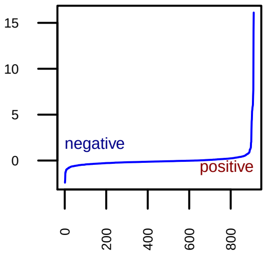

**18 negative**	**37 positive**
hsa−miR−675−3p	hsa−miR−141−3p
hsa−miR−217	hsa−miR−141−5p
hsa−miR−137	hsa−miR−200a−3p
hsa−miR−483−5p	hsa−miR−200a−5p
hsa−miR−216a−5p	hsa−miR−200b−3p
hsa−miR−1228−5p	hsa−miR−200b−5p
hsa−miR−483−3p	hsa−miR−200c−3p
hsa−miR−1−3p	hsa−miR−200c−5p
hsa−miR−504−5p	hsa−miR−203a−3p
hsa−miR−592	hsa−miR−205−3p
hsa−miR−215−5p	hsa−miR−205−5p
hsa−miR−2116−3p	hsa−miR−224−5p
hsa−miR−873−5p	hsa−miR−429
hsa−miR−378c	hsa−miR−944
hsa−miR−133a−3p	hsa−miR−31−3p
hsa−miR−1224−5p	hsa−miR−31−5p
hsa−miR−218−1−3p	hsa−miR−452−5p
hsa−miR−876−5p	hsa−miR−375
	hsa−miR−3161
	hsa−miR−1295a
	hsa−miR−1293
	hsa−miR−206
	hsa−miR−183−5p
	hsa−miR−27b−5p
	hsa−miR−224−3p
	hsa−miR−26a−2−3p
	hsa−miR−383−5p
	hsa−miR−223−3p
	hsa−miR−452−3p
	hsa−miR−577
	hsa−miR−1249−3p
	hsa−miR−27b−3p
	hsa−miR−149−5p
	hsa−miR−202−5p
	hsa−miR−188−5p
	hsa−miR−139−5p
	hsa−miR−885−5p

Cox regression:
logtest pv=2.9e−02
LHR=0.77 (CI = 0.12, 1.43)

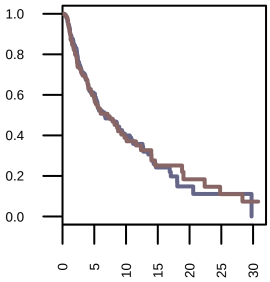

**factor**	**p.value**
SampleType	1.77e−49
Cluster	1.70e−18
Dataset	1.13e−03
Gender	3.27e−02

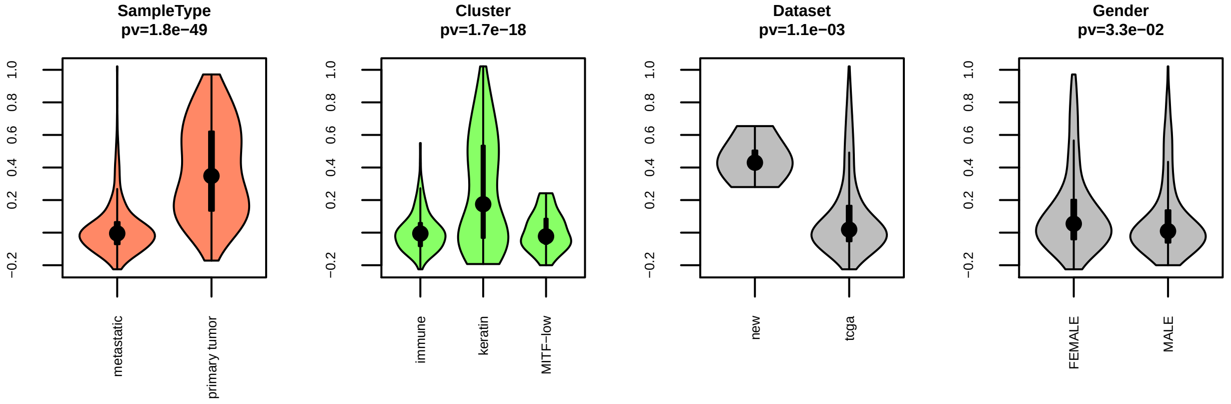

MIC11 (stability 0.994)

Metagene (involvement of features)

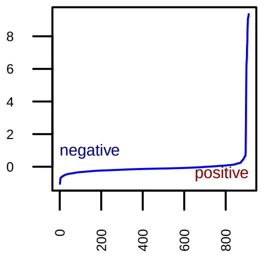

**12 negative**	**33 positive**
hsa−miR−3200−3p	hsa−miR−506−3p
hsa−miR−137	hsa−miR−507
hsa−miR−2355−3p	hsa−miR−508−3p
hsa−miR−296−5p	hsa−miR−508−5p
hsa−miR−452−3p	hsa−miR−509−3−5p
hsa−miR−548b−3p	hsa−miR−509−3p
hsa−miR−3170	hsa−miR−509−5p
hsa−miR−1287−5p	hsa−miR−510−5p
hsa−miR−149−5p	hsa−miR−513a−3p
hsa−miR−153−3p	hsa−miR−513a−5p
hsa−miR−548v	hsa−miR−513b−5p
hsa−miR−3922−3p	hsa−miR−513c−5p
	hsa−miR−514a−3p
	hsa−miR−514b−3p
	hsa−miR−514b−5p
	hsa−miR−3161
	hsa−miR−211−5p
	hsa−miR−204−5p
	hsa−miR−183−5p
	hsa−miR−219a−2−3p
	hsa−miR−3065−5p
	hsa−miR−1291
	hsa−miR−3065−3p
	hsa−miR−1243
	hsa−miR−138−1−3p
	hsa−miR−891a−5p
	hsa−miR−193b−3p
	hsa−miR−182−5p
	hsa−miR−205−5p
	hsa−miR−658
	hsa−miR−138−5p
	hsa−miR−31−5p
	hsa−miR−184

Cox regression: logtest pv=9.9e−02 LHR=0.30 (CI = −0.06, 0.67)

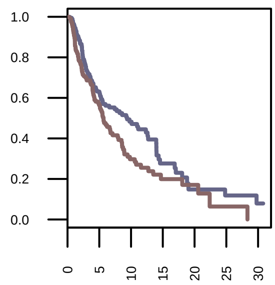

**factor**	**p.value**
Cluster	5.96e−15
SampleType	8.86e−06
Gender	4.84e−01
Dataset	8.42e−01

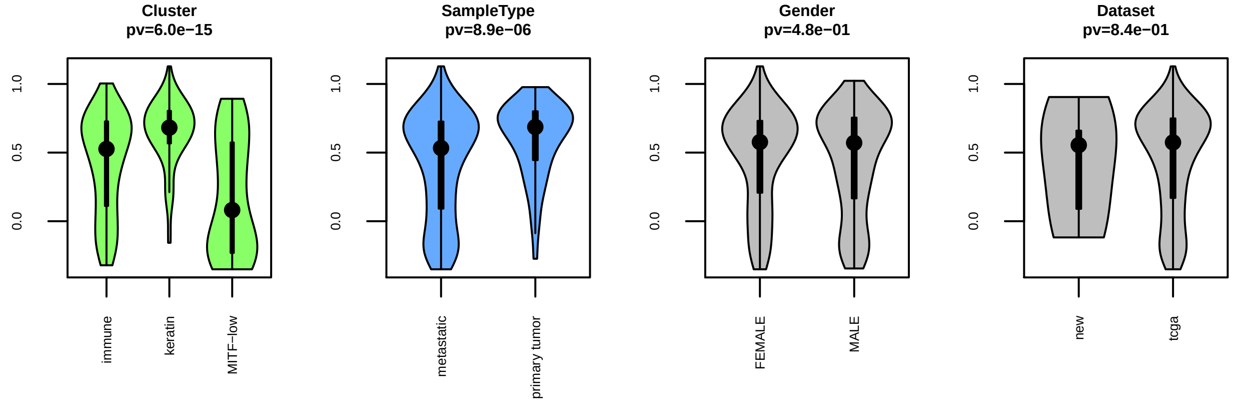

MIC14 (stability 0.891)

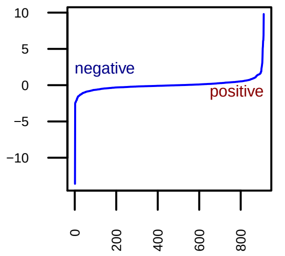

33 negative	39 positive
hsa−miR−211−5p	hsa−miR−100−5p
hsa−miR−1266−5p	hsa−miR−137
hsa−miR−130a−3p	hsa−miR−3161
hsa−miR−1247−5p	hsa−miR−551b−3p
hsa−miR−3150b−3p	hsa−miR−577
hsa−miR−615−3p	hsa−miR−885−5p
hsa−miR−873−5p	hsa−miR−9−3p
hsa−miR−146a−5p	hsa−miR−9−5p
hsa−miR−1287−5p	hsa−miR−598−3p
hsa−miR−483−3p	hsa−miR−125b−1−3p
hsa−miR−483−5p	hsa−miR−125b−5p
hsa−miR−378c	hsa−miR−135b−5p
hsa−let−7d−5p	hsa−miR−3200−3p
hsa−miR−548k	hsa−miR−592
hsa−miR−3170	hsa−miR−100−3p
hsa−miR−1301−3p	hsa−miR−149−5p
hsa−miR−1910−5p	hsa−miR−140−3p
hsa−miR−34a−5p	hsa−miR−542−3p
hsa−miR−195−3p	hsa−miR−424−3p
hsa−miR−29b−1−5p	hsa−miR−3681−5p
hsa−miR−130a−5p	hsa−miR−424−5p
hsa−miR−196a−5p	hsa−miR−542−5p
hsa−miR−1249−3p	hsa−miR−221−5p
hsa−miR−30b−3p	hsa−miR−140−5p
hsa−miR−193b−5p	hsa−miR−450a−5p
hsa−miR−29b−3p	hsa−miR−375
hsa−miR−193b−3p	hsa−miR−1468−5p
hsa−miR−582−3p	hsa−miR−944
hsa−let−7d−3p	hsa−miR−222−5p
hsa−miR−181a−2−3p	hsa−miR−551b−5p
hsa−miR−582−5p	hsa−miR−21−5p
hsa−miR−10a−3p	hsa−miR−221−3p
hsa−miR−378a−5p	hsa−miR−2355−5p
	hsa−miR−1305
	hsa−miR−892a
	hsa−miR−935
	hsa−miR−193a−3p
	hsa−miR−21−3p
	hsa−miR−92b−3p

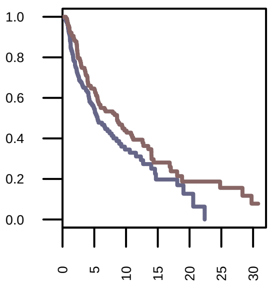

factor	p.value
Cluster	2.80e−20
SampleType	1.13e−03
Dataset	1.42e−01
Gender	7.55e−01

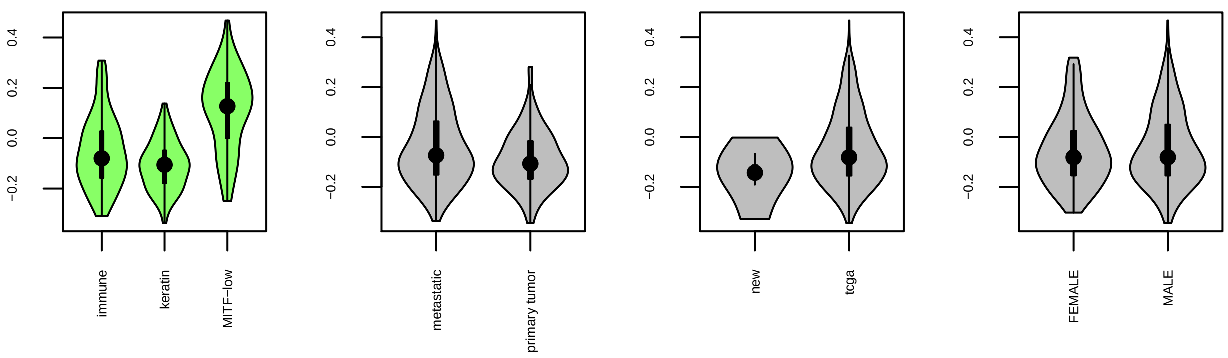

MIC20 (stability 0.962)

Metagene
(involvement of features)

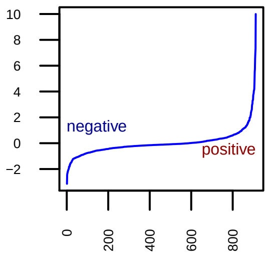

23
negative

hsa−miR−149−5p
hsa−miR−27b−5p
hsa−miR−205−5p
hsa−miR−27b−3p
hsa−miR−196b−5p
hsa−miR−1296−5p
hsa−miR−1262
hsa−miR−3170
hsa−miR−24−1−5p
hsa−miR−1305
hsa−miR−199b−5p
hsa−miR−151a−3p
hsa−miR−23b−3p
hsa−miR−582−5p
hsa−miR−196a−5p
hsa−miR−190a−5p
hsa−miR−887−3p
hsa−miR−30d−3p
hsa−miR−885−5p
hsa−miR−1−3p
hsa−miR−550a−5p
hsa−miR−20a−3p
hsa−miR−33a−5p

56
positive

hsa−miR−1247−5p
hsa−miR−142−3p
hsa−miR−142−5p
hsa−miR−146b−3p
hsa−miR−150−5p
hsa−miR−155−3p
hsa−miR−155−5p
hsa−miR−1976
hsa−miR−223−3p
hsa−miR−223−5p
hsa−miR−29c−3p
hsa−miR−342−3p
hsa−miR−342−5p
hsa−miR−3614−3p
hsa−miR−511−5p
hsa−miR−642a−5p
hsa−miR−146b−5p
hsa−miR−766−3p
hsa−miR−625−3p
hsa−miR−653−5p
hsa−miR−944
hsa−miR−29b−3p
hsa−miR−29b−2−5p
hsa−miR−3690
hsa−miR−29c−5p
hsa−miR−1228−5p
hsa−miR−148a−3p
hsa−miR−629−3p
hsa−let−7i−3p
hsa−miR−505−5p
hsa−miR−181a−3p
hsa−miR−361−3p
hsa−miR−708−3p
hsa−miR−3653−3p
hsa−miR−200c−3p
hsa−miR−486−3p
hsa−miR−330−5p
hsa−miR−708−5p
hsa−miR−618
hsa−miR−3667−5p
hsa−miR−1295a
hsa−miR−3678−5p
hsa−miR−148a−5p
hsa−miR−1270
hsa−miR−326
hsa−miR−548b−3p
hsa−miR−206
hsa−miR−21−5p
hsa−miR−3202
hsa−miR−21−3p
hsa−miR−942−5p
hsa−miR−1249−3p
hsa−miR−141−3p
hsa−miR−7−1−3p
hsa−miR−141−5p
hsa−miR−194−5p

Cox regression:
logtest pv=1.2e−04
LHR=−1.32 (CI = −1.99, −0.64)

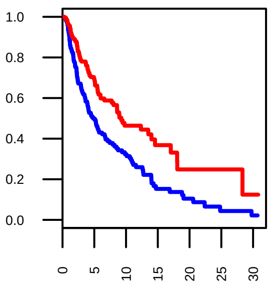

**factor**	**p.value**
Cluster	4.33e−38
SampleType	6.99e−03
Dataset	8.73e−03
Gender	1.67e−01

Cluster
pv=4.3e−38

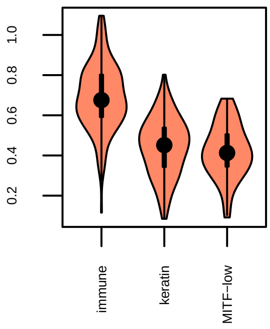

SampleType
pv=7.0e−03

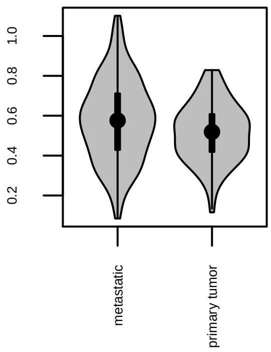

Dataset
pv=8.7e−03

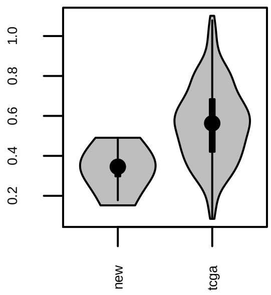

Gender
pv=1.7e−01

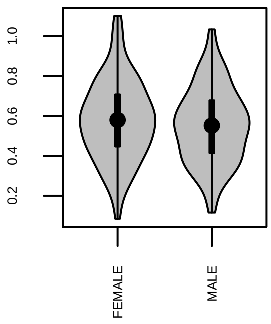

MIC22 (stability 0.982)

Metagene (involvement of features)

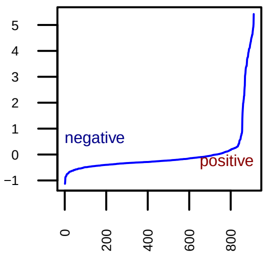

2 negative

hsa−miR−1305
hsa−miR−504−5p

71 positive

hsa−miR−127−3p
hsa−miR−127−5p
hsa−miR−134−5p
hsa−miR−136−3p
hsa−miR−136−5p
hsa−miR−154−3p
hsa−miR−154−5p
hsa−miR−299−3p
hsa−miR−299−5p
hsa−miR−323a−5p
hsa−miR−323b−3p
hsa−miR−329−3p
hsa−miR−337−3p
hsa−miR−369−3p
hsa−miR−369−5p
hsa−miR−370−3p
hsa−miR−376a−3p
hsa−miR−376a−5p
hsa−miR−376b−3p
hsa−miR−376c−3p
hsa−miR−377−3p
hsa−miR−377−5p
hsa−miR−379−3p
hsa−miR−379−5p
hsa−miR−380−3p
hsa−miR−380−5p
hsa−miR−381−3p
hsa−miR−382−5p
hsa−miR−409−3p
hsa−miR−409−5p
hsa−miR−410−3p
hsa−miR−411−3p
hsa−miR−411−5p
hsa−miR−431−3p
hsa−miR−431−5p
hsa−miR−432−5p
hsa−miR−433−3p
hsa−miR−485−3p
hsa−miR−485−5p
hsa−miR−487a−3p
hsa−miR−487b−3p
hsa−miR−493−3p
hsa−miR−493−5p
hsa−miR−495−3p
hsa−miR−496
hsa−miR−539−5p
hsa−miR−541−3p
hsa−miR−543
hsa−miR−654−3p
hsa−miR−654−5p
hsa−miR−655−3p
hsa−miR−656−3p
hsa−miR−668−3p
hsa−miR−758−3p
hsa−miR−889−3p
hsa−miR−541−5p
hsa−miR−770−5p
hsa−miR−203a−3p
hsa−miR−337−5p
hsa−miR−1185−5p
hsa−miR−708−3p
hsa−miR−873−5p
hsa−miR−223−3p
hsa−miR−412−3p
hsa−miR−199b−5p
hsa−miR−653−5p
hsa−miR−214−3p
hsa−miR−1193
hsa−miR−206
hsa−miR−625−3p
hsa−miR−125b−2−3p

Cox regression: logtest pv=6.4e−01 LHR=0.08 (CI = −0.27, 0.44)

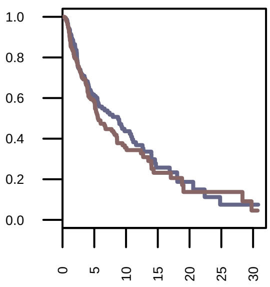

factor	p.value
Cluster	1.78e−05
Dataset	1.17e−01
SampleType	1.38e−01
Gender	5.37e−01

Cluster pv=1.8e−05

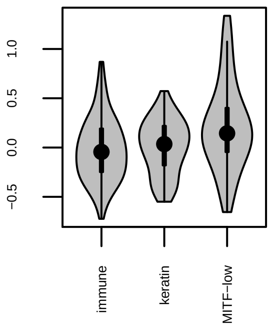

Dataset pv=1.2e−01

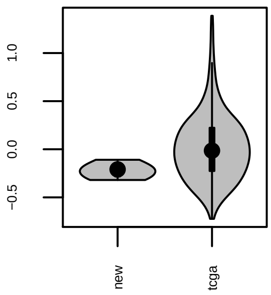

SampleType pv=1.4e−01

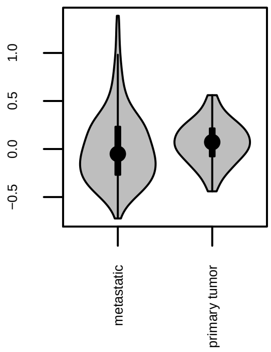

Gender pv=5.4e−01

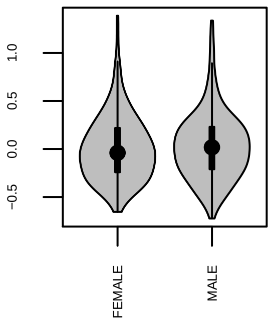

MIC25 (stability 0.875)

Metagene (involvement of features)

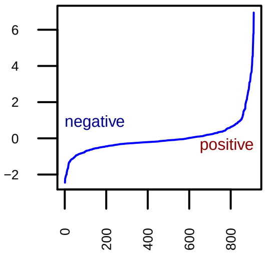

**18 negative**	**57 positive**
hsa−miR−221−5p	hsa−miR−1247−5p
hsa−miR−219a−2−3p	hsa−miR−143−5p
hsa−miR−194−3p	hsa−miR−145−3p
hsa−miR−206	hsa−miR−145−5p
hsa−miR−222−3p	hsa−miR−195−5p
hsa−miR−210−3p	hsa−miR−199a−3p
hsa−miR−221−3p	hsa−miR−199a−5p
hsa−miR−222−5p	hsa−miR−199b−3p
hsa−miR−135b−5p	hsa−miR−199b−5p
hsa−miR−323a−5p	hsa−miR−214−3p
hsa−miR−194−5p	hsa−miR−214−5p
hsa−miR−192−5p	hsa−miR−217
hsa−miR−541−5p	hsa−miR−483−3p
hsa−miR−30b−3p	hsa−miR−483−5p
hsa−miR−30d−5p	hsa−miR−497−5p
hsa−miR−625−3p	hsa−miR−675−3p
hsa−miR−378c	hsa−miR−887−3p
hsa−miR−3130−5p	hsa−miR−216a−5p
	hsa−miR−195−3p
	hsa−miR−708−5p
	hsa−miR−1−3p
	hsa−miR−452−5p
	hsa−miR−218−5p
	hsa−miR−139−5p
	hsa−miR−143−3p
	hsa−miR−139−3p
	hsa−miR−224−5p
	hsa−miR−126−3p
	hsa−miR−126−5p
	hsa−miR−452−3p
	hsa−let−7c−5p
	hsa−miR−708−3p
	hsa−miR−99a−5p
	hsa−miR−133a−3p
	hsa−miR−1295a
	hsa−miR−99a−3p
	hsa−miR−196a−5p
	hsa−miR−125b−2−3p
	hsa−miR−24−1−5p
	hsa−miR−196b−5p
	hsa−miR−224−3p
	hsa−miR−218−1−3p
	hsa−miR−3651
	hsa−miR−27b−5p
	hsa−miR−585−3p
	hsa−miR−149−5p
	hsa−miR−27b−3p
	hsa−miR−205−5p
	hsa−miR−193b−3p
	hsa−miR−150−5p
	hsa−miR−146b−5p
	hsa−miR−1258
	hsa−miR−369−5p
	hsa−miR−31−3p
	hsa−miR−598−3p
	hsa−miR−125b−5p
	hsa−miR−450a−5p

Cox regression:
logtest pv=7.3e−01
LHR=−0.12 (CI = −0.83, 0.58)

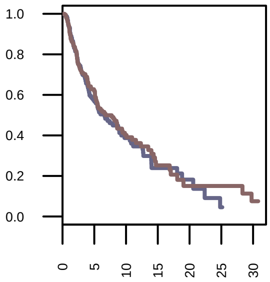

**factor**	**p.value**
Cluster	1.44e−05
Dataset	9.09e−02
Gender	5.15e−01
SampleType	8.37e−01

Cluster
pv=1.4e−05

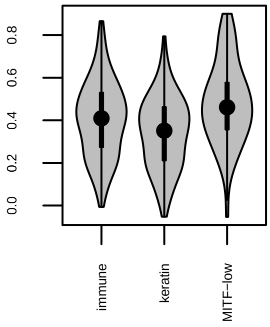

Dataset
pv=9.1e−02

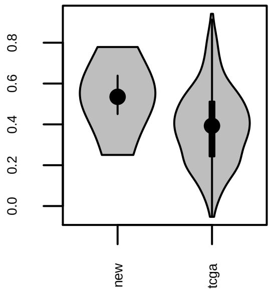

Gender
pv=5.2e−01

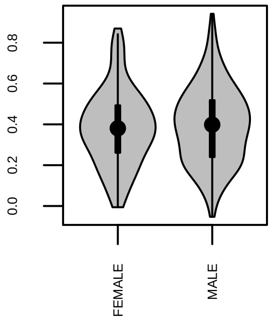

SampleType
pv=8.4e−01

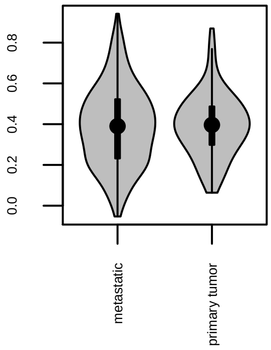

Supplement: Supplementary file 2 — Additional file 2:. Supplementary Results automatic report generated for the discussed components by the presented consensus ICA tool (consICA). (PDF 1107 kb) [file 12920_2019_578_MOESM2_ESM.pdf]
