# Supplementary material for: Deconvolution of transcriptomes and miRNomes by independent component analysis provides insights into biological processes and clinical outcomes of melanoma patients
Source: BMC Med Genomics. 2019 Sep 18;12:132. doi: 10.1186/s12920-019-0578-4 (PMC6751789; doi:10.1186/s12920-019-0578-4)
Supplement: Supplementary file 3 — Additional file 3: Fig. S1. Flow chart of post-processing of ICA results to determine biological relevance of components and networks. Fig. S2. Schematic workflow of ICA application to the discovery, validation and investigation datasets. Fig. S3. Performance of principal component and independent components as feature selection methods. Fig. S4. Similarity between the identified components improves with the increase of number of runs for consensus ICA. Fig. S5. Correlating components to profiles defined in the previous studies. Fig. S6. Pearson correlations between the metagenes of three immune components RIC2, RIC25, RIC57 and one angiogenic component RIC49 on one side, and averaged expression profiles of single cell sub-populations published by Tirosh et al. Fig. S7. Behavior of the important RICs in the validation dataset (same order and RIC naming as in Fig. 6). Fig. S8. STRING networks based on overlapping MIC20-meta-target genes and RIC2 (A), RIC25 (B), RIC27 (C), RIC74 (D) metagenes, showing a significant protein interaction network (medium confidence:0.400, PPI enrichment p-values: < 1.0e-16, < 1.0e-16, < 1.0e-16, and 9.77e-06 accordingly) representing main players within immune response. Fig. S9. STRING networks based on overlapping: MIC22-target genes and RIC13 metagenes (A), MIC25-target genes and RIC13 metagenes (B), MIC25-target genes and RIC49 metagenes (C). Fig. S10. RNA-seq data preparation and metric selection based on discovery TCGA SKCM dataset. Fig. S11. (A) Number of significant positively (red) and negatively (blue) contributing genes in metagene of each of them RNA components before re-orientation. (PDF 2978 kb) [file 12920_2019_578_MOESM3_ESM.pdf]

Supplementary Figure S1

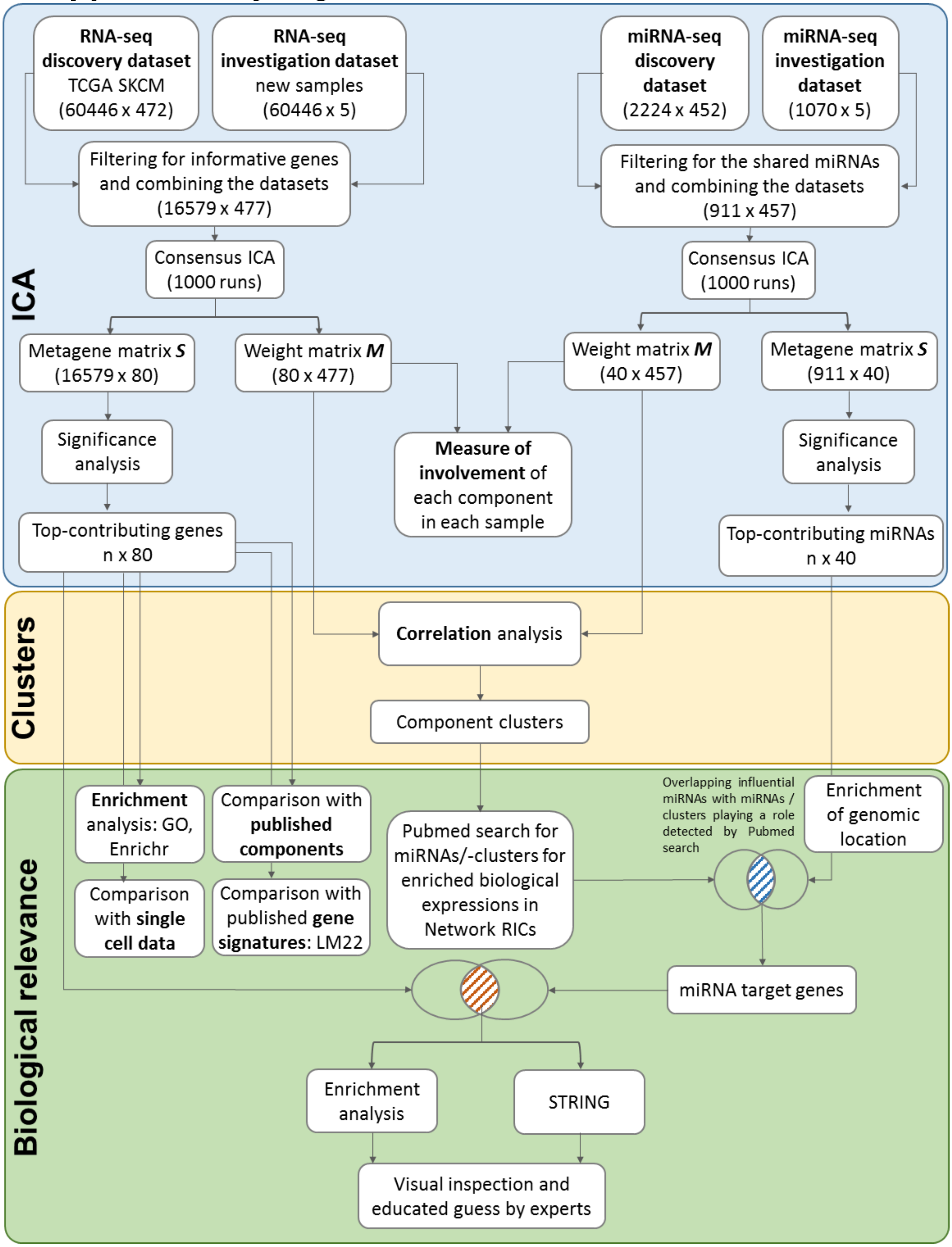

Supplementary Figure S1. Flowchart of post-processing of ICA results to determine biological relevance of components and networks. Two different TCGA SKCM datasets have been analysed (RNA and miRNA-seq data). For both, ICA has been performed resulting in a weight matrix ***M*** (not shown) and a metagene matrix ***S***. Genes strongly involved in ***S*** can be selected by a simple significance test that compares their value in the respective ***S*** matrix to a null distribution of non-influential genes. An enrichment analysis of influential genes of RICs indicates relevant biological processes. To i) integrate MICs and RICs and to ii) investigate biological relevance, a simple correlation analysis was used to build clusters. For those biological processes enriched in clustering RICs, a text-mining search was performed to detect biologically connected miRNAs and clusters. Then, the target genes of influential miRNAs of clustered MICs were overlapped with influential genes of correlated RICs. This overlapped gene-list was submitted to an additional enrichment analysis and STRING.

Supplementary Figure S2

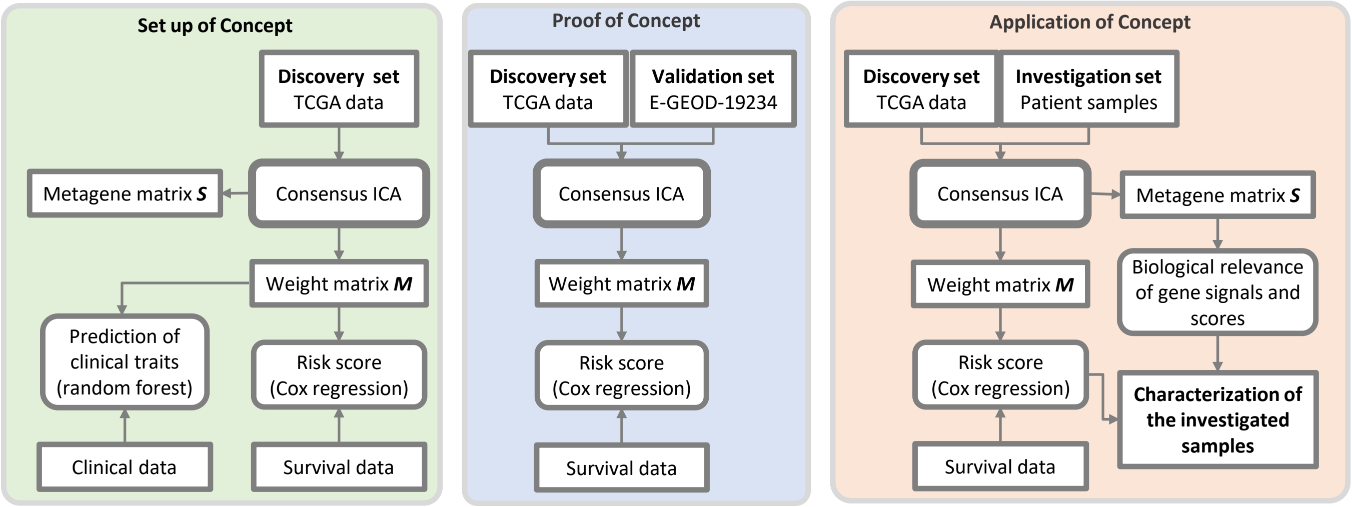

Supplementary Figure S2. Schematic workflow of ICA application to the discovery, validation and investigation datasets. Left panel (green): preliminary ICA of discovery TCGA data. We established the technique, investigated the RNA-seq measures (counts and FPKM), selected the number of components, showed that weight matrix M can be used for patient classification and set up a risk score (RS). Middle panel (blue): the developed risk score was tested on the additional validation dataset. Left panel (red): application of the method on an unpublished investigation dataset of 5 samples: 3 primary tumours, one normal skin and one NHEM cell line. Transcriptome and miRNome data integration and in-depth investigation of the biologically relevant signals seen in S-matrix were performed.

Supplementary Figure S3

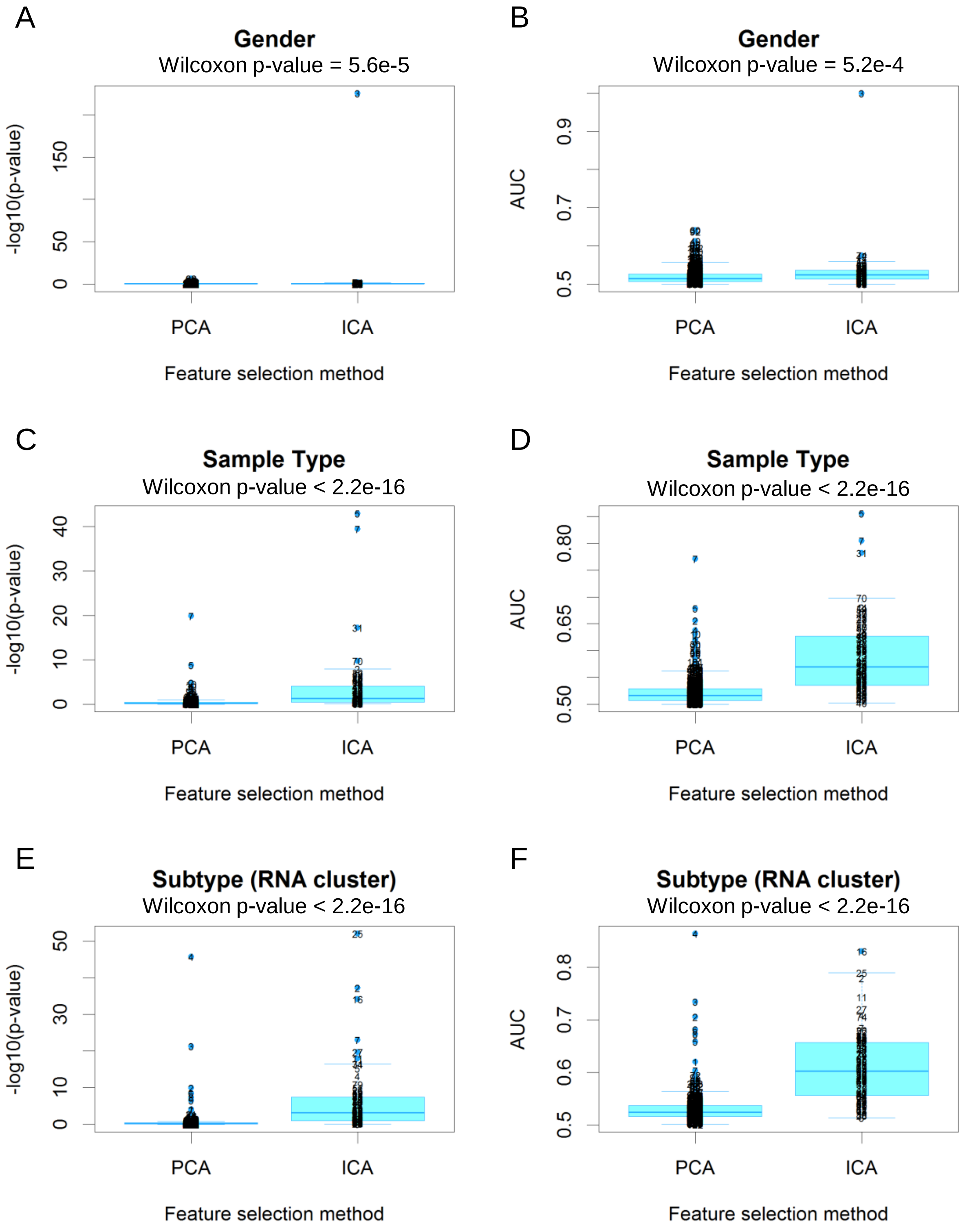

Supplementary Figure S3. Performance of principal component and independent components as feature selection methods. Analysis for discriminating patient gender (A-B), sample type (C, D) and cancer subtype (E, F) was performed for each component separately. We compared ANOVA-based p-values for each component (A,C,E) and AUC (B,D,F). For tumour subtypes we calculated mean AUC of all pairwise comparisons. Component number is shown on the corresponding point.

Supplementary Figure S4

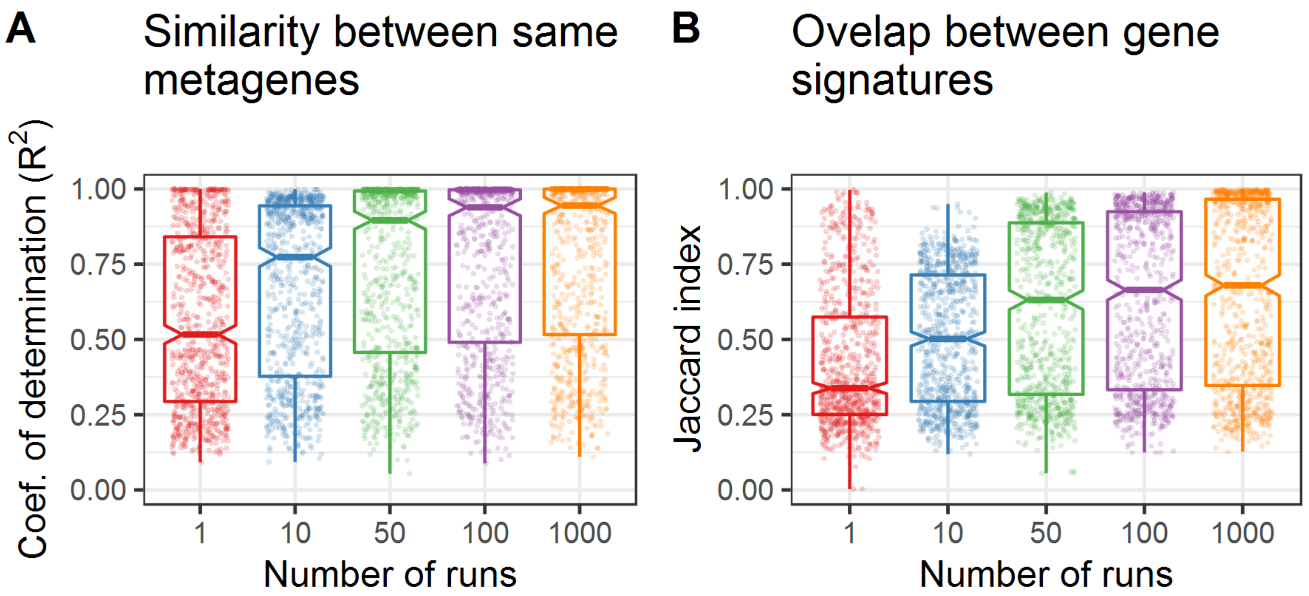

Dunn's test: adjusted p-values for R^2

runs	1	10	50	100	1000
1	1	1.2E-07	1.3E-32	2.0E-48	3.1E-65
10	1.2E-07	1	5.5E-11	1.3E-20	6.4E-32
50	1.3E-32	5.5E-11	1	6.7E-03	2.5E-07
100	2.0E-48	1.3E-20	6.7E-03	1	1.4E-02
1000	3.1E-65	6.4E-32	2.5E-07	1.4E-02	1

Dunn's test: adjusted p-values for Jaccard index

runs	1	10	50	100	1000
1	1	2.0E-07	3.5E-32	1.3E-47	2.1E-60
10	2.0E-07	1	5.4E-11	2.2E-20	5.3E-29
50	3.5E-32	5.4E-11	1	2.2E-20	4.7E-06
100	1.3E-47	2.2E-20	2.2E-20	1	5.4E-02
1000	2.1E-60	5.3E-29	4.7E-06	5.4E-02	1

Supplementary Figure S4. Similarity between the identified components improves with the increase of number of runs for consensus ICA. Here 5 replicating analyses were performed for each number of runs: 1,10, 50, 100 and 1000. In (A), the similarity is measured as the coefficient of determination (R^2) between profiles of the contributing genes, i.e columns of matrix S (only pairs of components with highest R^2 were considered). In (B), the lists of significantly contributing genes were compared by Jaccard index. We checked significance of the number of components using Krushkal-Wallis test (in both cases p-value < 2.2e-16) and applied Dunn's post-hoc test with Benjamini-Hochberg's correction (adj.p-values < 0.01 are highlighted in Tables). Here we used nonparametric tests because of bi-modal distribution of calculated R^2 and Jaccard indexes.

Supplementary Figure S5

A

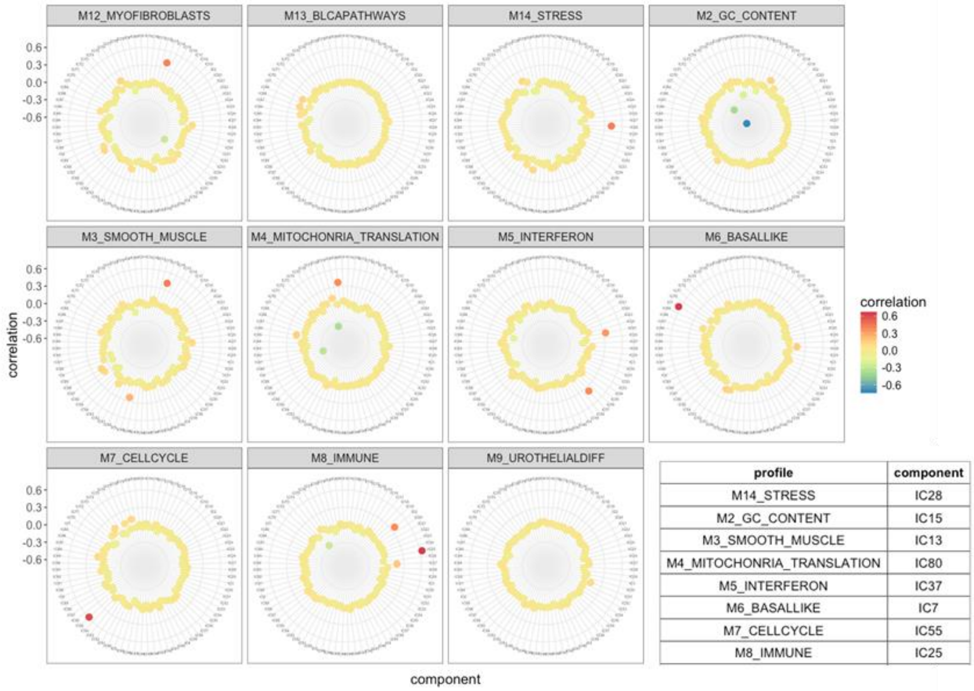

profile	component
M14_STRESS	IC28
M2_GC_CONTENT	IC15
M3_SMOOTH_MUSCLE	IC13
M4_MITOCHONRIA_TRANSLATION	IC80
M5_INTERFERON	IC37
M6_BASALLIKE	IC7
M7_CELLCYCLE	IC55
M8_IMMUNE	IC25

B

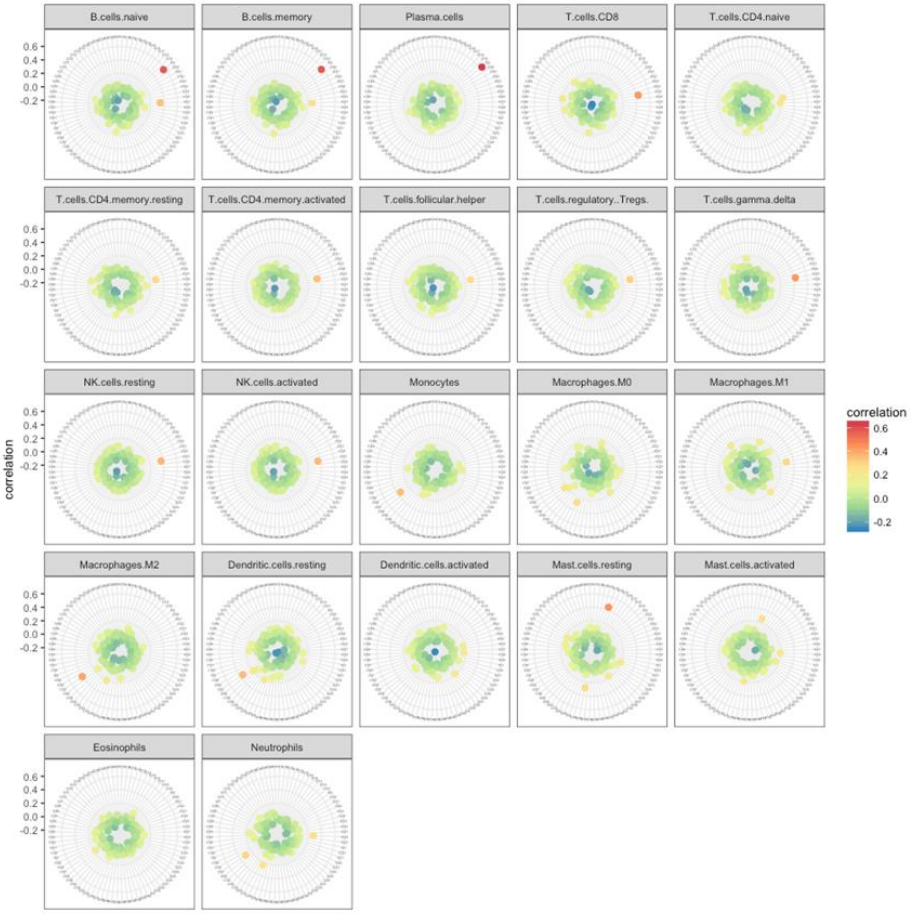

TYPE	IC	r	p.val
Plasma.cells	IC2	0.6345223	0.000000e+00
B.cells.memory	IC2	0.5772912	0.000000e+00
B.cells.naive	IC2	0.5722786	0.000000e+00
Mast.cells.resting	IC13	0.4438004	0.000000e+00
T.cells.CD8	IC25	0.4378903	0.000000e+00
T.cells.gamma.delta	IC25	0.4311936	0.000000e+00
Macrophages.M2	IC57	0.4052127	0.000000e+00
NK.cells.resting	IC25	0.3680599	1.798561e-14
NK.cells.activated	IC25	0.3582026	9.769963e-14
Dendritic.cells.resting	IC57	0.3533929	2.189360e-13
Monocytes	IC57	0.3492959	4.294343e-13
T.cells.CD4.memory.activated	IC25	0.3450473	8.557599e-13
T.cells.regulatory..Tregs.	IC25	0.3116811	1.349472e-10
Neutrophils	IC57	0.3005246	6.403389e-10
Macrophages.M1	IC25	0.2934334	1.665341e-09
T.cells.CD4.memory.resting	IC25	0.2882322	3.302200e-09
T.cells.follicular.helper	IC25	0.2830928	6.407385e-09
Macrophages.M0	IC50	0.2801740	9.280856e-09
Mast.cells.activated	IC13	0.2625065	7.984905e-08
T.cells.CD4.naive	IC25	0.2416493	8.337452e-07
Dendritic.cells.activated	IC37	0.1832383	2.054304e-04
Eosinophils	IC57	0.1673730	7.092196e-04

Maximal correspondence

Supplementary Figure S5. Correlating components to profiles defined in the previous studies. (A) Reciprocally correlated metagenes of components found within this study and previously identified for bladder tumours by Biton et al. (B) Correlations between metagenes of the identified components and leukocyte gene signatures LM22, published by Newman et al.

Supplementary Figure S6

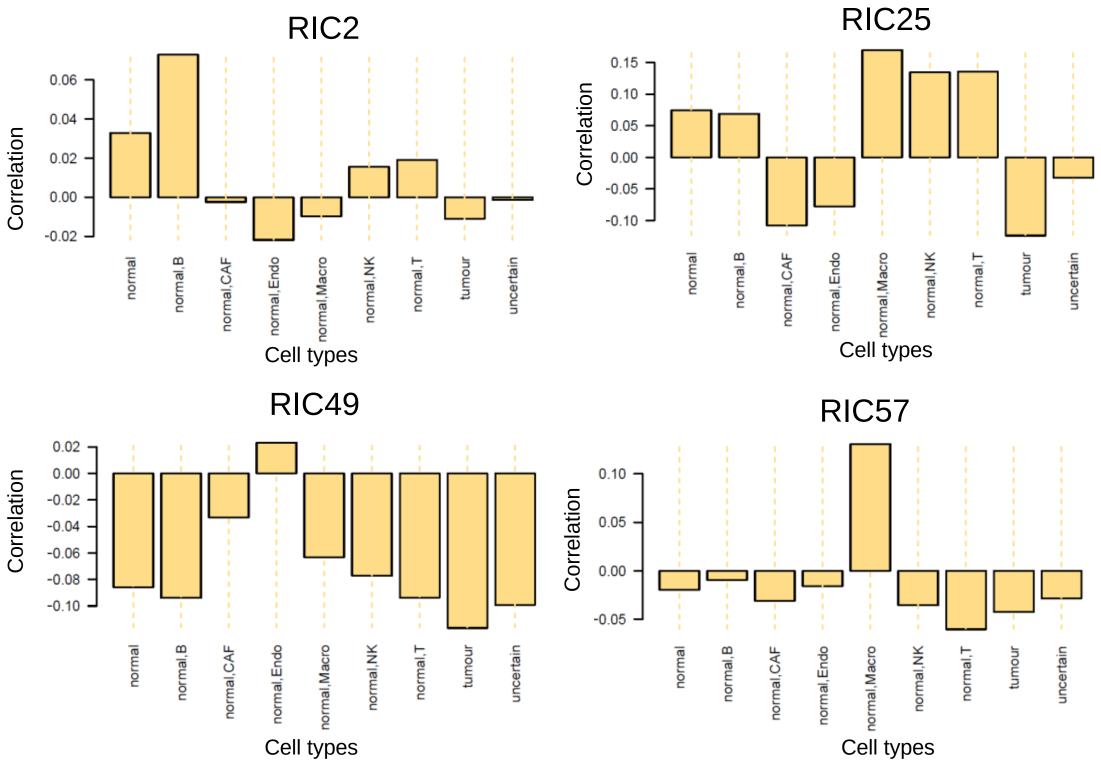

Supplementary Figure S6. Pearson correlations between the metagenes of three immune components RIC2, RIC25, RIC57 and one angiogenic component RIC49 on one side, and averaged expression profiles of single cell sub-populations published by Tirosh et al.

Supplementary Figure S7

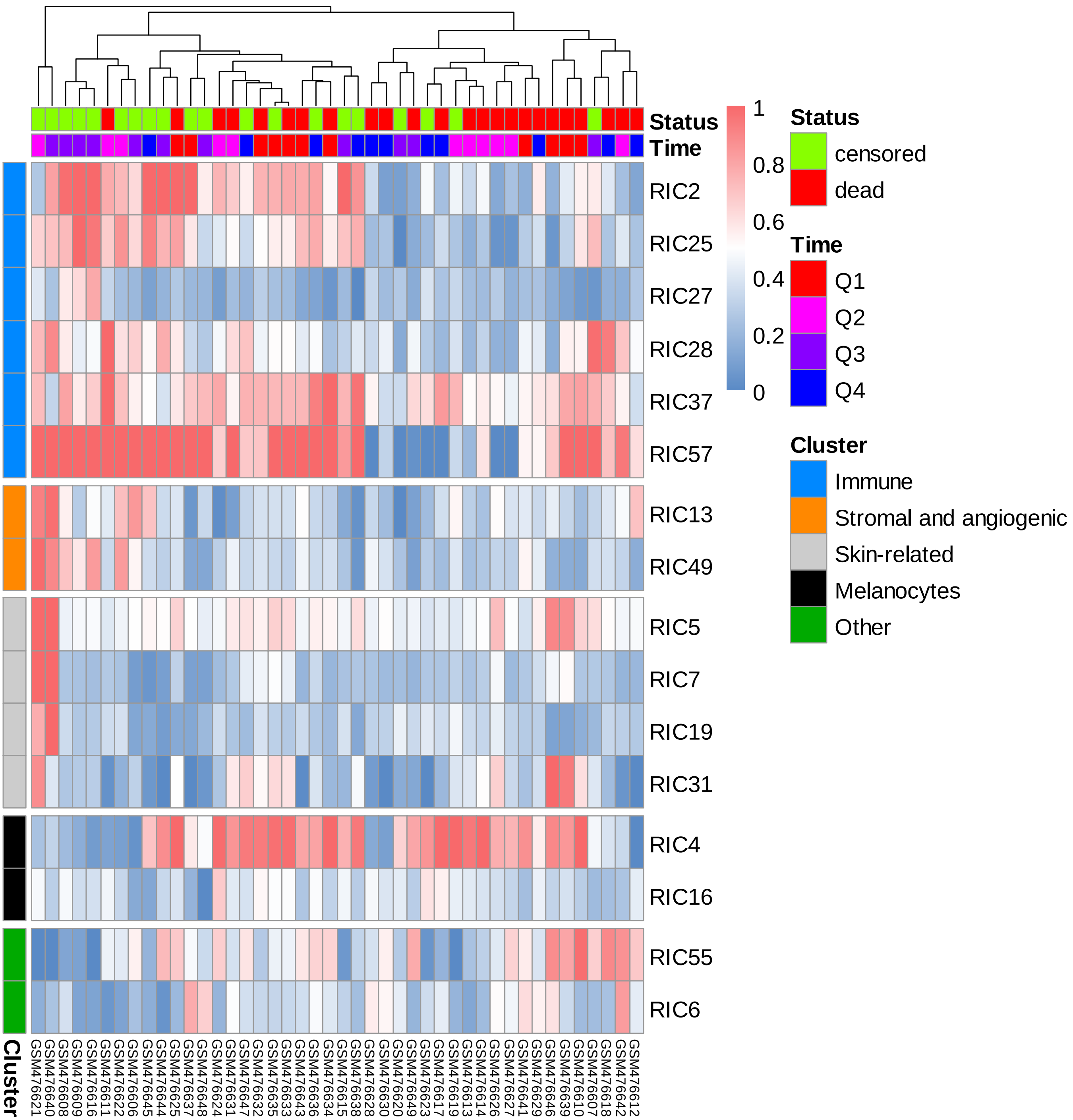

Supplementary Figure S7. Behavior of the important RICs in the validation dataset (same order and RIC naming as in Fig.6). Survival times were categorized and are represented by the corresponding quartiles (Q1-Q4) on the top of the heatmap.

Supplementary Figure S8

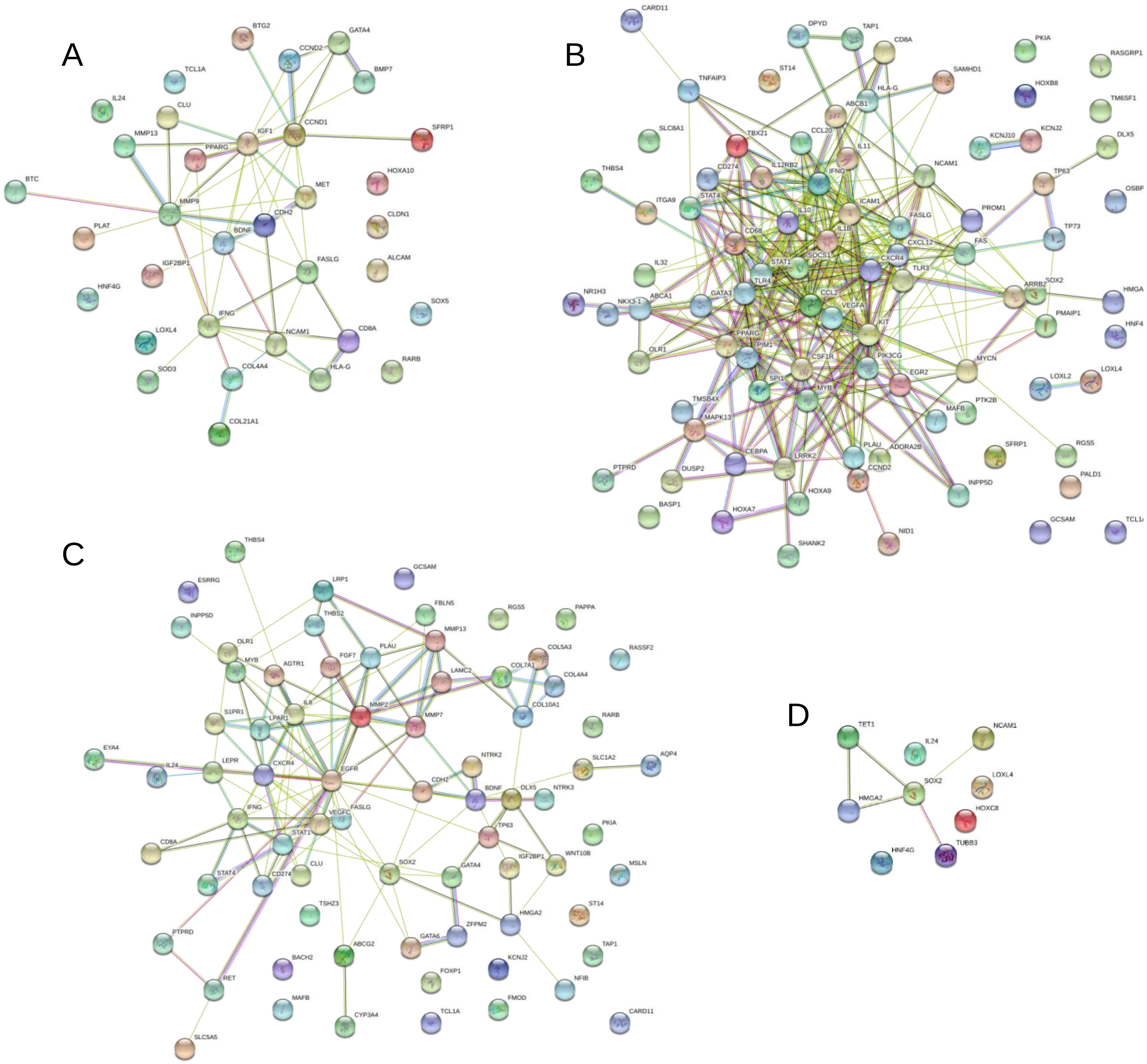

Supplementary Figure S8. STRING networks based on overlapping MIC20-meta-targetgenes and RIC2 (A), RIC25 (B), RIC27 (C), RIC74 (D) metagenes, showing a significant protein interaction network (medium confidence: 0.400, PPI enrichment p-values: <1.0e-16, <1.0e-16, <1.0e-16, and 9.77e-06 accordingly) representing main players within immune response. The gene list uploaded to STRING represents the overlap between the target genes of influential miRNAs of MIC20 which are found in immune response, respectively B- and T-cell, related publications and influential genes of considered RICs (see Supplementary Figure S1, red overlap in green box for biological relevance).

Supplementary Figure S9

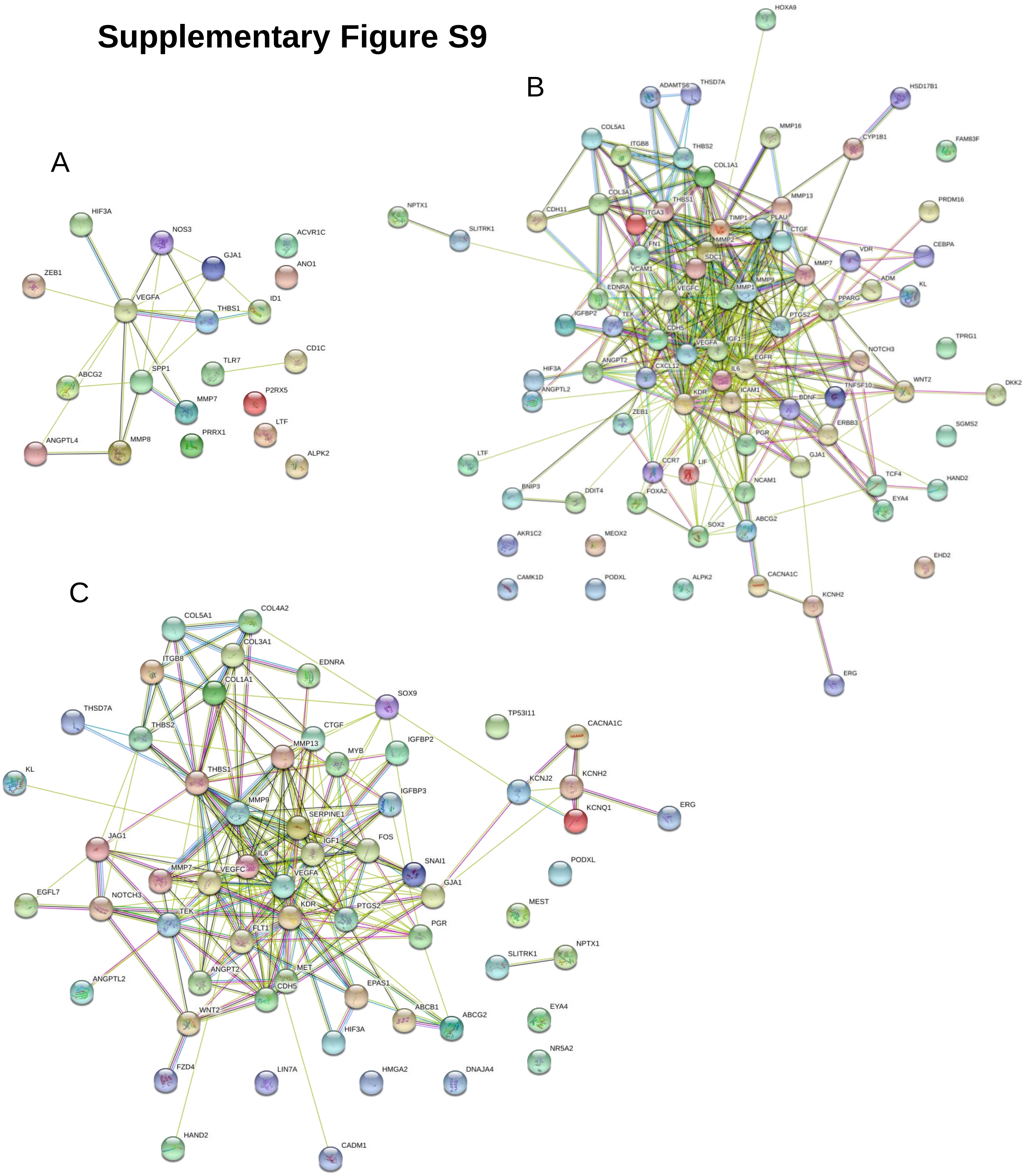

Supplementary Table S9. STRING networks based on overlapping: MIC22-target genes and RIC13 metagenes (A), MIC25-target genes and RIC13 metagenes (B), MIC25-target genes and RIC49 metagenes (C). Significant protein interaction networks (medium confidence: 0.400, PPI enrichment p-values: 1.27e-13, <1.0e-16, <1.0e-16 respectively) are observed, representing main players within angiogenesis. The gene list uploaded into STRING represents the overlap between the target genes of top-contributing miRNAs of MIC22 and MIC25 found in angiogenesis-related publications and top-contributing genes of RIC13 and RIC49 (also see Supplementary Figure S1, red overlap in the green box for biological relevance).

Supplementary Figure S10

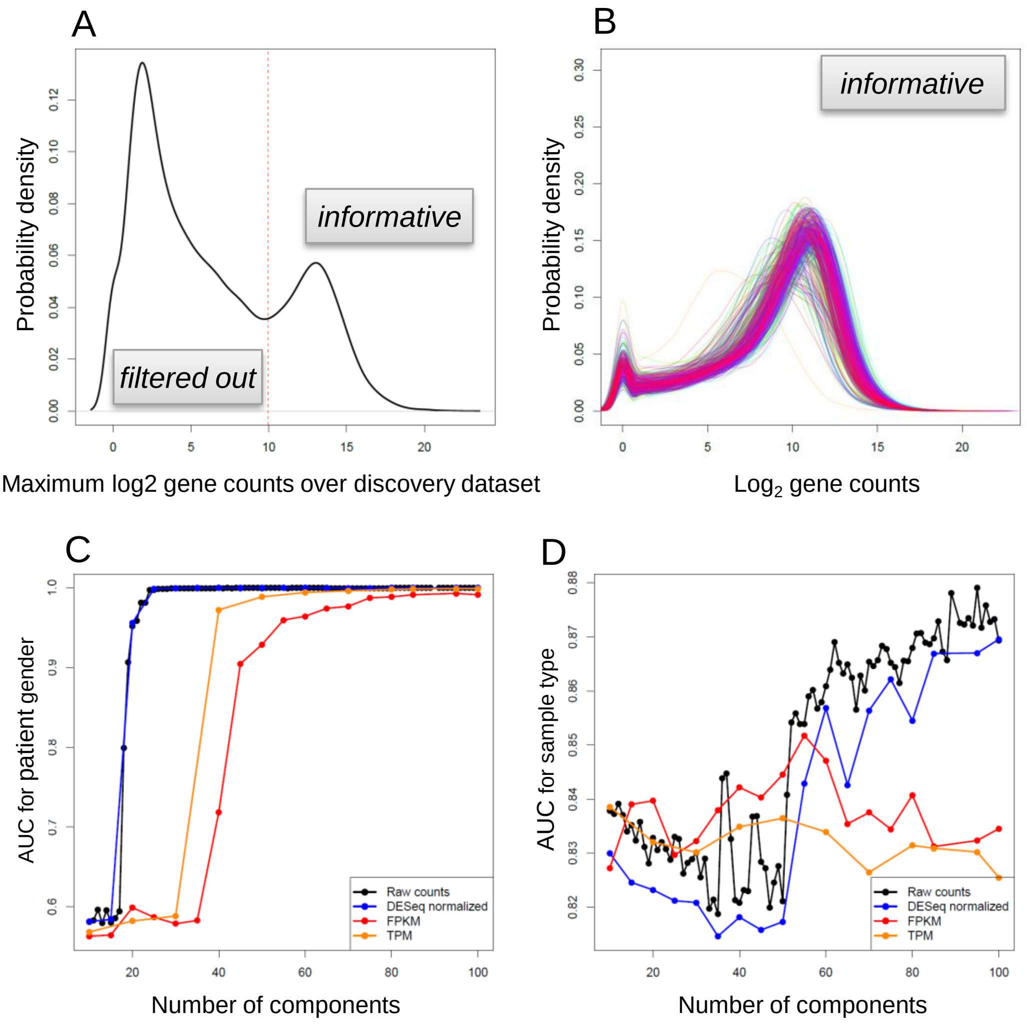

Supplementary Figure S10. RNA-seq data preparation and metric selection based on discovery TCGA SKCM dataset. (A) Distribution of maximum log2 expression over all discovery samples for the genes with soft filtering threshold (red dotted line). Genes which are below the threshold in all samples are filtered out, and the rest are considered as informative. (B) Distribution of log expression for the informative genes after filtration. The best AUC from ICA weight matrix, when classifying patient gender (C) and sample type (primary / metastatic) (D). In both cases raw counts showed higher AUC values with a lower number of independent components.

Supplementary Figure S11

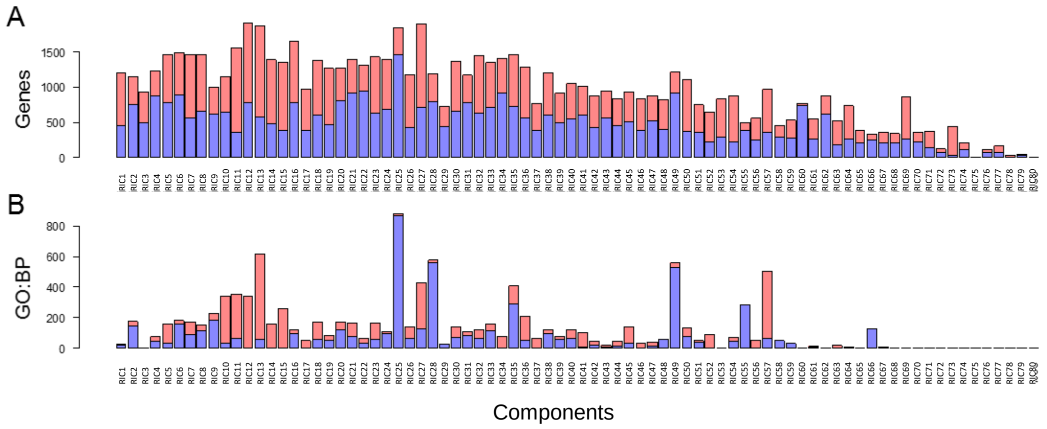

Supplementary Figure S11. (A) Number of significant positively (red) and negatively (blue) contributing genes in metagene of each of the mRNA components before re-orientation. (B) Number of enriched GO biological processes found for these genes. In the most cases, only one list of genes is biologically meaningful: either positive (e.g. RIC10-RIC15) or negative (e.g. RIC25, RIC28, RIC49, RIC55). The components were reoriented to ensure that top-contributing genes with the most significant GO terms are positive.
